# Settling Velocities of Tire and Road Wear Particles: Analyzing Finely Graded Density Fractions of Samples from a Road Simulator and a Highway Tunnel

**DOI:** 10.1021/acs.est.5c04165

**Published:** 2025-06-18

**Authors:** Stefan Dittmar, Steffen Weyrauch, Thorsten Reemtsma, Paul Eisentraut, Korinna Altmann, Aki S. Ruhl, Martin Jekel

**Affiliations:** † Chair of Water Quality Control, 26524 Technische Universität Berlin, Straße des 17. Juni 135, 10623 Berlin, Germany; ‡ GEOMAR Helmholtz Centre for Ocean Research Kiel, Wischhofstraße 1−3, 24148 Kiel, Germany; § Department of Analytical Chemistry, Helmholtz-Centre for Environmental Research−UFZ, Permoserstrasse 15, 04318 Leipzig, Germany; ∥ Bundesanstalt für Materialforschung und -prüfung (BAM), Unter den Eichen 87, 12205 Berlin, Germany; # German Environment Agency (UBA), Section II 3.3, Schichauweg 58, 12307 Berlin, Germany

**Keywords:** transport, sinking velocity, sedimentation, particle tracking, TED-GC/MS, microplastics

## Abstract

The terminal settling velocity is considered the most critical parameter determining the transport of tire and road wear particles (TRWP) in aquatic environments. Nonetheless, no respective empirical data has been reported so far. In this study, particle samples from a road simulator and a highway tunnel were investigated with a validated imaging method. Different density and size fractions of both samples were measured separately, acquiring sizes and settling velocities of more than 30,000 individual particles. In addition, tire marker polymers were analyzed for each fraction via thermal extraction desorption-gas chromatography/mass spectrometry. Finally, the acquired particle data was combined according to the fractions’ estimated tire contents in order to deduce detailed probability distributions of particle size and settling velocity for the actual TRWP from both samples. Weighted by TRWP-incorporated tire mass, median diameters of 54 and 44 μm as well as median settling velocities of 0.65 and 0.22 mm/s were found for TRWP from the road simulator and highway tunnel, respectively. This study thus provides the first ever empirical data on TRWP settling velocities in water, which can be highly valuable input for modeling the environmental transport of TRWP and for dimensioning TRWP retention systems.

## Graphical abstract

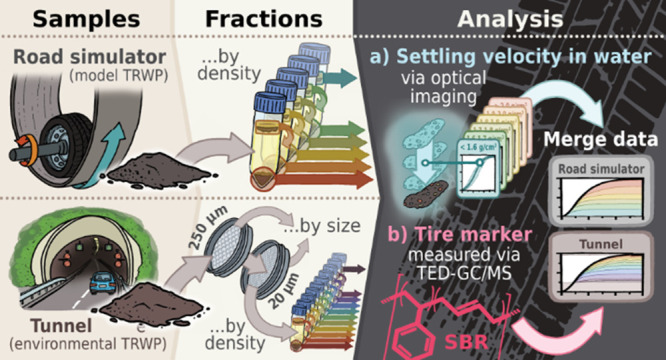

## Introduction

Tire and road wear particles (TRWP) are generated by abrasion of tire tread due to road friction. TRWP are thus emitted in high numbers everywhere where vehicles drive. Modeling revealed average generation rates as high as 0.1 g/km per car and 1 g/km per truck,^1^ which amount to estimated global annual emissions of 5.9 Mt/a.^2^ For Germany, a calculation suggests that 66–76% of TRWP end up in road-side soil, while 12–20% are transported to surface waters.^3^ Even with partly divergent presumptions, another study concluded similar percentages for Switzerland.^4^ If subsumed as microplastics (MPs),^5^ TRWP are considered one of the biggest contributors to overall MP emissions.^1,2,6^ Recently, poisoning and death of wildlife has been linked to tire constituents and respective transformation products.^7–9^

The major pathway of TRWP into the aquatic environment is road runoff by stormwater.^3^ TRWP accumulate on the road pavement until they are washed off and, if not caught by retention systems, are received by surface waters.^10^ Airborne transport is not addressed in this study, yet is currently supposed to affect less than 10% of overall TRWP.^3,6,11^

Within water bodies, gravitational settling is a key process influencing the particles’ vertical transport and consequently the environmental fate of TRWP as well as possible hotspots or accumulation zones.^12–14^ Modeling studies identified the settling velocity as one of the most important and sensitive input parameters, e.g. when describing riverine transport and potential ocean input of TRWP.^15,16^ Yet, no empirical data is available on the terminal settling velocities of TRWP in water so far, which is why respective models have to rely on purely theoretical assumptions and estimates.^15–17^ Other influential factors, such as convection, aggregation, or biofouling, are usually interlinked but implemented separately in transport models and are therefore not considered in this study.

The settling velocity of a particle in water is determined by its size, shape and density. TRWP consist of abraded tire rubber and incorporated debris from the road surface or surroundings.^18^ The resulting particles are heterogeneous and variable in density.^19^ TRWP are mostly characterized as elongated, rod-shaped particles ranging from tens of nm to hundreds of μm in size.^3,6,20^

One of the major obstacles in TRWP research is to obtain representative samples. Particle samples from road surfaces not only contain TRWP of different tire product origin and aging history, but possibly a plethora of other particulates as well, e.g. other traffic residues such as brake wear or environmental debris.^18,21^

This study provides detailed measurements of TRWP particle size and settling velocities according to two different particle samples: First, model TRWP from a road simulator were investigated. These are generated to serve as a potential reference material for research on TRWP. Second, road dust sampled from a highway tunnel was examined, because it presumably contains high amounts of TRWP as they are emitted to and found in the environment. Since the samples contain TRWP of various density and sizes and likely include non-TRWP as well, a combination of different fractionation, analysis and data processing methods was chosen to elucidate TRWP sizes and settling velocities.

## Materials and Methods

### Analytical Strategy

Figure 1 depicts an overview of this study’s research approach. The scheme includes all relevant analyses and data processing steps. To provide further guidance, the intermediate and final results are shown as sketches of the subsequent figures.

**Figure 1:**
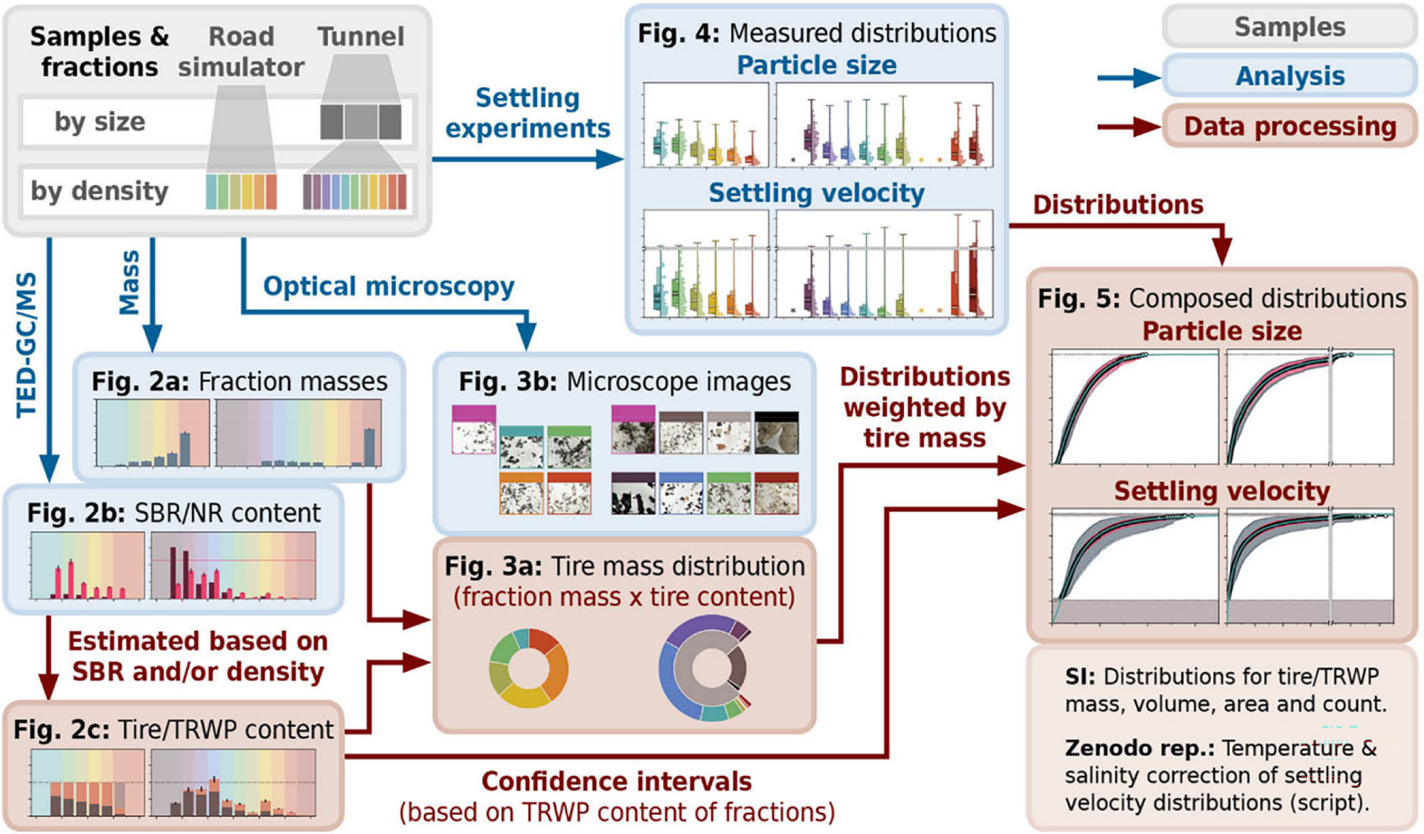
Flow diagram of this study’s structure, including samples, analysis methods, and data processing steps (see legend on the upper right) in order to elucidate TRWP size and settling velocities in water. Sketches of all subsequent figures are integrated as they display intermediate and final results.

Initially, both samples were fractionated by density (and by size in case of the tunnel dust) in order to enrich TRWP in certain fractions (cf. Figure 2a). The fractions’ contents of the tire marker polymers styrene–butadiene rubber (SBR) and natural rubber (NR) were analyzed via thermal extraction desorption-gas chromatography/mass spectrometry (TED-GC/MS, cf. Figure 2b).^22^ The tire and TRWP contents of each fraction were then estimated based on measured SBR contents and average fraction densities (cf. Figure 2c) and qualitatively supported by optical microscopy (cf. Figure 3b). Conclusively, the distribution of tire mass across both particle samples could be evaluated (cf. Figure 3a).

**Figure 2:**
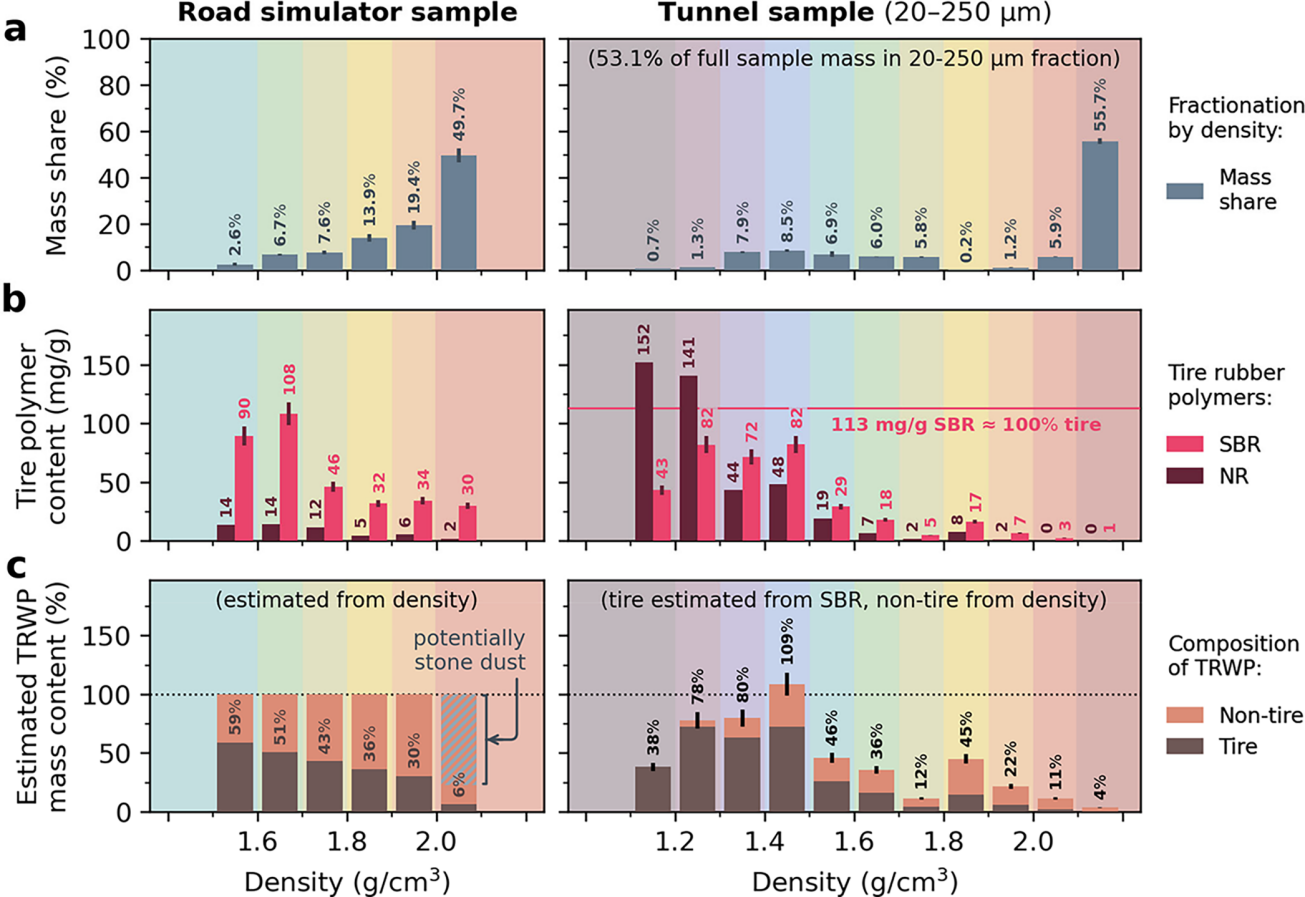
Results of the density fractionations of the Road simulator sample (left side) and the 20–250 μm size fraction of the Tunnel sample (right side). The density fractions’ (a) relative shares of total sample mass, (b) measured contents of the tire marker polymers SBR and NR and (c) estimated TRWP mass contents are depicted. TRWP mass contents are shown as the sum of tire and nontire material, e.g., mineral encrustations, as estimated from SBR content and average fraction density. For the Tunnel sample, 113 mg/g SBR is indicated in (b) as the global average SBR content of tire tread according to Eisentraut et al.^22^

**Figure 3:**
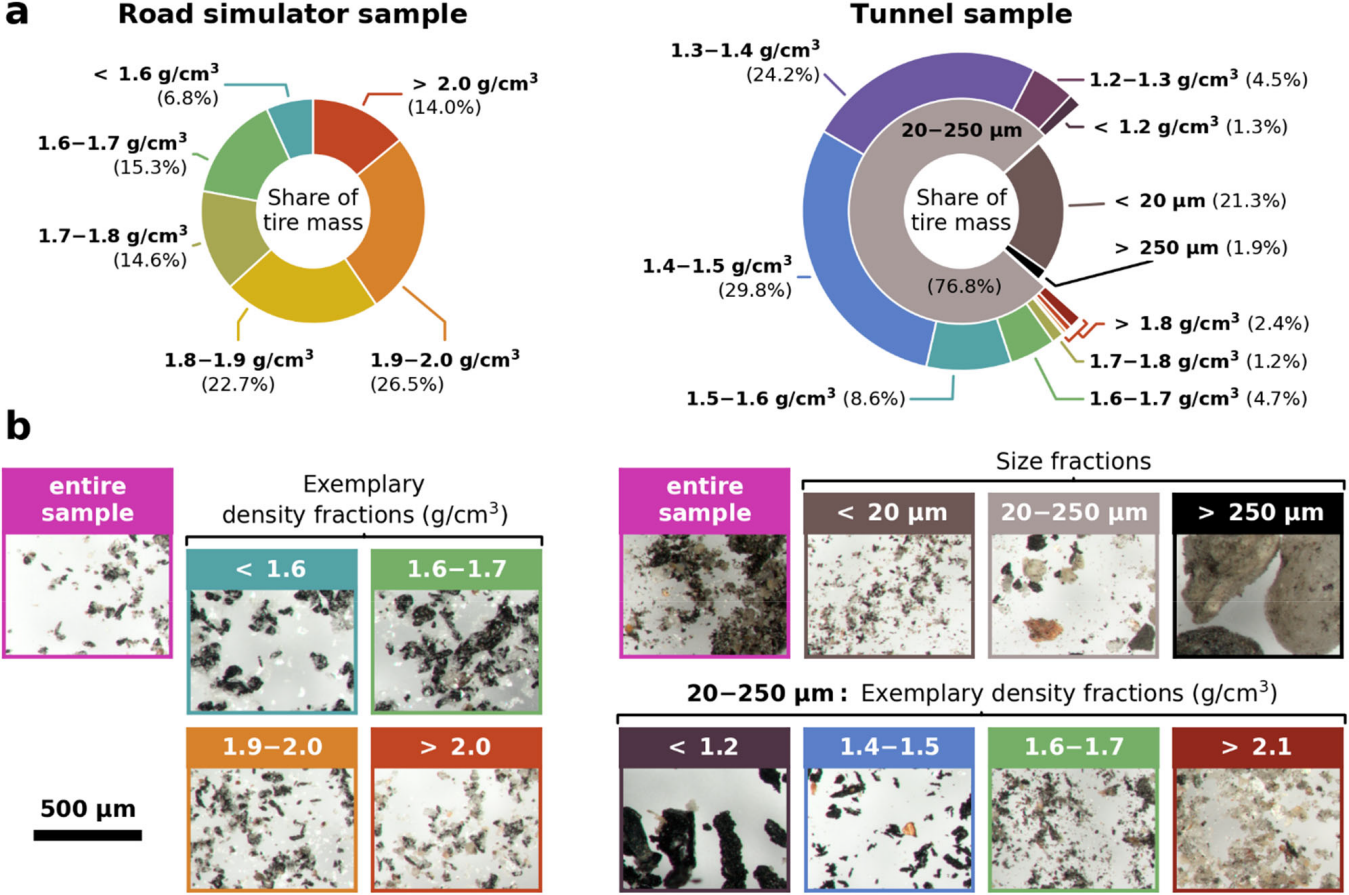
(a) Distribution of tire mass across the obtained fractions of the Road simulator sample (density fractions, left side) and the Tunnel sample (density and size fractions, right side) as well as (b) microscope images of both particle samples and several exemplary fractions. A uniform scale bar is indicated on the bottom left.

Complimentarily, comprehensive series of settling experiments in water were conducted for the different fractions using a previously validated measuring setup.^23^ Distributions of particle size and settling velocity were obtained for each fraction via optical imaging and subsequent tracking of individual particles (cf. Figure 4). These results are recombined with respect to the fractions’ supposed tire and TRWP content in order to finally derive distributions of particle sizes and settling velocities for TRWP from both samples (cf. Figure 5). In the following, all mentioned methods are described in detail.

**Figure 4:**
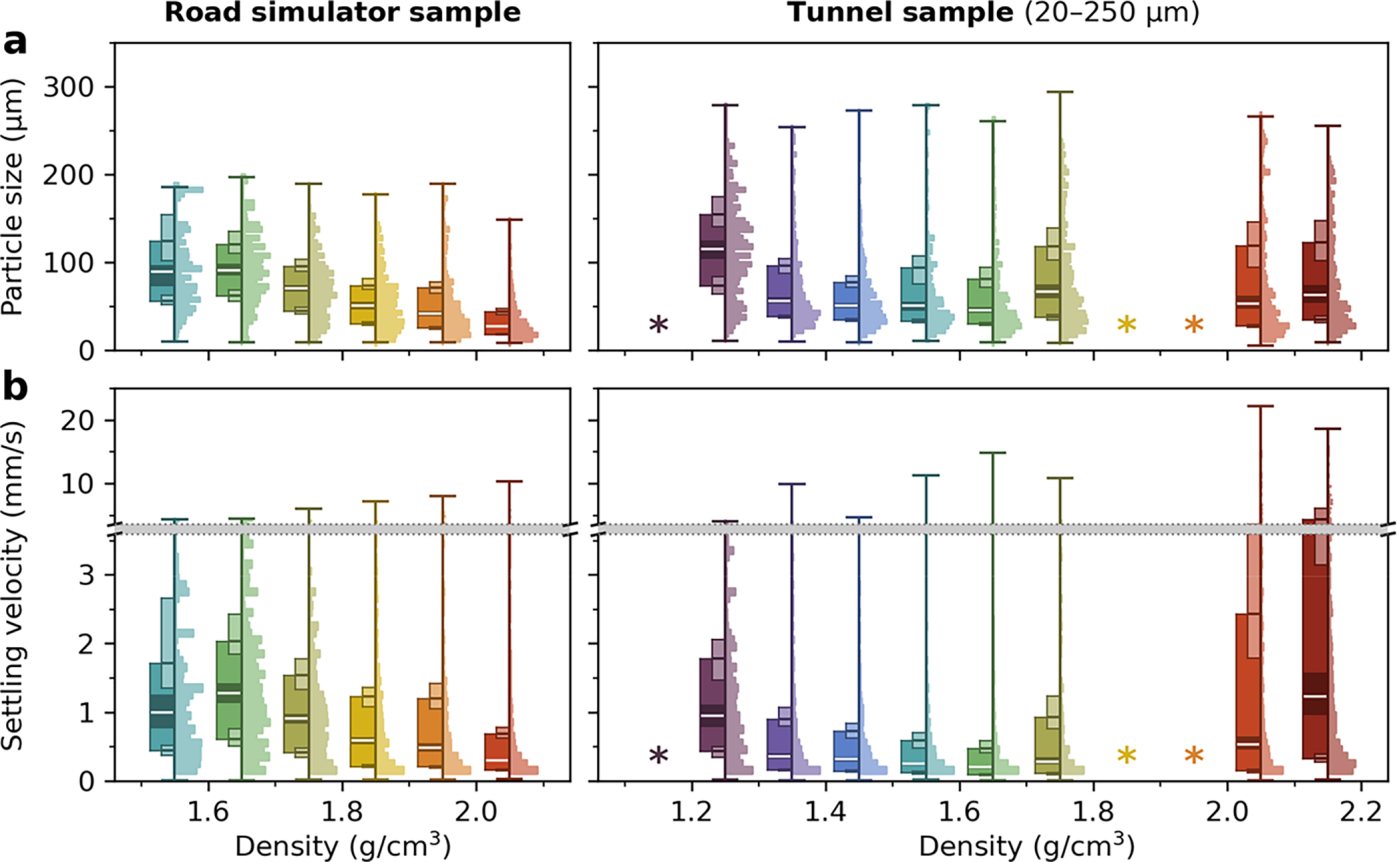
Distributions of (a) particle sizes and (b) settling velocities (in water at 15 °C, mind the broken *y*-axis) as measured in settling experiments for the different density fractions of the Road simulator sample (left side) and of the 20–250 μm size fraction of the Tunnel sample (right side). Shown distributions are weighted by particle volume and depicted as dual plots that include a histogram (right-hand half) and a boxplot with range, quartiles and associated 95% confidence intervals (left-hand half). Density fractions that were not analyzed due to insufficient sample mass are annotated (*).

**Figure 5:**
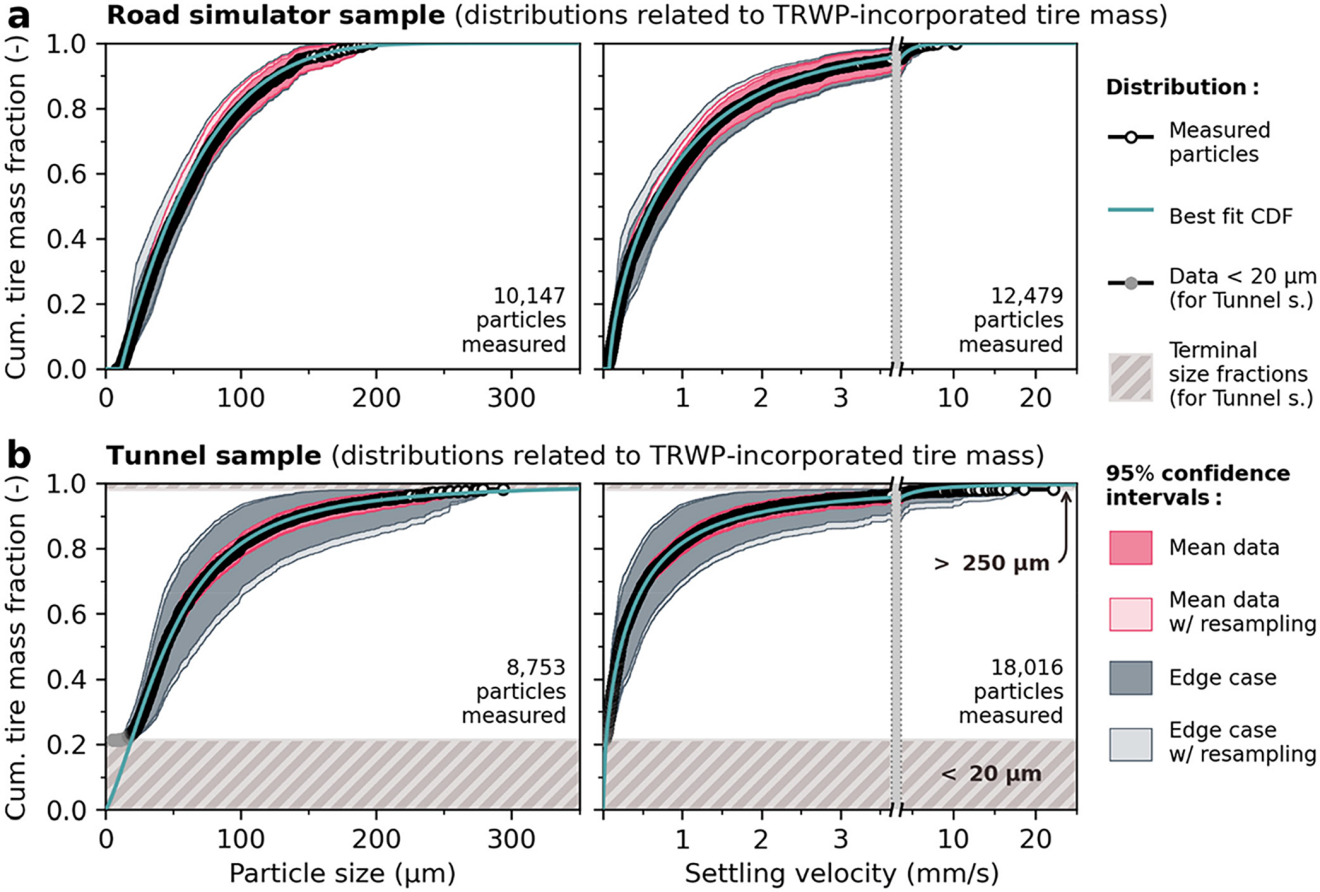
Particle sizes (left side) and settling velocities (measured in water at 15 °C, right side, mind the broken *x*-axis) of TRWP from (a) the Road simulator sample and (b) the Tunnel sample. The cumulative distributions are weighted by TRWP-incorporated tire mass and were composed from data on individual density fractions of both samples. Best fit cumulative distribution functions (CDF) are included, respectively (also see Table S9). 95% confidence intervals (CIs) were derived from the mean 95% CIs of the settling data on the different density fractions. The edge case 95% CI is computed by associating the respective TRWP content to the slowest/fastest or smallest/largest particles of a density fraction. Mean densities, mass shares, and tire contents of the density fractions were resampled (*N* = 2000) to derive extended CIs. Lower and upper size fractions of the Tunnel sample were not investigated but are annotated and assumed to have the lowest or highest sizes and velocities, respectively.

### Samples and Investigated Fractions

The Road simulator sample (containing model TRWP) was generated by Karlsruhe Institute for Technology (KIT) at a tire test bench consisting of a rotating drum lined with asphalt on the inside. During operation of the simulator, 15 g of stone dust (crushed moraine, milled to 1.0–5.8 μm) was added once every 1.5 h to support abrasion of tire tread. Further information is provided elsewhere.^24^ The obtained particle sample was sieved to below 150 μm prior to provision and was characterized before with respect to the morphology and elemental composition of individual particles.^24^

In this study, sequential density fractions with a step size of 0.1 g/cm^3^ were investigated. Consecutive steps of suspension and separation in respective sodium polytungstate solutions were performed to obtain six fractions of the Road simulator sample (terminal fractions: <1.6 g/cm^3^ and >2.0 g/cm^3^). Details are provided in Section S1 of the Supporting Information.

The Tunnel sample (containing environmental TRWP) was obtained from the German highway tunnel “Königshainer Berge” (51.2148°, 14.8119°) on January 21, 2020. Road dust was sampled using a pressure washer. Since its completion in 1999, the tunnel had only been dry swept (twice a year). In 2019, according to the counting station “Nieder Seifersdorf” (51.2078°, 14.7889°) in immediate vicinity, the average traffic volume within the sampled tunnel tube amounted to 10,607 cars and 4925 heavy goods vehicles per day.^25^ Further details on sampling location, technique and sample processing have been described by Klöckner et al.^21^ They denoted the Tunnel sample as Sample A in their original publication and already reported peak concentrations of 6PPD and tire-related Zn for the 20–200 μm size fraction and the 1.3–1.5 g/cm^3^ density fraction, respectively.^21^

For this study, particles below 20 μm and above 250 μm in size were separated beforehand via dry-sieving the Tunnel sample. Then, 11 density fractions were exclusively obtained from the 20–250 μm size class (in 0.1 g/cm^3^ steps, terminal fractions: <1.2 g/cm^3^ and >2.1 g/cm^3^, cf. Section S1).

Both samples and all generated fractions were examined with a stereo microscope (SMZ-1270, Nikon, Japan) equipped with a camera for capturing digital images (SC50, Olympus, Japan). Average densities were determined experimentally for the upper terminal density fractions of both samples, for the stone dust added to the road simulator, as well as for the three size fractions of the Tunnel sample. A pycnometer (2 mL) was used to measure density via displacement of isopropanol (see Section S2 for details and results).

### Settling Velocity Measurements

Single particle settling velocities were measured in quiescent water using a setup and method validated for small MPs.^23,26^ Stock suspensions containing the target particles and a surfactant mix (NovaChem SF100, Postnova Analytics, Germany) at equal concentrations (10–40 mg/L, cf. Table 1) were kept at 30 °C in a water bath (1092, GFL, Germany).^23^ Prior to an experimental run, the suspension was homogenized by shaking (20 s) before immediately transferring 10 mL to the precisely tempered settling column (14.99 ± 0.04 °C). Images of settling particles were acquired within a fixed field of view (FOV, 3.55 × 4.24 mm) in the lower part of the column. The initial frame rate (annotated in Table 1) was incrementally reduced with progressing runtime of an experiment as described previously.^23^ Image sequences were processed with a tracking algorithm^27^ to obtain velocities, trajectories and contours of observed particles. To be detected, a particle’s contour size has to exceed a minimum equivalent circular diameter (ECD) of 10 μm. Throughout this study, particle size is otherwise characterized as the corrected equivalent diameter *d*_eq_ proposed by Bagheri et al.,^28^ which was used to estimate particle volume and surface area, too (cf. Section S3). Full methodical details are provided in previous publications.^23,26^

**Table 1:** Sets of Settling Experiments Conducted for the Density Fractions of the Given Sample

sample name	set	runs	particle dose per run (mg)^a^	runtime
Road simulator sample (six fractions analyzed)	Set 1^b^	20	0.1	1 h 30 min
	Set 2	40	0.2	8 min 30 s^d^
	Set 3	80^c^	0.4	4 min 15 s
Tunnel sample (eight fractions analyzed)	Set 1^b^	4	0.1	4 h
	Set 2^b^	16	0.1	1 h 30 min
	Set 3	40	0.2	12 min 45 s
	Set 4	160^c^	0.4	4 min 15 s

a To achieve the indicated particle dose, 10 mL of a 10, 20, or 40 mg/L particle stock suspension were dosed at the beginning of each run.

b Image acquisition at an initial frame rate of 10 Hz instead of 20 Hz.

c Halved for <1.6 and 1.2–1.3 g/cm^3^ fractions of the Road simulator sample and Tunnel sample, respectively, due to low fraction mass.

d Increased to 25 min for fraction <1.6 g/cm^3^ fraction of the Road simulator sample.

Settling velocities were measured for all density fractions of both samples, except for the fractions <1.2, 1.8–1.9, and 1.9–2.0 g/cm^3^ of the Tunnel sample due to insufficient quantity of these density classes. As summarized in Table 1, several sets of settling experiments were conducted, respectively. To gather data over the entire range of particle sizes and velocities, higher numbers of runs with decreased runtime were performed. Thus, an increased amount of larger particles is observed, which are usually less frequent and more difficult to characterize in terms of size.^26,28^

Based on the acquired data on individual particles, representative distributions of particle sizes and settling velocities can be derived—either related to particle volume, surface area, or count. Here, the data from different sets of experiments is combined by introducing appropriate cutoffs for size or velocity to account for different runtimes. This procedure is described and exemplified in Section S3.

Interactions between particles potentially alter their settling behavior,^29–31^ which would impede measurements of the single particle settling velocity. To exclude conflicted measurements, an empirical model was employed for quality assurance and control. The model monitors particle–particle interactions according to the acquired image data and was proposed, calibrated and applied for this measuring setup before.^23,26^ Measured settling velocities were replaced by average values from valid data (see Section S9.2), if the model suggested a deviation of both more than 0.01 mm/s and 5% from the targeted single particle settling velocity. In total, 2480 settling experiments were conducted for this study, which amount to a cumulated runtime of 729 h.

### Analysis of Tire Marker Compounds

Both samples and all obtained fractions were analyzed by TED-GC/MS to identify and quantify tire marker compounds. TED-GC/MS is a multistage measurement technique combining extraction by thermal gravimetric analysis with the advantages of gas chromatography–mass spectrometry. A sample is heated under N_2_ atmosphere from 200 to 500 °C at a rate of 10 K/min. The decomposition gases are collected on a solid phase sorbent in the same temperature range and the loaded sorbent is automatically transferred to the GC/MS system. After release from the sorbent and separation on the GC column, the decomposition gases are detected in a mass spectrometer.

Car and truck tires partly consist of SBR and/or natural rubber (NR). These two compounds were used as polymer marker molecules indicating the presence of tire in the samples. SBR and NR were analyzed according to their specific decomposition products 2-phenylcyclohexene and limonene, respectively.^32,33^ It must be noted that limonene is not entirely specific to NR, but can also originate from plant material.^22,34^ Both polymers were quantified by one-point standard addition: After measuring a sample, a known mass of pristine SBR or NR was added and the measurement was repeated. Additional details can be found in Section S4 and in previous publications.^22,35^

### Estimation of Tire and TRWP Mass Contents

In the following, TRWP are conceptualized as binary mixtures of tire and nontire material. Both parts are supposed to exhibit homogeneous density, respectively. These simplifying assumptions are detailed in Section S5 together with derived descriptive equations. The estimated TRWP mass content (β_TRWP_) of a density fraction can be computed from its tire mass content (β_T_), the average fraction density (ρ) and the densities of the tire (ρ_T_) and nontire (ρ_NT_) materials that constitute TRWP:

$$\beta_{\mathrm{TRWP}}=\beta_{T}\frac{\rho (\rho_{\mathrm{NT}}-\rho_{T})}{\rho_{T}(\rho_{\mathrm{NT}}-\rho )}$$ 1

Hereafter, tire material density is always assumed as 1.2 g/cm^3^,^36^ while average nontire densities of 2.68 and 2.50 g/cm^3^ for the Road simulator and Tunnel sample, respectively, were deduced from pycnometer measurements (cf. Section S2). For the Tunnel sample and its associated fractions, the tire content is estimated from measured SBR: An average SBR content of tire tread of 11.3% is assumed, which was derived by Eisentraut et al.^22^ from a top-down balance of global production volumes. This conversion factor was deemed suitable, as the Tunnel sample is assumed to integrate over a vast diversity of tire products.

Yet, individual tire formulations can be highly variable.^32^ Therefore, the same approach is not suitable for the Road simulator sample, which contains material of three different tire products in unknown proportions and of inaccessible formulation.^24^ Instead, these model TRWP were assumed to be a binary mixture of abraded tire tread and added stone dust (cf. Section S2). In absence of contamination, all fractions of the Road simulator sample were supposed to consist entirely of TRWP–except the upper density fraction, which might contain pure stone dust as well. The fractions’ average densities (cf. Sections S1 and S2) were then vice versa used to estimate their tire contents via eq 1.

### Deriving TRWP Sizes and Settling Velocities

Particles measured during settling experiments cannot be assigned individually as TRWP or non-TRWP due to the lack of methods for in situ chemical identification. To deduce distributions of particle sizes and settling velocities for the TRWP contained in a sample, respective distributions measured in settling experiments for each density fraction of the sample are recombined based on the fractions’ tire and TRWP contents. The resulting distributions can be either weighted by TRWP-incorporated tire material or overall TRWP as well as by particle mass, volume, surface area, or count, respectively (see Section S6 for comprehensive computational details).

## Results and Discussion

### Tire Contents of Samples and Corresponding Fractions

The results of the different fractionation procedures applied to both samples are comprised in Figure 2—together with measured tire marker compounds as well as estimated tire and TRWP contents for each fraction (full underlying data is provided in Table S4). Figure 2a depicts the mass partitioning across the fractions: Regarding the Road simulator sample, the upper density fraction (>2.0 g/cm^3^) constitutes almost half of its mass (49.7%). The shares of the remaining fractions constantly decrease with decreasing densities, leaving only 2.5% of the sample mass within the lowest fraction (<1.6 g/cm^3^).

The Tunnel sample was first dry-sieved: 14.8% of its mass was found in the <20 μm size fraction, 53.1% in the 20–250 μm size fraction, and 32.1% in the largest size fraction >250 μm. The 20–250 μm size fraction was then fractionated with respect to density (cf. Figure 2a, right side): Here, the majority of its mass (55.7%) is again comprised in the upper fraction (>2.1 g/cm^3^). The other density fractions reveal at least a bimodal distribution with a local minimum at 1.8–1.9 g/cm^3^ (0.2%) and another peak in the 1.4–1.5 g/cm^3^ fraction. These fractionation results are in good agreement with a previous characterization of the Tunnel sample by Klöckner et al.,^21^ who identified similar density distribution patterns. The only notable difference is, that they found a higher mass share of the size fraction below 20 μm (31.7%, compared to 14.8% in this study) at the expense of the overall 20–250 μm fraction (39.3%, compared to 53.1% in this study). For size fractionation, Klöckner et al.^21^ applied wet sieving as opposed to dry sieving used for this study. Due to lower separation efficiency, dry sieving probably led to a higher share of particles smaller than 20 μm, that were still retained in the 20–250 μm fraction. However, this possible inconsistency is compensated, as the sizes of particles from the 20–250 μm fraction were analyzed individually down to 10 μm during the subsequent settling experiments.

Measured contents of SBR and NR mostly increase with decreasing density as is shown for both samples in Figure 2b. For each density fraction, estimations of TRWP and TRWP-incorporated tire contents are presented in Figure 2c. The upper density fraction of the Road simulator sample consists of only 6.3% tire material and might thus–depending on the assumed TRWP composition—contain up to 77.7% pure stone dust. The stone dust was added to the road simulator to promote tire abrasion, yet this does not necessarily mean that it is entirely incorporated in the produced model TRWP.

For the Tunnel sample, an overall SBR content of 9.8 mg/g was measured. This exceeds previously reported measurements for road runoff, road dust, and roadside soil (0.3–2.0 mg/g SBR),^37,38^ which presumably contain higher portions of non-TRWP particles, and is similar to the sediments of a pond treating highway runoff (9.6–10.9 mg/g SBR).^38^

The <20 μm size fraction of the Tunnel sample contains 15.9 mg/g SBR and 6.6 mg/g NR, while the size fraction >250 μm features only low contents of both tire polymers (0.6 mg/g SBR and 1.3 mg/g NR). The 20–250 μm size fraction was further fractionated by density, which allowed to estimate overall TRWP contents from SBR (Figure 2c). All estimated TRWP contents appear plausible except for a slight excess prediction of 108.7% ± 9.8 for the 1.4–1.5 g/cm^3^ fraction. All density fractions between 1.2 and 1.5 g/cm^3^ show estimated TRWP contents above 78.0%. This is in line with observations from optical microscopy: Mainly elongated, black particles were recorded for these fractions (cf. Figures 3b and S20–S22), as previously described as typical for TRWP.^18^ A similar visual impression arises for the lowest density fraction (<1.2 g/cm^3^): Although observing almost exclusively larger, TRWP-like particles (cf. Figures 3b and S19), the reduced SBR content leads to an estimated TRWP content of only 38.3 ± 3.4%. At the same time, remarkably high NR contents (>14 wt %) were measured for the two lowest density fractions, which might indicate the presence of TRWP originating from truck tires. Truck tires usually contain higher proportions of NR.^22,32^ Yet, NR measurements have to be interpreted with caution (see discussion in ‘Materials and methods’) and were therefore not additionally taken into account for estimating tire contents—even more so, in view of the low mass shares of the fractions concerned and the lack of an appropriate conversion factor.^22^

By combining mass shares (Figure 2a) and estimated tire contents (Figure 2c), the distributions of tire mass across all fractions were calculated for both samples, respectively, and are presented in Figure 3a. Notable enrichment patterns are revealed for the Tunnel sample (Figure 3a, right side): On the one hand, 54.0% of its tire mass is concentrated between 1.3 and 1.5 g/cm^3^ of the 20–250 μm size fraction, while the same fractions constitute only 8.7% of the total sample mass (cf. Figure 2a, right side). On the other hand, the upper size fraction (>250 μm) contains just 1.9% of the entire tire mass, yet accounts for 32.1% of the total sample mass. Exemplary microscope images (Figures 2d and S15–S29) support these observations.

The enrichment is less distinct for the Road simulator sample (Figure 3a, left side): The tire mass is distributed quite evenly across all density fractions, e.g., still 14.0% are found within the upper density fraction (>2.0 g/cm^3^). Additional fractionation above 2.0 g/cm^3^ might have refined these results. The average density of TRWP–here weighted by tire mass content–is remarkably higher for the Road simulator sample (∼1.82–1.87 g/cm^3^) than for the Tunnel sample (∼1.44 g/cm^3^ within 20–250 μm size fraction). Accordingly, increased mineral encrustations are visible from microscope images of the different density fractions of the Road simulator sample (cf. Figures S9–S14). Their homogeneous appearance further supports the assumption of the model TRWP as binary mixtures of abraded tire and stone dust.

The observed difference in average density between TRWP from both samples is in accordance with previous studies: Weyrauch et al.^39^ reported an average density of 1.8 g/cm^3^ for the Road simulator sample, while Klöckner et al.^21^ concluded that the TRWP from the Tunnel sample consist of approximately 75% tire material. This assumption would entail an average TRWP density of 1.4 g/cm^3^ (cf. eq S13).

### Settling Velocity Measurements of Fractionated Samples

In total, 123,572 particles were tracked during all settling experiments–characterizing their size and shape and measuring their terminal settling velocity. Prior to evaluation, the data set was reduced to 30,759 particles by excluding particles that did not meet respective cutoff criteria for particle size or settling velocity. These criteria were introduced to account for experiments with reduced runtime (cf. Table 1 and Section S3). Additionally, 38 particles (0.1%) were excluded as contaminants or agglomerates after visual classification by one human observer. The applied empirical model for particle interactions required a correction for 3.8% of the measured velocities (see Sections S9.1 and S9.2).

For each investigated density fraction of both samples, cumulative distributions of particle sizes and settling velocities were composed from the corresponding particle data (exemplified in Section S3). Size, surface area and volume estimates for an individual particle depend on its respective orientation toward the image plane, which can result in high uncertainty—especially for particles with large aspect ratios.^26,28^ However, this limitation of the employed imaging technique is compensated to a great extent by analyzing a large number of particles across the entire size range, which resulted in mostly smooth distributions and narrow confidence intervals. All distributions and corresponding metrics–derived with regard to either particle number, surface area or particle volume—are provided in Section S10. Full underlying data is openly available.^40^

Figure 4 summarizes particle size and settling velocity distributions related to particle volume, that were determined with for the different density fractions of both samples. They are simplified by histograms and boxplots.

Figure 4a reveals notable differences in particle sizes between density fractions: For the Road simulator sample, the median particle size clearly decreases with increasing density—from 89 and 90 μm for the two lowest density fractions to only 27 μm for the upper density fraction. As has been discussed before, these model TRWP can be conceptualized as a binary mixture of abraded tire and stone dust. Here, it is hypothesized that dust encrustations may not be evenly distributed across the entire volume of a particle, but concentrated near its surface to a certain degree. On average, preferential encrustation at the TRWP surfaces would result in larger model TRWP having a lower density due to a lower surface-to-volume ratio—and vice versa.

As mentioned before, the density fractions of the Tunnel sample discussed in the following were exclusively prepared from the 20–250 μm size fraction (cf. ‘Materials and Methods’). The lowest investigated density fraction (1.2–1.3 g/cm^3^) also shows an exceptionally high median particle size of 115 μm. These measurements are supported by optical microscopy (Figures S19 and S20)—indicating an even larger average particle size for the fraction <1.2 g/cm^3^, which could not be examined in settling experiments due to insufficient sample quantity. As already noted, the high NR content of both fractions also stands out. The observed increase in particle size, when compared to other fractions, might thus be associated to higher shares of TRWP originating from truck tires. Since truck-related TRWP could not be quantified specifically, this hypothesis cannot be further substantiated—yet it warrants future investigation. For the fractions between 1.3 and 1.7 g/cm^3^, which are assumed to contain the majority of the Tunnel sample’s tire mass (67.3%, cf. Figure 3a), a slight decrease of median particle size from 56 to 45 μm with increasing density is still visible. Yet, it is less pronounced than what was described for the model TRWP from the Road simulator.

The complex interplay regarding particle size and density, including partially inverse trends, is reflected in the measured settling velocities (Figure 4b). As expected, the maximum settling velocities were measured for the Tunnel sample’s two fractions with the highest density. While the highest median velocity of 1.23 mm/s also corresponds to the upper density fraction, it is immediately followed by 0.95 mm/s measured for the 1.2–1.3 g/cm^3^ fraction: Here, the lowest density of all investigated fractions is, with respect to settling velocity, compensated by the highest average particle size. Regarding the Road simulator sample, maximum measured settling velocity increases with density, too, from 4.32 to 10.34 mm/s. Yet, except for the lowest density fraction, the median velocity continuously decreases with increasing fraction density—from 1.27 to only 0.30 mm/s (also see Table S5).

### Deducing Sizes and Settling Velocities of TRWP

Settling experiments yielded distributions of particle sizes and settling velocities for the different density fractions of both samples. For each sample, these measurements can be recombined based on the fractions’ estimated tire and TRWP contents (detailed in Section S6). Due to the successful enrichment of TRWP in specific fractions, meaningful distributions of sizes and settling velocities can be derived for the TRWP from both samples. Figure 5 shows the results related to TRWP-incorporated tire mass—further results, e.g., related to particle volume, surface area and count as well as to overall TRWP mass are provided in Section S11.

The depicted 95% confidence intervals (CIs) emphasize that the associated uncertainty could be effectively narrowed down: ‘Mean data CI’ is constructed from the CIs measured for every fraction, hereby assuming TRWP as representative particles of their respective density fractions. The ‘edge case CI’ additionally assumes that all TRWP within a fraction are either the smallest/largest or slowest/fastest settling particles, respectively. Thus, it provides a very conservative, uttermost estimate of the associated uncertainty. Both intervals were additionally extended via resampling the mean fraction densities and SBR contents (further details given in Section S6.3). The noninvestigated size fractions of the Tunnel sample were assigned settling velocities below (for <20 μm fraction) or above (for >250 μm fraction) the range measured for the 20–250 μm fraction (cf. shades in Figure 5), which is a reasonable assumption despite possible variations in density. Regarding the Road simulator sample, the lower end of both distributions might be slightly trimmed due to the minimum ECD of 10 μm for particle detection (see ‘Materials and Methods’).

Table 2 summarizes the quartiles of particle size and settling velocity distributions weighted by TRWP-incorporated tire mass, as depicted in Figure 5, and weighted by overall TRWP mass, respectively. TRWP mass contents of fractions are computed using eq 1. TRWP densities are assumed to be mean fraction densities, respectively, except for the terminal fractions (see Section S6.4 for further details).

**Table 2:** Quartiles of Particle Size and Settling Velocity Distributions Derived for TRWP from the Road Simulator and Tunnel Sample^a^

		particle size (μm)		settling velocity (mm/s)	
distributions weighted by···	quartiles	road sim. sample	tunnel sample	road sim. sample	tunnel sample
tire mass (cf. Figure 5)	**Q1:25%**	30	22	0.25	0.06
	**Q2: median**	54	44	0.65	0.22
	**Q3:75%**	89	84	1.41	0.70
**TRWP mass**	**Q1:25%**	26	35^b^	0.21	0.15^b^
	**Q2: median**	45	54^b^	0.54	0.35^b^
	**Q3:75%**	78	95^b^	1.25	0.93^b^

a Distributions are weighted by TRWP-incorporated tire mass and TRWP mass, respectively.

b Values exclusively refer to 20–250 μm size fraction.

With respect to the Road simulator sample, it is apparent that the quartiles weighted by TRWP-incorporated tire mass exceed those weighted by full TRWP mass. A corresponding increase of particle size with decreasing fraction density, which in turn translates to increasing tire content of respective TRWP, was noted before (cf. Figure 4). It was potentially linked to a preferentially superficial encrustation of the abraded tire rubber with stone dust. For the Tunnel sample, a similar comparison cannot be made, since the metrics and distributions weighted by TRWP mass were exclusively derived for the 20–250 μm size fraction.

The contribution of the remaining size fractions to overall TRWP mass could vary widely with respect to the assumed mean TRWP densities: between 14 and 54% for the <20 μm size fraction and between 1 and 5% for the >250 μm size fractions for the peripheral assumptions of 1.2 or 2.2 g/cm^3^, respectively. It is probably more realistic to assume mean densities such as 1.7 g/cm^3^ for TRWP below 20 μm and 1.2 g/cm^3^ for TRWP above 250 μm in size, which would result in the investigated 20–250 μm size fraction constituting 71% of the overall TRWP mass. Still, reliable estimates cannot be made without additional data. In the following, Road simulator and Tunnel sample are therefore compared and discussed on the basis of metrics weighted according to TRWP-incorporated tire mass.

### Sample Comparison and Representativeness

Despite a rather confined emission scenario, TRWP are heterogeneous particles with variable properties. Thus, the conceivable range of the densities and sizes of environmental TRWP can be large, but is only characterized in part so far and based on few samples.^18,24,41,42^

In this study, the TRWP size was found to be similar for both investigated samples—with median values of 54 μm for the Road simulator and 44 μm for the Tunnel sample as weighted with respect to incorporated tire mass. For the Road simulator sample, the observed size is in line with previous studies.^24,39^ For the Tunnel sample, Kovochich et al.^43^ reported a larger average particle size of 94 μm (volume-related) based on a smaller set of TRWP identified via scanning electron microscopy analysis of the dry sample. In contrast, the contents of organic tire constituents as well as tire-related Zn measured by Klöckner et al.^21^ in different size fractions of the Tunnel sample suggest a median TRWP size within the fraction between 20 and 50 μm, which would support this study’s results.

The median settling velocities–0.65 mm/s for the Road simulator and 0.22 mm/s for the Tunnel sample, respectively—deviate more significantly (factor 3.0) than would be explained by the difference in particle size alone (factor 1.5 according to Stokes’ law). Yet, this deviation is highly consistent, if the difference in average densities between both samples is included as well (factor 2.9–3.1 according to Stokes’ law).

Weighted by incorporated tire mass, an average density of ∼1.82–1.87 g/cm^3^ for model TRWP from the Road simulator sample compares to only ∼1.44 g/cm^3^ for TRWP from the Tunnel sample’s 20–250 μm size fraction. It can be argued that the samples thus cover a broad range of realistic TRWP densities. Klöckner et al.^21^ already noted, that the average densities of the same investigated Tunnel sample TRWP were rather low—e.g., when compared to samples from a sedimentation basin and a soil retention filter^44^ with TRWP densities primarily being between 1.5 and 1.9 g/cm^3^.

Jung and Choi^45^ investigated three different road dust samples and found the majority of TRWP to have lower densities between 1.2 and 1.7 g/cm^3^, too. Moreover, they pointed out a similar inverse trend between TRWP size and density as was partly found in this study. Murakami et al.^46^ also observed increased polycyclic aromatic hydrocarbon contents in the density fraction below 1.7 g/cm^3^ for different road dust samples, which they partially attributed to TRWP. In general, current characterizations of TRWP are based on surprisingly little empirical data^42^ and are often exclusively rooted in theoretical considerations.^47^ The latter can also be ambiguous: The often cited^48–50^ average TRWP density of 1.8 g/cm^3^ derived by Kreider et al.^47^ only holds true for a nontire content (2.4 g/cm^3^ assumed here by the authors) of 50% based on volume—with respect to 50% mass content it would reduce to 1.6 g/cm^3^ (see Section S5).

In summary, this study’s characterization of both the Tunnel and Road simulator sample is consistent with the existing literature. In direct comparison, the Tunnel sample is more relevant and representative as it contains a broad range of TRWP generated under real environmental conditions.

### Empirical Input Data for TRWP Transport Modeling

The terminal settling velocity of TRWP was identified as the most decisive parameter for modeling TRWP transport and fate in the aquatic environment.^15^ So far, existing models had to estimate velocities due to a lack of empirical data: Unice et al.^16^ estimated a settling velocity of 3.05 mm/s for TRWP according to their central assumptions of 1.8 g/cm^3^ density, 105 μm particle size, and an aspect ratio of 0.64. Bondelind et al.^17^ estimated settling velocities of 2.40 mm/s (1.9 g/cm^3^, 75 μm) and 0.13 mm/s (1.7 g/cm^3^, 20 μm), respectively. Such estimates are likely error-prone due to the high variability of TRWP densities and sizes. In fact, two of the three aforementioned estimates by far exceed the median TRWP settling velocities measured in this study, i.e. 0.65 mm/s for the Road simulator sample and 0.22 mm/s for the Tunnel sample.

Significant overestimations of TRWP settling velocities can lead to a substantial underestimation of the long-range transport of TRWP in models. For the Seine catchment, Unice et al.^16^ concluded that only 2% of all generated TRWP reach the estuary. Yet, their model’s TRWP settling velocity estimate exceeds this study’s median measurements by 5 and 14 times, respectively. Riverine TRWP inputs into the oceans could thus be considerably higher than assumed so far, which would better explain recent measurements of TRWP in marine environments.^51,52^

Consequently, the detailed empirical data on TRWP settling velocities and particle sizes from this study is valuable for refining TRWP transport models. The data should also be considered for dimensioning TRWP retention measures in areas of concentrated traffic, e.g., retention ponds and infiltrations basins.^53–56^

Apart from deriving specific average values, the experimentally obtained probability distributions of particle size and settling velocity (as presented in Figure 5) might also serve as direct inputs for probabilistic transport modeling. Here, sufficient numbers of randomized virtual samples of settling velocity and particle size are drawn from the respective probability distributions in order to describe the population of TRWP particles in a whole. In case any model additionally requires TRWP density as input, the density can be virtually resampled according to the fractionation results (e.g., Figure 3a), before virtually resampling size and settling velocity from the distributions measured for this density fraction (cf. Section S10). Compared to deterministic models, probabilistic modeling could be advantageous with respect to the inhomogeneous particle properties of TRWP.

In general, distributions and average values for particle sizes and settling velocities can be deduced with respect to particle mass, volume, surface area or number—each related to either overall TRWP or TRWP-incorporated tire material.

When implementing a transport model, the appropriate choice of reference should be made in accordance with the respective modeling goal. For example, the tire mass incorporated in TRWP (as shown in Figure 5) likely correlates with the total amount of leachable tire constituents. For modeling general mass budgets, overall TRWP mass should be chosen as reference instead. Particle number or surface area might be more relevant, when focusing on particle-related ecotoxicological end points or leaching kinetics, respectively.

This study’s data on settling velocities and particles sizes of TRWP from the Road simulator and Tunnel sample has been processed for each possible set of references to serve as potential model input (provided in Section S11). Since all settling velocities were measured in deionized water at 15 °C, they have to be corrected for other temperatures and salinities. Corrections must be made separately for each density fraction before merging the data (as described in Section S11). A respective script (Python) for computing such corrected settling velocity distributions and all required input data are made freely available.^40^

### Future Research Perspective

This study demonstrates the successful TRWP enrichment via fractionation followed by an effective combination of integral measurements of tire marker compounds with representative particle data gathered for each fraction. Comparable approaches could become a viable alternative to the direct identification of single TRWP from samples,^24,43^ since they potentially extent the range of examinable features as was shown here for settling velocities. Comprehensive empirical data focusing on probability distributions of critical particle properties^57^ are especially important for TRWP due to its complex composition.

In this study, estimations of tire content were based on measured SBR. To increase robustness, other promising tire constituents might be evaluated as additional markers in the future.^58^ Moreover, only two different samples—environmental TRWP as well as potential model TRWP—could be investigated and compared within the scope of this study. This marks significant progress, since any similar empirical data on TRWP was lacking so far, and both samples cover a wide range of plausible TRWP densities. Nonetheless, the variability of TRWP requires continued investigation of appropriate samples in order to further explore potential influencing factors like vehicle diversity, tire composition,^32,59^ driving and braking behavior, different road surfaces^59^ and weather conditions. For example, significantly larger TRWP sizes were recorded for samples from river sediments.^43,48^ However, sampling TRWP farther away from their source possibly causes shifts in density and particle size due to selective transport and deposition mechanisms. The potential selectivity of transport processes is emphasized by the large range of settling velocities measured for TRWP in this study.

Overall, many uncertainties and open questions still surround the environmental mobility, fate and impact of TRWP—especially due to variable particle properties and significant spatial and temporal heterogeneity of key driving factors (e.g., precipitation^7,60^ ). To address knowledge gaps, future research efforts should include various perspectives and disciplines: Besides additional settling data, lab- or mesocosm-scale experiments are needed to simulate and better quantify specific transport processes, e.g., fluvial transport^14,61^ or artificial runoff scenarios^62^ to elucidate the remobilization potential of TRWP from terrestrial reservoirs such as road surfaces and nearby soils.^3,63^ A careful selection of appropriate model particles that closely resemble environmental TRWP in relevant particle properties is crucial for such experiments. Gathered empirical data should be constantly integrated into transport models, which could on one hand be validated according to continued environmental sampling of TRWP, yet on the other hand could also be used to inform more targeted sampling campaigns. Findings from these different research endeavors could then be integrated into toxicological experiments and assessments, e.g., by identifying and prioritizing potential TRWP “hot spots” and the associated species and habitats that may be affected.

This study’s results emphasize the importance of accounting for the variability of TRWP properties in modeling, but also in the choice of representative model particles as an essential tool for continued mechanistic research on TRWP. The detailed empirical data that is presented offers starting points and paths to achieve these goals.

## Supplementary Material



## Data Availability

Single particle raw data of all experiments, measured cumulative particle size and settling velocity distributions for all investigated density fractions, cumulative distributions with respect to either TRWP-incorporated tire material or TRWP composed from full settling data for both samples, respectively, as well as a Python script to correct these distributions for water temperatures and salinities other than the experimental conditions are provided in the Zenodo repository “Additional data for *Settling velocities of tire and road wear particles: Analyzing finely graded density fractions of samples from a road simulator and a highway tunnel*” at 10.5281/zenodo.15088430 (DOI).

## Notes

Methodical details on density fractionation, pycnometer measurements, deriving size and velocity distributions from settling data, TED-GC/MS measurements, estimating the nontire fraction of TRWP as well as on merging size or velocity distributions measured for different density fractions; and tabular results of fractionation procedures, optical microscope images of all considered samples and fractions, results of data quality assurance measures applied to the gathered settling data, measured particle size and settling velocity distributions for all investigated density fractions as well as various cumulative distributions composed from full settling data with respect to either TRWP-associated tire material or TRWP (PDF)
