# Supplementary material for: Settling Velocities of Tire and Road Wear Particles: Analyzing Finely Graded Density Fractions of Samples from a Road Simulator and a Highway Tunnel
Source: Environ Sci Technol. 2025 Jun 18;59(26):13434–46. doi: 10.1021/acs.est.5c04165 (PMC12243122; doi:10.1021/acs.est.5c04165)
Supplement: Supplementary file 1 [file es5c04165_si_001.pdf]

SUPPORTING INFORMATION

Settling velocities of tire and road wear particles: Analyzing finely graded density fractions of samples from a road simulator and a highway tunnel

Stefan Dittmar,[*,†,‡] *Steffen Weyrauch,*[§] *Thorsten Reemtsma,*[*,§] *Paul Eisentraut,*[||,#] *Korinna Altmann,*[||] *Aki S. Ruhl,*[†,#] *Martin Jekel*[†]

[†]Chair of Water Quality Control, Technische Universität Berlin, Sekr. KF4, Straße des 17. Juni 135, 10623 Berlin, Germany

[‡]GEOMAR Helmholtz Centre for Ocean Research Kiel, Wischhofstraße 1–3, 24148 Kiel, Germany

[§]Helmholtz-Centre for Environmental Research – UFZ, Department Analytical Chemistry, Permoserstrasse 15, 04318, Leipzig, Germany

[||]Bundesanstalt für Materialforschung und -prüfung (BAM), Unter den Eichen 87, 12205, Berlin, Germany

[#]German Environment Agency (UBA), Section II 3.3, Schichauweg 58, 12307 Berlin, Germany

* Corresponding authors: stefan.dittmar@tu-berlin.de, thorsten.reemtsma@ufz.de

Number of pages: 68
Number of figures: 68
Number of tables: 9

Content

S1 Density fractionation 3

S2 Pycnometer measurements 4

S3 Deriving size and velocity distributions from settling data 5

S4 Further details on TED-GC/MS measurements 9

S5 Estimating the non-tire fraction of TRWP from mixed density 10

S6 Merging size or velocity distributions of different density fractions 12

S6.1 Background and definitions 12

S6.2 Derivation of weighting factors ωi for different target distributions 12

S6.3 Uncertainty and confidence intervals 13

S6.4 Implementation and assumptions 14

S7 Tabular results of fractionation procedures 19

S8 Optical microscope images 20

S8.1 Road simulator sample 20

S8.2 Tunnel sample 22

S9 Data quality of settling velocity measurements 27

S9.1 Visual inspection of particles and contaminations 27

S9.2 Applying empirical model for particle-particle interactions 28

S10 Measured particle size and settling velocity distributions 43

S11 Composed particle size and settling velocity distributions 59

S12 Particle aspect ratios obtained from settling data 66

S13 References 67

S1 Density fractionation

In the following, further details on the applied density fractionation method are provided. To execute fractionation, the particle sample was dispersed by ultrasonication and shaking in a heavy liquid sodium polytungstate (SPT) solution (Labsolute, Th. Geyer, Germany) with the highest targeted density, e.g. 2.1 g/cm^3 for the Tunnel sample, and centrifuged at 1970 g for 10 min (Rotanta 460, Hettich, Germany). The settled fraction, here > 2.1 g/cm^3, was trapped in the tip of the tube by shock-freezing with liquid nitrogen. The floating fraction, here < 2.1 g/cm^3, was transferred into another tube where the SPT solution was diluted to the subsequent lighter density by the addition of ultra-pure water and then dispersed and centrifuged again. These steps were repeated until the lowest targeted density was reached. Fractionated particles were always recovered from suspension via vacuum-filtration onto a 0.8 µm nitrocellulose membrane filter (LabSolute, Th. Geyer, Germany), then washed with ultrapure water (Milli-Q integral, Merck, Germany) and dried at 50°C. Usually, a fractionation step was carried out via processing several centrifugation tubes in parallel, which allows to analyze standard deviations (cf. Table S4). This was not feasible for fractionation of the Tunnel sample below 1.4 g/cm^3 – due to small remaining sample masses, they were combined in one centrifuge tube.

Table S1 comprises the targeted and the actually measured density limits for all generated fractions of both the Road simulator sample and the Tunnel sample. Throughout the main publication and the Supporting Information, targeted density limits are used for denominating samples in order to ensure readability and avoid unnecessary complexity, yet the measured values, respective averages or bootstraps are used whenever fraction densities are inputs of a calculation (e.g. see section S6).

Table S1. Targeted and measured density limits for all density fractions of both samples.

Sample/Fraction name			Lower density limit, ρ_{lower} (g/cm^3)		Upper density limit, ρ_{upper} (g/cm^3)	
			Targeted	Measured	Targeted	Measured
Road simulator sample	Density fractions (g/cm^3)	< 1.6	-	-	1.60	1.60*
		1.6–1.7	1.60	1.60*	1.70	1.70*
		1.7–1.8	1.70	1.70*	1.80	1.80*
		1.8–1.9	1.80	1.80*	1.90	1.90*
		1.9–2.0	1.90	1.90*	2.00	2.00*
		> 2.0	2.00	2.00*	-	-
Tunnel sample	Density fractions (g/cm^3)	< 1.2	-	-	1.20	1.20
		1.2–1.3	1.20	1.20	1.30	1.29
		1.3–1.4	1.30	1.29	1.40	1.40
		1.4–1.5	1.40	1.40	1.50	1.50
		1.5–1.6	1.50	1.50	1.60	1.61
		1.6–1.7	1.60	1.61	1.70	1.74
		1.7–1.8	1.70	1.74	1.80	1.82
		1.8–1.9	1.80	1.82	1.90	1.88
		1.9–2.0	1.90	1.88	2.00	1.99
		2.0–2.1	2.00	1.99	2.10	2.14
		> 2.1	2.10	2.14	-	-

*measured density within ±0.01 g/cm^3 from targeted density

S2 Pycnometer measurements

The mean densities of the upper density fractions of both samples as well as of the size fractions of the Tunnel sample were determined at ~20 °C via pycnometer measurements with isopropanol. The results are comprised in Table S2.

Table S2. Pycnometer-measured densities of selected fractions. For Tunnel sample fractions, mean densities of non-tire material computed from estimated tire content are included.

Sample/Fraction name		Measured density (g/cm^3)	Replicates	Tire content (wt%)	Non-tire density (g/cm^3)
Road simulator sample	> 2.0 g/cm^3	2.488 (2.467, 2.508)	2	6.38*	2.684‡
Tunnel sample	< 20 µm	2.172 ± 0.019	3	14.04†	2.503§
	20–250 µm	2.105 (2.092, 2.118)	2	14.08†	2.402§
	> 2.1 g/cm^3	2.490 ± 0.033	3	0.47†	2.503§
	> 250 µm	2.488 ± 0.111	3	0.58†	2.504§

* computed from measured non-tire density and assumed tire density of 1.2 g/cm^3 (via Equation S13, left side)

† estimated from SBR measurements, e.g. see Table S4 and main publication

‡ measured density of added stone dust

§ computed from measured density, tire content and assumed tire density of 1.2 g/cm^3 (via Equation S13, left side)

For the Tunnel sample fractions, non-tire densities were computed from the measured mean densities and estimated tire contents (also see section S5). The results coincide except for the size fraction 20–250 µm: The lower value could potentially be explained by an increased proportion of truck tire TRWP contained within this fraction, which might not be fully covered by estimating the tire content solely based on average SBR content (styrene-butadiene rubber). Please note the related discussion in the section ‘Tire content of samples and corresponding fractions’ of the main publication. Consequently, the non-tire density computed for the 20–250 µm fraction is not considered and the non-tire density of the Tunnel sample is assumed as average of the remaining values (2.503 g/cm^3). It is used for calculating the non-tire fractions of TRWP (see section S5).

With respect to the Road simulator sample, 15 g of stone dust were added to the simulator every 1.5 h during particle generation[1]. Therefore, the non-tire fraction can be considered to consist mainly of stone dust with only minor contribution of wear of the test pavement itself. In fact, the stone dust was added to prevent the agglutination of the pavement with tire material and thus ensure TRWP generation in the first place. Hence, the density of the stone dust was determined via pycnometer measurement as well (2.684 ± 0.030 g/cm^3) and is generally assumed as the non-tire density for TRWP of the Road simulator sample.

The lower density fractions of both samples could not be measured at all due to insufficient sample mass. Instead, mean densities of 1.55 and 1.2 were assumed throughout for the < 1.6 g/cm^3 fraction of the Road simulator sample and the < 1.2 g/cm^3 fraction of the Tunnel sample, respectively.

S3 Deriving size and velocity distributions from settling data

In this study, particle size is characterized as the equivalent diameter $d_{eq,Bagheri}$, which was proposed by Bagheri et al.[2] and is computed as:

$$d_{eq,Bagheri} = \frac{\overline{d_{2D}}}{1.022 \cdot \psi^{-0.29}} \cong \frac{\overline{d_{2D}}}{1.022 \cdot {f_{circ}^{*}}^{-0.29}} \qquad \text{(S1)}$$

The sphericity ψ is hereby estimated as the circularity f_{circ}^{*} and the mean diameter $\overline{d_{2D}}$ is computed as the mean equivalent circular diameter (ECD) of the three particle contours with the largest, smallest and intermediate contour area (further details were already provided in section S3 of the Supporting Information of a previous publication[3]). As a second size descriptor, the overall average ECD of a particle is considered in the following, since it is commonly used in many publications[4,5], although it possibly overestimates particle volume compared to the corrected $d_{eq,Bagheri}$.[2]

In general, cumulative distributions related to particle number, surface area and volume were derived, respectively (see results in section S10). Surface area (SA) and volume (V) were estimated individually for each particle based on respective corrections proposed by Bagheri et al.[2]:

$$V = \frac{\pi \cdot {d_{eq,Bagheri}}^{3}}{6} \qquad \text{(S2)}$$

$$SA = \frac{\pi \cdot {d_{eq,Bagheri}}^{2}}{\psi} \cong \frac{\pi \cdot {d_{eq,Bagheri}}^{2}}{f_{circ}^{*}} \qquad \text{(S3)}$$

Settling experiments were conducted in three of four sets of experimental runs, in order to investigate density fractions of the Road simulator sample or Tunnel sample, respectively. With each set, the runtime was decreased, while the number of experimental runs was increased – and usually the applied particle dose per run as well (cf. Table 1 of the main manuscript). This was done in order to characterize a particle population more precisely by sampling a sufficient number of larger particles, which are usually less frequent. The differences in particle dose, numbers of runs and runtime, in turn, have to be considered when merging settling data from all sets of experiments in order to obtain distributions of settling velocity and particle size.

Figure S1 exemplifies the derivation of a volume-specific distribution of the particle size (d_{eq}) for the 1.8–1.9 g/cm³ density fraction of the Road simulator sample. 32 mg of sample (80x 0.4 mg) were nominally dosed during the experiments of Set 3 – compared to only 2 mg for Set 1 (20x 0.1 mg). Yet, the particle population observed in experiments of Set 3 is partially truncated, because not all slower settling particles pass the field of view (FOV) within the shortened runtime (4 min 15 s for Set 3 compared to 90 min for Set 1). Therefore, detected particles below set-specific size cutoffs (46 µm for Set 2 and 80 µm for Set 3, cf. Figure S1b) were not considered. To finally obtain the targeted distribution, the estimated volumes of particles from previous sets detected below cutoffs are now corrected with a factor compensating for the lower investigated sample mass. Here, this is a factor of 21 (0–46 µm) or 4.2 (46–80 µm), respectively. This correction results in the cumulative distribution shown in Figure S1c and can be similarly executed, if particle numbers or surface areas are considered instead of estimated volumes.

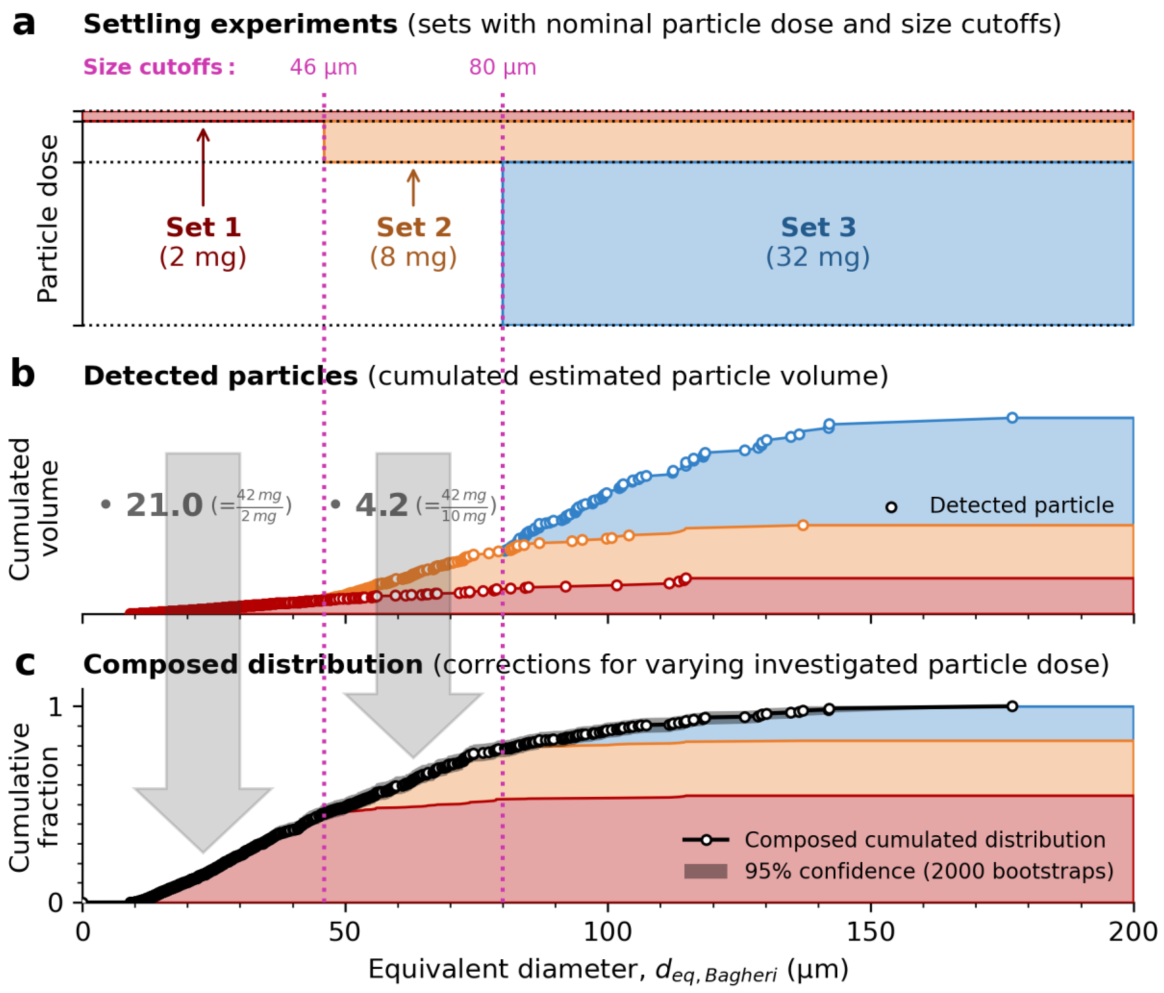

Figure S1. Derivation of the volume-specific cumulative distribution of particle size from settling data exemplified for the 1.8–1.9 g/cm³ fraction of the Road simulator sample. Includes particle doses for all sets of experiments (a), size of detected particles (b) and composed distribution (c).

Set-specific cutoffs are always determined automatically for composing a size or velocity distribution. They are set to zero for Set 1, as the respective runtime was fixed based on preliminary experiments, hence ensuring to cover the considered particle population completely – with respect to the minimum ECD of 10 µm for particle detection. For subsequent sets, cutoffs are computed according to the data from previous sets: For both particle size or velocity, cutoffs are set to the lowest value above which less than 5% or at most one particle were not detected within the now considered runtime, respectively (cutoff tolerance $\alpha = 5\%$).

Figure S2 shows volume-specific cumulative settling velocity distributions for the 1.8–1.9 g/cm³ fraction of the Road simulator sample, which were derived for different cutoff tolerances between 0% and 50%. Considering this example, the effect on the distribution appears to be insignificant between cutoff tolerances of 0 and 20%, and only shows at a very high cutoff tolerance of 50%. This is probably due to the fact, that settling experiments of a set were usually conducted in immediate succession. Therefore, a certain proportion of slow settling particles, that would usually be truncated due to the decreased runtime, was still captured in subsequent experiments. Overall, 5% appears to be a reasonable choice for the cutoff tolerance.

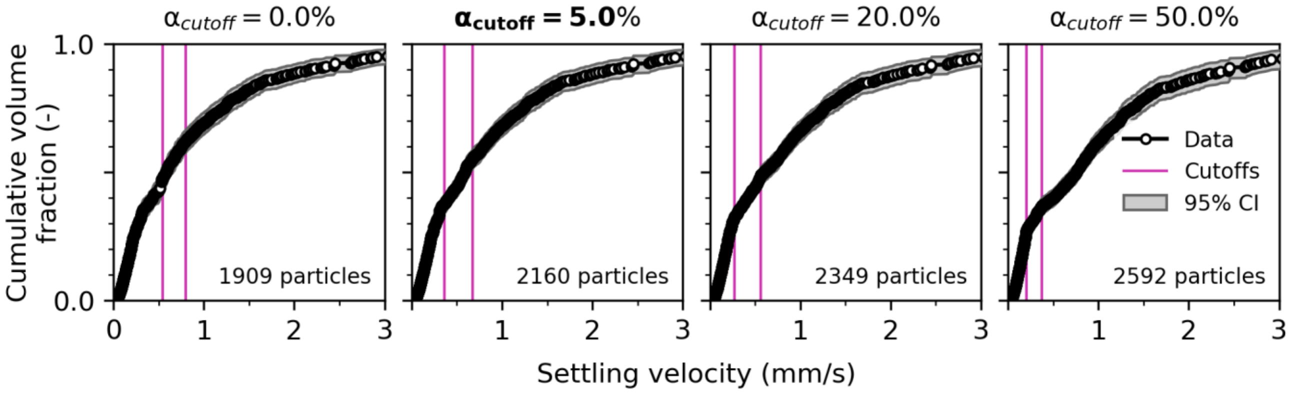

Figure S2. Cumulative, volume-specific settling velocity distributions for density fraction 1.8–1.9 g/cm³ of the Road simulator sample derived for different cutoff tolerances α.

To assess the uncertainty within a derived distribution, a 95% confidence interval was derived via bootstrapping: Each distribution was resampled 2000 times with replacement. Figure S3 exemplarily shows different cumulative distributions of particle size for the 1.8–1.9 g/cm³ density fraction of the Road simulator sample. They were derived from the measurement results of Set 1, of Set 1 and Set 2 as well as of all sets, respectively. The success of the experimental strategy is evident, when comparing the different confidence intervals: The integration of additional measurement data – with focus on larger particles – clearly refines the distribution and thus significantly decreases the associated uncertainty.

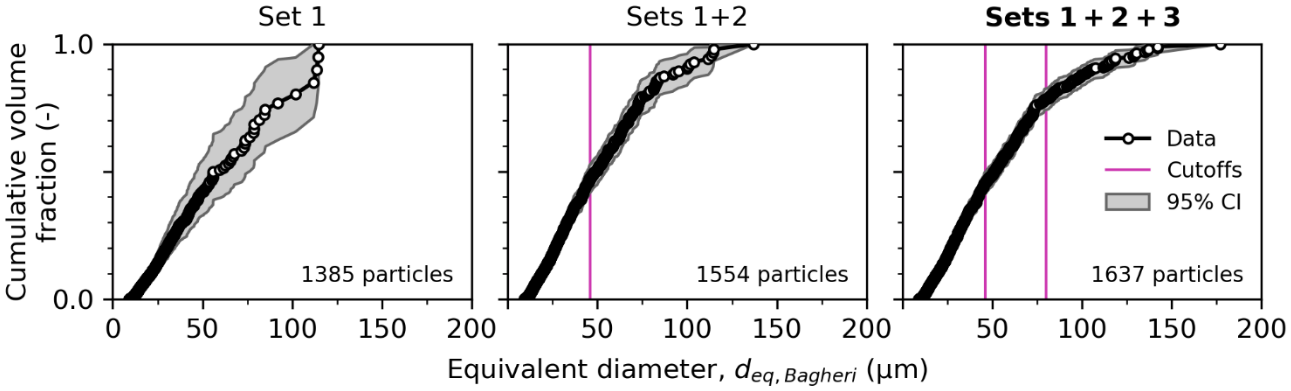

Figure S3. Cumulative, volume-specific particle size distributions for density fraction 1.8–1.9 g/cm³ of the Road simulator sample. The distributions are based on data from different combinations of sets of experiments.

Finally, an individual correction factor f_{corr} was applied for each particle to account for differences in detection probability, which are caused by the restricted field of view (FOV). With increasing particle width, the probability of capturing a particle decreases. f_{corr} is therefore computed as the ratio of the FOV width W and an effective width W_{corr}:

$$f_{corr} = \frac{W}{W_{corr}} = \frac{W}{W - W_P - W_T} \tag{S4}$$

W_{corr} is computed individually for each particle, subtracting the width of the particle contour (W_P) as well as a possible additional width due to the trajectories inclination (W_T).

W_T is approximated from the trajectory's width Δx and height Δy, both derived from particle centroids, as well as the FOV height H. Moreover, the particle height H_P is included, since passing at least 50% of the distance $H - H_P$ is mandatory for further consideration of a particle (cf. Dittmar et al.[6]):

$$W_T = \frac{\Delta x}{\Delta y}(H - H_P) \cdot 50\% \cdot \frac{H - H_P}{H} = \frac{\Delta x \cdot (H - H_P)^2}{2 \cdot \Delta y \cdot H} \tag{S5}$$

Figure S4 illustrates the computation of f_{corr} for an exemplary particle and comprises all required quantities. For the given example, the correction factor takes the value of 1.142. Overall, computed correction factors range between 1.003 and 1.881.

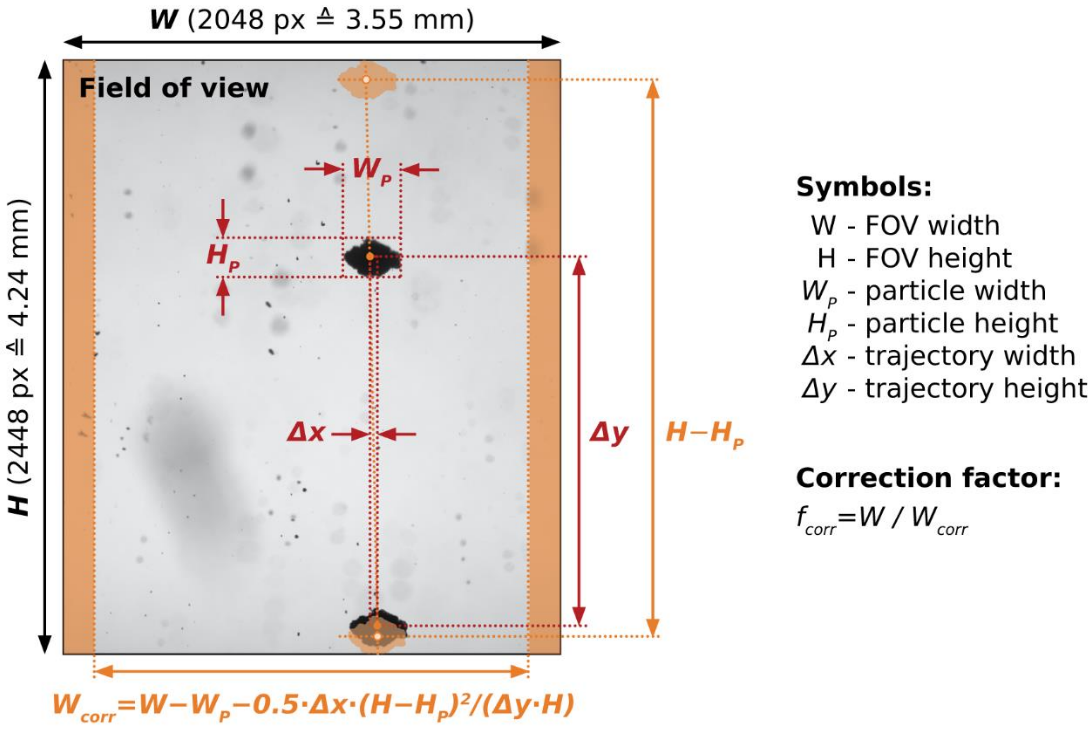

Figure S4. Exemplary computation of the correction factor f_{corr} for balancing different detection probabilities on the level of individual particles.

Beside the extent of the FOV, the depth of field (DOF) could inflict size dependent differences in detection probability, e.g. if the focal range and thus the observed water volume would be increased for larger particles. The employed image processing algorithm automatically assesses whether a particle is in focus. The calibration of the detection parameters actually revealed a dependency between the associated focal range and particle size for lower detection accuracies (e.g. ± 5 µm, cf. Figure S10 of the Supporting Information of Dittmar et al.[6]). Yet, this dependency is insignificant for the chosen detection accuracy of ± 1 µm and thus no additional correction factor was applied.

For each composed cumulative distribution, the best-fitting continuous distribution function from the Python package *scipy.stats*[7] was identified. For the fitting procedure, a probability distribution function was derived by resampling 5000 cumulative fractions from the measured cumulative distribution – with linear interpolation between data points. Fitting results are included in section S10. The respective fitted parameters are explained in the package documentation[8].

S4 Further details on TED-GC/MS measurements

In preparation for the TED-GC/MS measurements, TGA measurements were carried out on a TGA/DSC3+ system (Mettler-Toledo, Germany) to estimate the elastomer and pyrolysable matter content in order to avoid overloading and memory effects on the more sensitive instrument. All TED-GC/MS measurements included 4 µg of the Internal Standard $^{13}C_6$-labelled polystyrene. Samples were heated in aluminum oxide crucibles between 200 and 500°C (10 K/min, TGA2, Mettler-Toledo) under nitrogen atmosphere (30 mL/min). The decomposition products were transferred through a heated coupling device (240°C, Gerstel, Germany and BAM, Germany) and collected on polydimethylsiloxane sorption bars (40°C, SorbStar, ENVEA, Germany). The loaded sorption bars were moved (MPS2, Gerstel) to a thermal desorption unit (TDU, 50-200°C, helium, 40 K/min, 5 min isotherm, Gerstel) where the decomposition products were remobilized. After cryo-focusing and rapid injection (solvent vent mode) in a cooled injection system (CIS, -100°C to 270°C, 12 K/s, Gerstel), the decomposition products were separated in a GC (7890B, Agilent, USA) equipped with a HP-5ms column (40-260°C, 5 K/min, 260-300°C, 10 K/min, 1 mL/min He, Agilent) and detected with a mass spectrometer (MSD 5977B, EI, 70 eV, scan mode 35-350 m/z, Agilent).

One-Point-Standard-Addition was chosen as quantification method. An aliquot of each sample was measured additionally to another aliquot spiked with pristine SBR and NR. The difference in the peak areas of the decomposition products of SBR, 2-Phenylcyclohexene, and of NR, Limonene, was used to calculate the mass content of the respective polymers in the original sample. However, Limonene can have other sources than NR such as poly- or oligoisoprenes in plant matter. The results for NR have a distinct degree of uncertainty associated with them due to the possible presence of plant material in the Tunnel sample.

In general, some road surfaces contain SBR as binder, e.g. from recycled tire material. If measured, this SBR could be misinterpreted as SBR from abraded tire tread (incorporated in TRWP). Yet, both road surfaces sampled for this study did not contain additional SBR: The respective highway tunnel has a concrete roadway, while the road simulator is lined with the tarmac AC 11 D S 25/55-55 (Südwest-Asphalt, Germany), which does not contain recycled tire material or any elastomers as was confirmed by the manufacturer. In the context of this study, SBR can thus be regarded as a marker molecule exclusively for the tire material associated with TRWP.

Typically, results for SBR and NR measured with the TED-GC/MS vary with a relative standard deviation of 9 and 15%, respectively. These values might be exceeded in the present study due to partially low amounts of sample intake increasing the relevance of sample inhomogeneities. The reduction of sample mass used for analysis was necessary because of unusual high polymer contents. However, double measurements of a selected sample exhibited high reproducibility (8.85% relative standard deviation for SBR and 9.05% for NR).

To avoid contamination, all samples were stored in plastic/rubber-free containers and handled with plastic/rubber-free tools. Exposition to the laboratory’s air was minimized. Analytical blank measurements were collected before each sample run. Peaks originating from the Internal Standard were checked for plausibility. Results calculated through chromatographic data were compared to data from the TGA-runs as a further plausibility test.

S5 Estimating the non-tire fraction of TRWP from mixed density

TRWP can be considered a mixture of tire material and encrustations[9,10], e.g. minerals from road dust. In the following, we conceptualize both as homogeneous fractions as is illustrated in Figure S5 – complemented with respective symbols and indices, which will be used throughout this section.

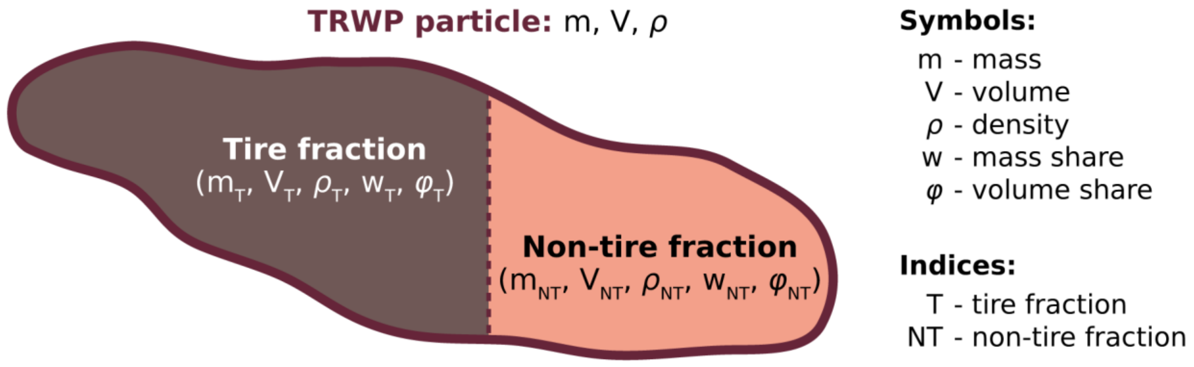

Figure S5. Simplified concept of single TRWP consisting of a homogeneous tire and non-tire fraction, respectively. Corresponding symbols and indices are indicated.

Based on this simplification, we can now set up a simple mass balance for a single particle:

$$m = m_T + m_{NT} \tag{S6}$$

With respect to the general definition of density, Equation S6 can be transformed to:

$$\rho V = \rho_T V_T + \rho_{NT} V_{NT} \tag{S7}$$

Introducing the volume shares now results in:

$$\rho = \rho_T \varphi_T + \rho_{NT} \varphi_{NT} \tag{S8}$$

As only two fractions are assumed, it applies that $\varphi_{NT} = 1 - \varphi_T$. Consequently, the volume shares of the tire fraction or non-tire fraction can be respectively expressed as:

$$\varphi_T = \frac{\rho_{NT} - \rho}{\rho_{NT} - \rho_T} \tag{S9}$$

$$\varphi_{NT} = \frac{\rho_T - \rho}{\rho_T - \rho_{NT}} \tag{S10}$$

In general, the mass share w_i of a fraction i can be deduced from the corresponding volume share φ_i following its definition, if the fraction density ρ_i and the overall density ρ are known:

$$\varphi_i = \frac{V_i}{V} = \frac{m_i \rho}{m \rho_i} = \frac{w_i \rho}{\rho_i} \Leftrightarrow w_i = \varphi_i \cdot \frac{\rho_i}{\rho} \tag{S11}$$

Now, we translate this considerations regarding individual TRWP to a particle sample, which contains TRWP, but might also contain further particles. The overall mass-related tire content of this sample (β_T) might be derived from the SBR content measured via TED-GC/MS, as it was done for the Tunnel sample (the Road simulator sample was treated differently due to unknown tire formulations, see main publication). If the density ρ of the sample is homogeneous and known or can be reasonably estimated, as is the case for the investigated density fractions, one can thus estimate the corresponding mass-related non-tire content (β_{NT}) that is associated with all TRWP. Considering that both tire fraction and non-tire fraction make up the sample's mass-related content of TRWP ($\beta_{TRWP} = \beta_T + \beta_{NT}$), assuming fixed, homogeneous densities for both fractions (ρ_T, ρ_{NT}) and utilizing the Equations above, β_T can be computed as follows:

$$\beta_{NT} = \beta_{TRWP} - \beta_T = \beta_T \left(\frac{1}{w_T} - 1\right) \xRightarrow{\text{Eq. S9, Eq. S11}} \beta_{NT} = \beta_T \left(\frac{\rho(\rho_{NT} - \rho_T)}{\rho_T(\rho_{NT} - \rho)} - 1\right) \quad \text{(S12)}$$

In some previous studies, it was not always clearly differentiated between volume-related and mass-related units when considering TRWP as mixtures of a tire fraction and a non-tire fraction. For instance, Unice et al.[11] assumed a mixed density of 1.8 g/cm³ for TRWP, based on a "50% road mineral encrustation content" – it remains ambiguous whether this refers to particle mass or volume. Further assumed was a tire tread density of 1.18 g/cm³ and 2.4 g/cm³ as the average density of road dust. A mixed density of 1.8 g/cm³ only results, if the content of 50% is volume-related (Equation S8). Otherwise, a mixed density ρ has to be determined by inserting $\varphi_{NT} = 1 - \varphi_T$ into Equation S8 and express φ_T as a function of w_T (Equation S11):

$$\rho = \rho_T \frac{w_T \rho}{\rho_T} + \rho_{NT}\left(1 - \frac{w_T \rho}{\rho_T}\right) \Leftrightarrow \rho = \rho_{NT}\left(1 - w_T + \frac{w_T \rho_{NT}}{\rho_T}\right)^{-1} \quad \text{(S13)}$$

Consequently, a mixed density of 1.6 g/cm³ instead of 1.8 g/cm³ results for the given scenario, if the 50% content of mineral encrustation is considered to be mass-related instead of volume-related.

S6 Merging size or velocity distributions of different density fractions

S6.1 Background and definitions

Based on settling experiments conducted separately for each density fraction, respective size and velocity distributions could be derived (cf. section S3 for computation method and section S10 for results). To further elucidate size and settling velocity of TRWP, these distributions might again be merged with respect to the estimated tire or TRWP content of the different fractions as weights (e.g. based on SBR measurements in case of the Tunnel sample), thus profiting from the enrichment of TRWP in certain density fractions (cf. Figure 2 of the main publication).

Considering a fraction i, the cumulative distribution of a quantity x, which can be settling velocity w_s or particle size by means of d_{eq} or ECD (cf. section S3), is hereafter denoted as $F_i(x)$. Several distributions might be merged by employing appropriate weight factors ω_i. In the following, this will be written as:

$$F(x) = \sum_i \omega_i \cdot F_i(x) \tag{S14}$$

In this study, a distribution $F_i(x)$ is derived empirically and thus consists of an ordered series of j data points of $x_{i,j}$ and a correspondingly derived cumulative probability $y_{i,j}$ (cf. section S3). In order to perform Equation S14, the probability $p_{i,j}$ is computed for each data point as the difference of cumulative probabilities multiplied by the fraction-specific weighting factor ω_i:

$$p_{i,j} = \omega_i \cdot (y_{i,j} - y_{i,j-1}) \tag{S15}$$

Then, all data points $(x_{i,j}, p_{i,j})$ are sorted by ascending x. Finally, the cumulative distribution $F(x)$ is computed from the sorted $p_{i,j}$ and the resulting series of cumulative probabilities is normed to $]0,1]$.

S6.2 Derivation of weighting factors ω_i for different target distributions

The weighting factors ω_i required for merging the distributions of several density fractions i have to be derived carefully depending on the targeted scenario and distribution characteristics. They are independent of the considered value for x, yet they differ depending on whether the targeted distribution is related to particle mass, volume, surface area or particle number. Moreover, different distributions can be derived for the tire content and the TRWP content of a sample, respectively, since the latter entails varying content of the TRWP-associated non-tire matter, e.g. minerals, for the different density fractions (cf. section S5).

For the Tunnel sample, the tire mass content β_T is estimated from SBR measurements. β_{TRWP} might then be estimated from β_T under the assumption of respective mean density ρ and tire and non-tire densities ρ_T and ρ_{NT}, respectively (cf. Equation S12 and Equation 1 of the main manuscript). For the Road simulator sample, β_T is already estimated from ρ, ρ_T and ρ_{NT} via assuming 100% TRWP content – except for the upper density fraction, which can contain pure stone dust. Therefore, the possible range of TRWP content is indicated instead (cf. section S7 and Figure 1 of the main manuscript).

In the following, the composition of distributions for mass, volume, surface area and particle number is exemplified for tire content β_T. If possible, β_T can be replaced by β_{TRWP} in order to relate to full TRWP. If only tire content β_T is considered, surface area and particle number practically become hypothetical quantities as measured particles are weighted with respect to the supposed tire content. In general, distributions of density fractions are merged using the mass, volumetric, surface area or particle number-related tire content of the density fractions as weighting factors, respectively. For these four different scenarios, Table S3 comprises the expressions for $F_i(x)$ and the weighting factors ω_i to be applied with Equation S14.

Table S3. Measured distributions and corresponding weighting factors for composing target distributions related to tire content in terms of mass, volume, surface area or particle number.

Case index	Targeted distribution (referring to tire content)	Employed measured distributions, $F_i(x)$	Weighting factors, ω_i
1	$F_{T,mass}(x)$, related to mass	$F_{mass,i}(x) = \rho_i \cdot F_{vol,i}(x)$	$\omega_{T,mass,i} = w_i \beta_{T,i}$
2	$F_{T,vol}(x)$, related to volume	$F_{vol,i}(x)$	$\omega_{T,vol,i} = \frac{w_i}{\rho_i} \beta_{T,i} = \frac{w_i V_{exp,i}}{m_{exp,i}} \beta_{T,i}$
3	$F_{T,area}(x)$, related to surface area	$F_{area,i}(x)$	$\omega_{T,area,i} = \frac{w_i A_{exp,i}}{m_{exp,i}} \beta_{T,i}$
4	$F_{T,num}(x)$, related to particle number	$F_{num,i}(x)$	$\omega_{T,num,i} = \frac{w_i N_{exp,i}}{m_{exp,i}} \beta_{T,i}$

$F_i(x)$ always has to be expressed by means of a measured distribution. Regarding a mass-related target distribution, it is thus ascribed to $F_{vol,i}(x)$ multiplied by the density fractions mean density ρ_i. The weighting factor always includes the respective mass share of total sample mass, w_i, and tire content $\beta_{T,i}$ of the considered density fraction. Considering cases 2, 3 and 4, the relation to mass has to be corrected accordingly. This correction is approximated by computing the cumulated volume $V_{exp,i}$, surface area $A_{exp,i}$ or particle number $N_{exp,i}$ of all measured particles – respecting cutoffs and correction factors as introduced previously (cf. section S3 and Figure S1) – and dividing them through the investigated particle dose $m_{exp,i}$. When considering volume (Case 2), the mean fraction density ρ_i can be used for correction as well, since volume and mass are directly proportional if a constant density is assumed.

S6.3 Uncertainty and confidence intervals

To illustrate the uncertainty associated with a merged distribution, several 95% confidence intervals (CIs) were derived, which are briefly described in the main manuscript as well. The 'mean data CI' is constructed from the different CIs, that were already computed individually for each fraction by bootstrapping the measurement data as explained in section S3 (also see Figure S3 and results given in section S10). The lower and upper limit distributions of the CIs of all fractions were then merged according to Equation S14, respectively, in order to yield the lower and upper limits constituting the 'mean data CI'.

Still, both the merged distribution itself and the 'mean data CI' are based on the preliminary assumption that the size and settling velocity distributions of TRWP within a fraction (either weighted with respect to incorporated tire-mass or TRWP mass) reflect the average size and settling velocity distributions measured for the respective fraction overall. This simplification is necessary, since particles tracked during a settling experiment cannot be individually identified as TRWP or not, yet it is contrasted by computing another CI, which will be denoted as the 'edge case CI' in the following.

The 'edge case' refers to an opposite extreme assumption, which thus marks an estimate of maximum associated uncertainty. Here, it is assumed that TRWP constitute the smallest or largest (and slowest or fastest settling) particles of a density fraction, respectively. Based on the TRWP content of the fractions, the lower and upper limits of the 'mean data CI' were truncated, scaled and then merged, respectively. If distributions related to particle count or surface area are considered, the TRWP content, which originally relates to mass (and volume when assuming narrow density fractions), has to be transformed accordingly. This transformation was executed based on the settling data as well, by computing and then interrogating volume-related distributions of particle count or estimated particle surface area per dosed mass (cf. Zenodo repository[12]). Consequently, different TRWP contents result for the two edge cases, e.g. the transformed TRWP content with respect to surface area or count is increased when assuming that TRWP account for the smallest particles of a fraction (and vice versa).

Additionally, both the 'mean data CI' and the 'edge case CI' were extended via bootstrapping various inputs, e.g. mass shares of fractions and tire contents, as is specified in section S6.4. The extended 95% CIs were derived from 2000 parallel resamples of these inputs and are denoted as 'mean data CI w/ resampling' and 'edge case CI w/ resampling', respectively.

S6.4 Implementation and assumptions

To merge size and velocity distributions measured for different fractions with respect to tire or TRWP mass, Equation S14 has to be implemented together with respective weighting factors according to Table S3. This implementation requires certain specifications and assumptions that will be detailed in the following. Beside the measured distributions of particle size or settling velocity, further inputs are needed, such as average density, tire or TRWP content and mass share of each fraction. Depending on the targeted distribution, cumulated surface area $A_{exp,i}$ or particle number $N_{exp,i}$ might be required as well (cases 3 and 4, Table S3). Regarding volume-specific distributions, weighting factors $\omega_{T,vol,i}$ were computed from w_i, ρ_i and $\beta_{T,i}$ according to the first of two approaches (case 2, Table S3).

All mass shares of the different fractions were set according to the fractionation results. For deriving extended CIs (see section S6.3), the mass shares were resampled from normal distributions as defined by the measured standard deviations (cf. Table S4) – except for the sieved size fractions as well as the density fractions below 1.3 g/cm³ of the Tunnel sample, for which no parallel fractionation steps were feasible (cf. section S1).

The densities of the density fractions (ρ_i in Table S3) were specified as the average center points between the measured density limits (cf. Table S1). For extended CIs, fraction densities were

resampled from normal distributions around these center points, restricted by the measured density limits and with the standard deviation set to the quarter of the range between these limits.

The terminal density fractions had to be considered separately, as they lack a second density limit: The lower terminal fractions constitute only small shares of the overall mass of both samples, respectively. For the Road simulator sample, the density range of the < 1.6 g/cm³ fractions was virtually set to 1.5–1.6 g/cm³ with an assumed average density of 1.55 g/cm³. Resampling for extended CIs was then conducted as described above for the non-terminal density fractions. For the lower terminal fraction of the Tunnel sample, < 1.2 g/cm³, the upper limit of 1.2 g/cm³ was assumed as average density and excluded from resampling, as the fraction's mass share is marginal (< 0.4%) and it appears from the optical microscope pictures (cf. Figure S19) as if the fraction largely consists of TRWP. In this case, the respective densities of the tire material associated with these particles probably fall slightly below 1.2 g/cm³, still it should be an appropriate assumption for this fraction's average density as well as for the tire tread density throughout.

Regarding the upper terminal density fractions, the mean densities from pycnometer measurements were specified as average densities – together with measured standard deviations, which were then used to define normal distributions for resampling again (cf. section S2 for results of pycnometer measurements). Resampling was restricted to the range between the lower density limits of the upper terminal fractions, 2.0 g/cm³ for the Road simulator sample and 2.14 g/cm³ for the Tunnel sample, and the assumed average density of the non-tire material of TRWP, 2.684 g/cm³ for the Road simulator sample and 2.503 g/cm³ for the Tunnel sample (cf. section S2).

TRWP mass content (β_{TRWP}) can be estimated from tire mass content (β_T) via Equation 1 of the main manuscript. β_{TRWP} is not only required for composing distributions related to TRWP, but also for computing the 'edge case CIs' (cf. section S6.3). As input for Equation 1, an average density of the respective TRWP particles has to be assumed – besides the average densities of tire and non-tire material that were already estimated and specified before for both samples (cf. section S2). The average densities of TRWP were equated with the respective average fraction densities described previously – except for the upper terminal density fraction of both samples: For the Road simulator sample, 2.245 g/cm³ was assumed as the average TRWP density of the > 2.0 g/cm³ density fraction. It is the center between 2.0 g/cm³ and 2.49 g/cm³, which are the limits of TRWP density and represent either minimum (22.3%) or maximum TRWP content (100%) of this fraction. As detailed elsewhere (e.g. Figure 1 of main publication and Table S4), the > 2.0 g/cm³ fraction of the Road simulator sample possibly contains larger amounts of stone dust that is not incorporated in TRWP. Since additional information are missing, the resampling for the 'edge case CIs' was performed from a uniform distribution between the limits 2.0 g/cm³ and 2.49 g/cm³.

For the Tunnel sample, the average TRWP density of the upper terminal density fraction was set to 2.2 g/cm³, which has been described as an upper limit for TRWP density in previous studies[1,13]. A higher TRWP density is rather unlikely, when acknowledging the low SBR content measured for this fraction (0.53 mg/kg) and conceptualizing TRWP properties as spanning a continuous spectrum. Resampling was restricted to the range of the TRWP density's theoretical limits of 2.14 and 2.49 g/cm³. It was conducted from a normal distribution with the standard deviation set to 0.06 g/cm³ – the difference between the center at 2.2 g/cm³ and the lower density limit. Apart from the implementation with respect to β_{TRWP}, assumed differences between the average density of the

upper terminal density fractions overall and of the contained TRWP were not considered when handling distributions of size and settling velocity measured for these two fractions.

Figure S6 depicts the distributions from which average densities and TRWP densities were resampled for the different density fractions of both samples for deriving the 'extended CIs'. Details and reasoning for these choices were described directly above.

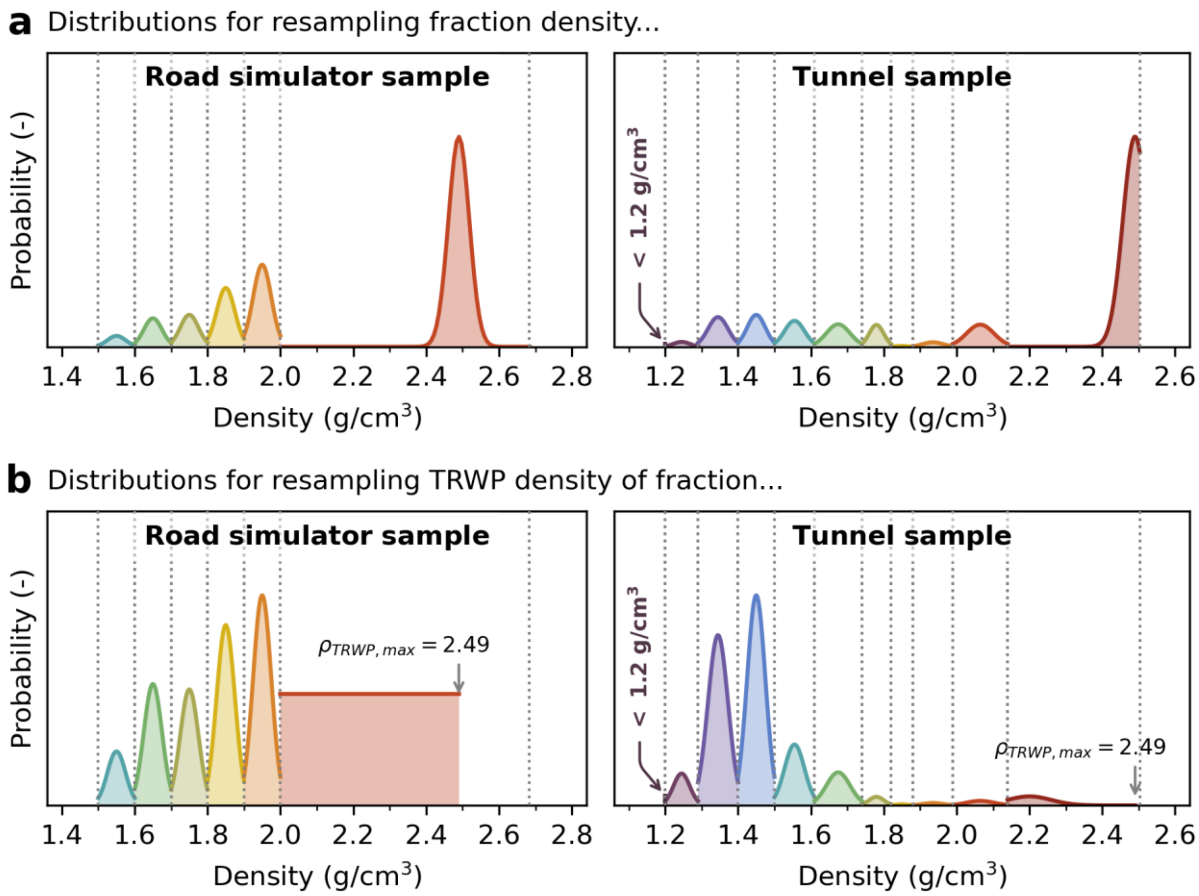

Figure S6. Assumed probability distributions for resampling (a) fraction densities and (b) TRWP densities for the different density fractions of the Road simulator (left side) and Tunnel sample (right side). For presentation purposes, probability maxima of all distributions are scaled to resemble the fractions' (a) mass shares or (b) share of overall tire mass, respectively.

The tire mass content β_T was computed differently for both samples (cf. 'Estimation of tire and TRWP mass contents' section of the main manuscript): Regarding the Road simulator sample, β_T was determined from β_{TRWP} by assuming 100% TRWP content for every except the upper terminal density fraction, for which β_T was determined from the measured fraction density and the densities assumed for tire (ρ_T) and non-tire material (ρ_{NT}). β_{TRWP} was indirectly resampled in case of the upper terminal density fraction via resampling TRWP density from a uniform distribution as has been described previously. For the Tunnel sample, β_T was estimated from measured SBR contents and thus resampled for 'edge case CIs' from normal distributions by assuming relative standard deviations of 9% for the SBR measurements (cf. S4 and Table S4). With respect to the 'edge case CI', β_{TRWP} was again indirectly resampled via resampling TRWP densities for the different fractions.

Further assumptions were necessary with respect to three density fractions of the Tunnel sample, which could not be investigated in settling experiments due to insufficient sample quantity. These missing size and velocity distributions were completed by assuming distributions measured for neighboring fractions. Namely, distributions for the < 1.2 g/cm³ fraction were taken from data on the 1.2–1.3 g/cm³ fraction, for the 1.8–1.9 g/cm³ fraction from data on the 1.7–1.8 g/cm³ fraction and for the 1.9–2.0 g/cm³ fraction from data on the 2.0–2.1 g/cm³ fraction, respectively.

If required (cf. Table S3), cumulated surface area $A_{exp,i}$ or cumulated particle number $N_{exp,i}$ of a density fraction i were computed from the particle data of the settling experiments by relating the desired quantity – either surface area or count – to the dosed sample mass, sorting the detected particles by descending size and then compiling cumulative distributions with respect to the detected particle volume. All of these distributions are available from the associated Zenodo repository[12]. Similar to deriving distributions of particle size and settling velocity, cutoffs for velocity and particle size were considered in order to combine the sets of experiments with different runtime. Therefore, each distribution was derived in triplicates using individual cutoffs for particle size ($d_{eq,Bagheri}$), equivalent circular diameter (ECD) and settling velocity (see section S3). These triplicates showed only minimal differences, which additionally substantiates the validity of the method of computing individual cutoffs. When similarly deriving cumulated particle volumes (also included in the Zenodo repository[12]) for the different fractions, they can be multiplied with average fraction densities in order to obtain estimates of the recovery of the nominally dosed particle mass. These recoveries are within the expected range considering the geometry of the experimental setup and the narrow depth of field of the imaging system (ca. 0.5 mm)[6], yet they reveal notable differences between the different investigated fractions as depicted in Figure S7. The exceptional low recovery recorded for the < 1.6 g/cm³ fraction of the Road simulator sample possibly reflects the reduced number of experiments carried out due to insufficient sample mass. The same fraction also exhibits a comparably large median particle size, which would usually require an increased amount of experiments. This deficit is also recognizable from wider confidence intervals of the derived size and velocity distributions (cf. Figure S46). To account for the observed variations of the estimated recoveries between different density fractions, $A_{exp,i}$ or $N_{exp,i}$ were corrected accordingly (dividing by the recovery), when merging the size or velocity distributions of the different density fractions of a sample.

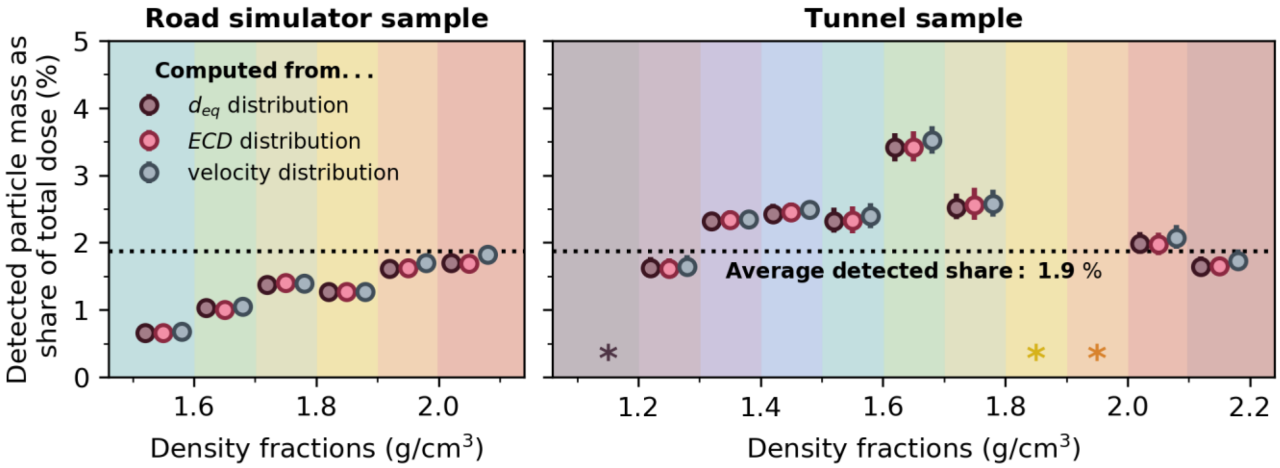

Figure S7. Share of nominally dosed particle mass detected during settling experiments with different density fractions as computed from distributions of d_{eq}, ECD or settling velocities.

Regarding the lower and upper boundaries of the 'edge case CIs', surface area $A_{exp,i}$ and particle number $N_{exp,i}$ were derived from the respective distribution per particle dose (see Zenodo repository[12]) at the probabilities of β_{TRWP} (assuming TRWP constitute only the largest particles of a fraction) and $1-\beta_{TRWP}$ (assuming TRWP constitute only the smallest particles of a fraction). In general, $A_{exp,i}$ and $N_{exp,i}$ were as well resampled for extended CIs: At the relevant probability (1 for whole fraction or β_{TRWP} and $1-\beta_{TRWP}$ for the 'edge case CIs'), the mean value and the 95% CI were extracted from the distribution per particle dose. Then, resampling was conducted from a normal distribution centered around the mean value, limited to the 95% CI range and with the standard deviation set to a quarter of this range.

S7 Tabular results of fractionation procedures

Table S4 comprises mass shares, SBR and NR measurements as well as estimated tire and TRWP content for both samples and all considered fractions. Based on a replicate measurement, a standard deviation of 9% is assumed for measuring SBR via TED-GC/MS (cf. section S4). For each density fraction, exact density limits are given in Table S1. TRWP content is estimated from mean density and tire content (see section S5) assuming a tire density of 1.200 g/cm³ and non-tire densities of 2.684 g/cm³ and 2.503 g/cm³ for the Road simulator and Tunnel sample, respectively (see section S2). As described in the main text, tire content is estimated from SBR for the Tunnel sample and from mean densities via assuming 100% TRWP content for the Road simulator sample (range given for the upper density fraction).

Table S4. Mass shares and measured SBR and NR content for both samples and all considered size and density fractions.

Sample/Fraction			Mass share (wt%)		SBR content (mg/g)		NR content (mg/g)		Estimated content (wt%)			
									Tire		TRWP	
			Mean	SD	Mean	SD	Mean	SD	Mean	SD	Mean	SD
Road simulator sample	Full sample		-	-	80.16	7.21	7.68	n.d.	n.d.*	n.d.*	n.d.†	n.d.†
Road simulator sample	Density fractions (g/cm³)	< 1.6	2.57	0.68	88.50–90.50	8.06	7,90–19,6	n.d.	59.16‡	n.d.	100.00	n.d.
Road simulator sample	Density fractions (g/cm³)	1.6–1.7	6.75	0.49	108.30	9.75	14.20	n.d.	50.67‡	n.d.	100.00	n.d.
Road simulator sample	Density fractions (g/cm³)	1.7–1.8	7.57	0.86	46.11	4.15	11.77	n.d.	43.16‡	n.d.	100.00	n.d.
Road simulator sample	Density fractions (g/cm³)	1.8–1.9	13.94	1.71	31.93	2.87	4.68	n.d.	36.45‡	n.d.	100.00	n.d.
Road simulator sample	Density fractions (g/cm³)	1.9–2.0	19.44	1.81	34.20	3.08	5.60	n.d.	30.44‡	n.d.	100.00	n.d.
Road simulator sample	Density fractions (g/cm³)	> 2.0	49.74	3.00	30.22	2.72	2.10	n.d.	6.30‡	n.d.	22.34–100.00	n.d.
Tunnel sample	Full sample		-	-	9.82	0.88	6.92	n.d.	8.69	0.78	n.d.†	n.d.†
Tunnel sample	< 20 µm		14.77	-	15.86	1.43	6.58	n.d.	14.03	1.27	n.d.†	n.d.†
Tunnel sample	20–250 µm		53.12	-	15.91	1.43	11.60	n.d.	14.08	1.27	n.d.†	n.d.†
Tunnel sample	Density fractions (g/cm³)	< 1.2	0.39	-	43.25	3.89	151.92	n.d.	38.27	3.44	38.27	3.44
Tunnel sample	Density fractions (g/cm³)	1.2–1.3	0.68	-	82.07	7.39	140.54	n.d.	72.63	6.54	78.05	7.02
Tunnel sample	Density fractions (g/cm³)	1.3–1.4	4.18	0.22	71.54	6.44	43.63	n.d.	63.31	5.70	79.84	7.19
Tunnel sample	Density fractions (g/cm³)	1.4–1.5	4.50	0.27	82.11	7.39	48.21	n.d.	72.66	6.54	108.64	9.78
Tunnel sample	Density fractions (g/cm³)	1.5–1.6	3.66	0.68	29.20	2.63	19.23	n.d.	25.84	2.33	46.02	4.14
Tunnel sample	Density fractions (g/cm³)	1.6–1.7	3.17	0.06	18.31	1.65	6.68	n.d.	16.20	1.46	35.59	3.20
Tunnel sample	Density fractions (g/cm³)	1.7–1.8	3.09	0.17	4.88	0.44	1.62	n.d.	4.32	0.39	11.54	1.04
Tunnel sample	Density fractions (g/cm³)	1.8–1.9	0.11	0.00	16.51	1.49	7.61	n.d.	14.61	1.31	44.94	4.04
Tunnel sample	Density fractions (g/cm³)	1.9–2.0	0.63	0.02	6.61	0.59	1.51	n.d.	5.85	0.53	21.63	1.95
Tunnel sample	Density fractions (g/cm³)	2.0–2.1	3.12	0.12	2.52	0.23	0.46	n.d.	2.23	0.20	11.43	1.03
Tunnel sample	Density fractions (g/cm³)	> 2.1	29.60	0.67	0.53	0.05	0.15	n.d.	0.47	0.04	3.72	0.33
Tunnel sample	> 250 µm		32.11	-	0.65	0.06	1.32	n.d.	0.57	0.05	n.d.†	n.d.†

* mean density of full sample was not measured

† no density fraction, thus TRWP density is variable and cannot be enclosed

‡ estimated from densities instead of measured SBR content

S8 Optical microscope images

S8.1 Road simulator sample

In this section, optical microscope images of the Road simulator sample are presented. Figure S8 shows the full sample, whereas Figures S9–S14 show the different density fractions, that were investigated. Scale bars are indicated column-wise in the first figure of each page, respectively. At the two higher magnifications, 2 to 5 images with different focus layers were acquired and stacked using a Python script, if the observed particles could not be entirely focused.

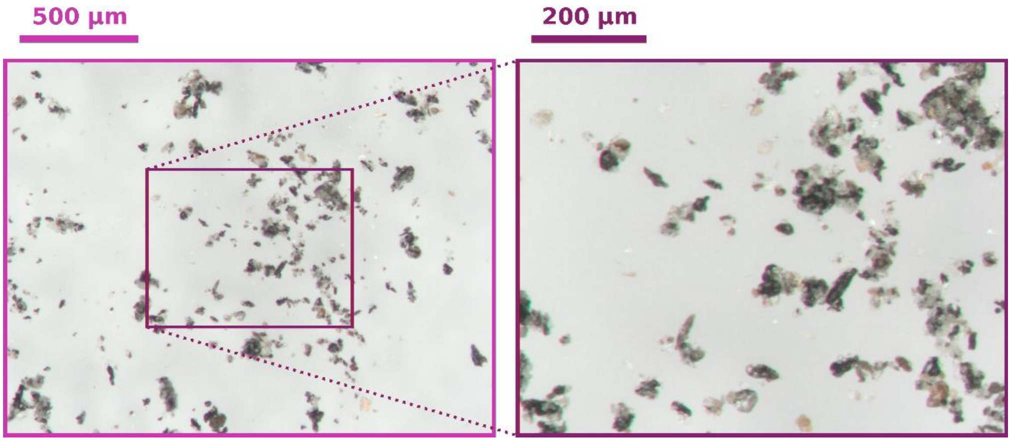

Figure S8. Optical microscope image of Road simulator sample (full sample).

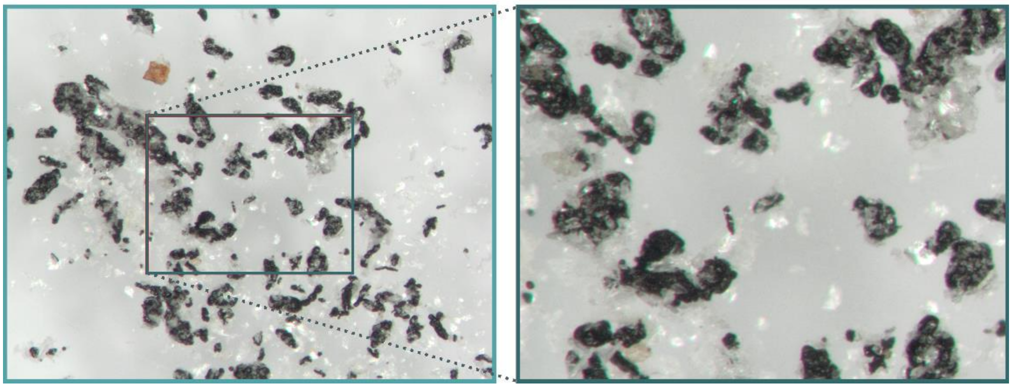

Figure S9. Optical microscope image of Road simulator sample (density fraction, < 1.6 g/cm³).

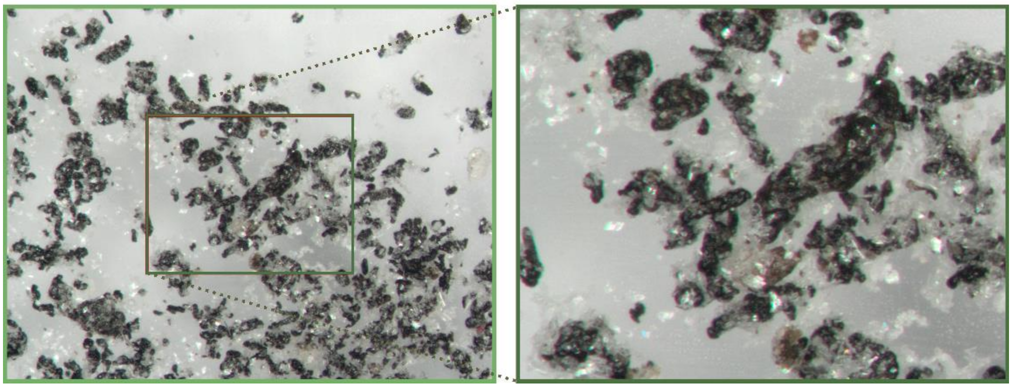

Figure S10. Optical microscope image of Road simulator sample (density fraction, 1.6–1.7 g/cm³).

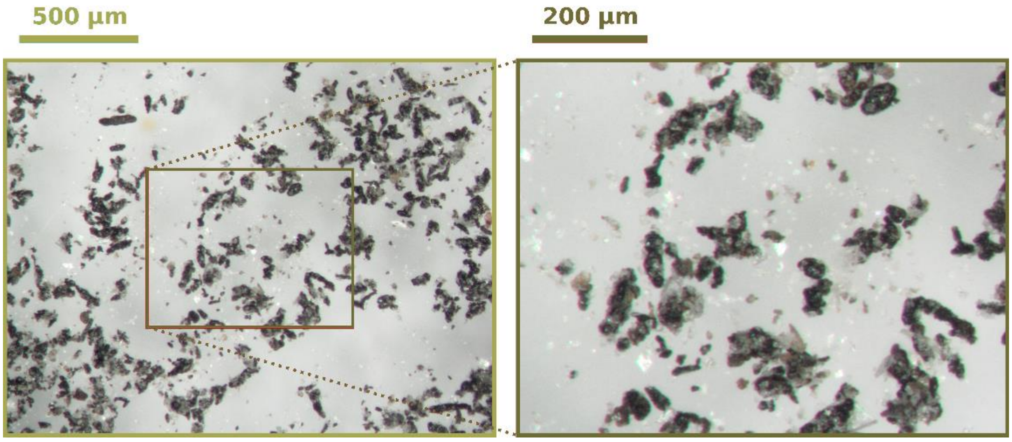

Figure S11. Optical microscope image of Road simulator sample (density fraction, 1.7–1.8 g/cm³).

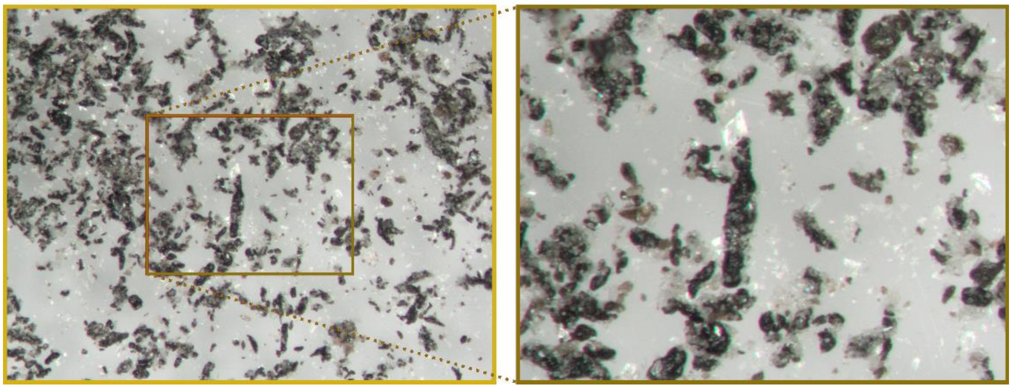

Figure S12. Optical microscope image of Road simulator sample (density fraction, 1.8–1.9 g/cm³).

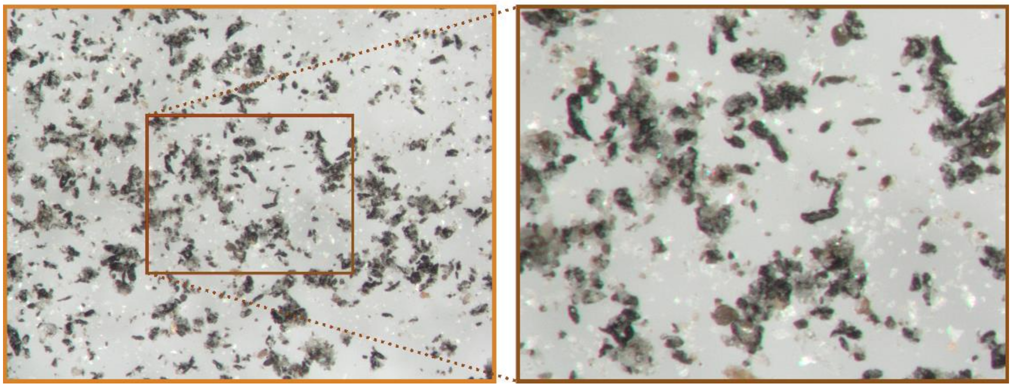

Figure S13. Optical microscope image of Road simulator sample (density fraction, 1.9–2.0 g/cm³).

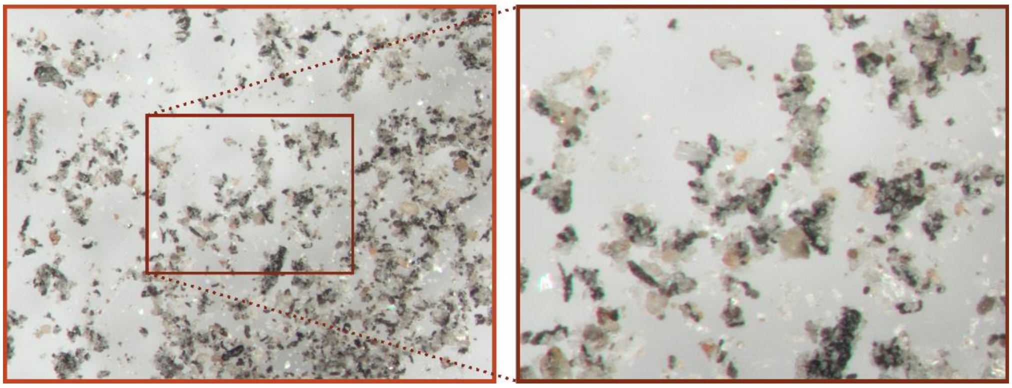

Figure S14. Optical microscope image of Road simulator sample (density fraction, > 2.0 g/cm³).

S8.2 Tunnel sample

In the following, optical microscope images of the Tunnel sample are presented. Figure S15 shows the full sample, Figures S16–S18 show the three different size fractions and Figures S19–S29 the density fractions of the size fraction between 20 and 250 µm. Scale bars are indicated column-wise in the first figure of each page. If necessary, 2–5 images were acquired and stacked at higher magnifications.

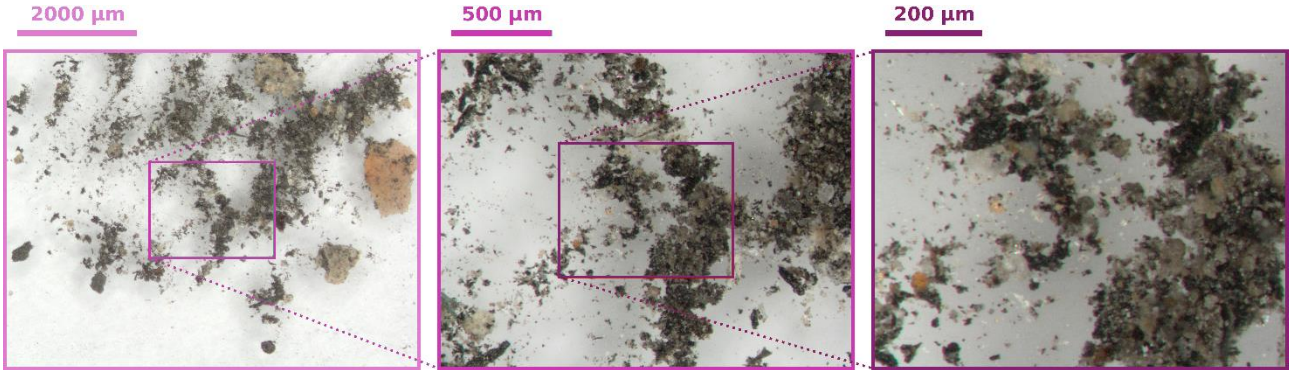

Figure S15. Optical microscope image of Tunnel sample (full sample).

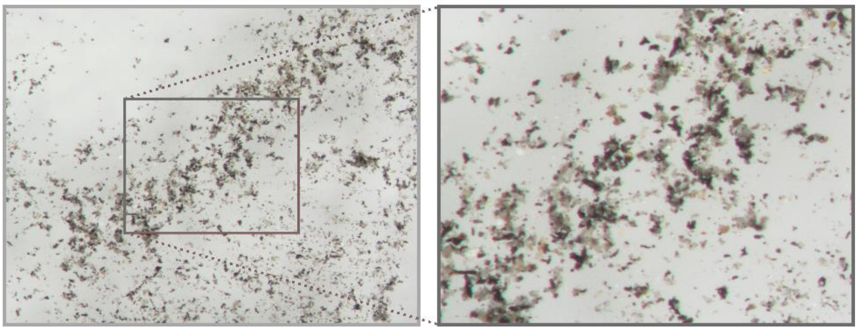

Figure S16. Optical microscope image of Tunnel sample (size fraction, < 20 µm).

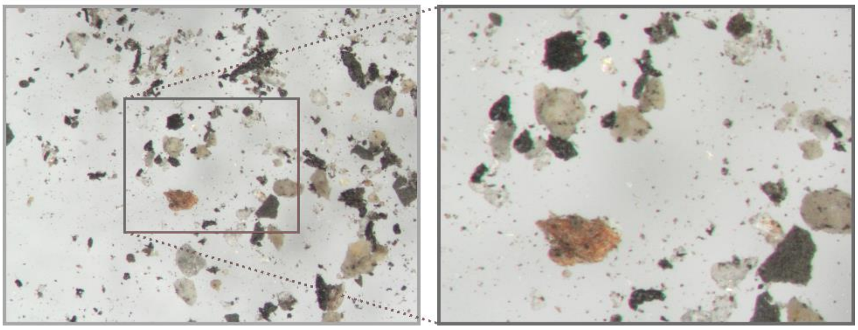

Figure S17. Optical microscope image of Tunnel sample (size fraction, 20–250 µm).

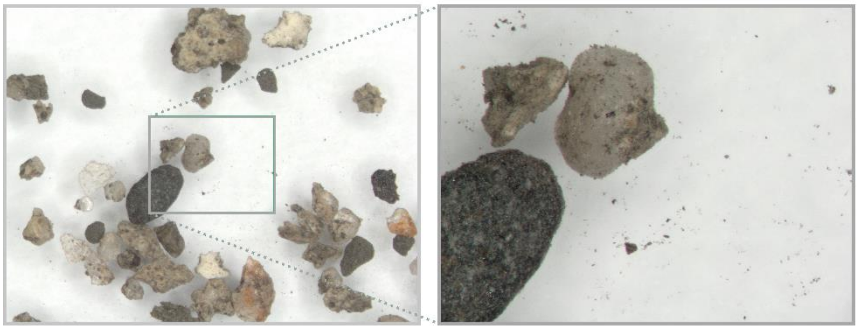

Figure S18. Optical microscope image of Tunnel sample (size fraction, > 250 µm).

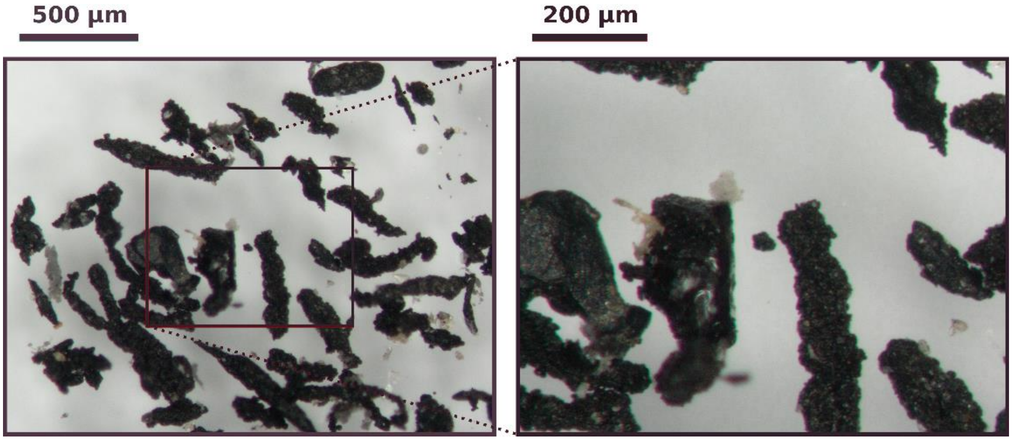

Figure S19. Optical microscope image of Tunnel sample (density: < 1.2 g/cm³, size: 20–250 µm).

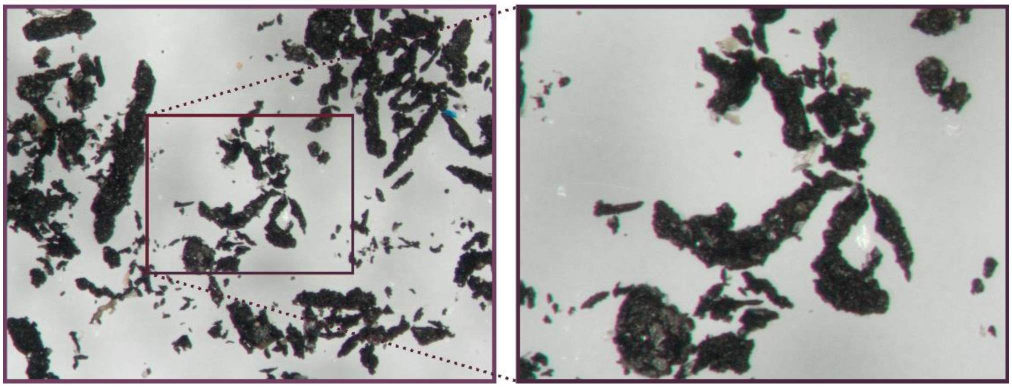

Figure S20. Optical microscope image of Tunnel sample (density: 1.2–1.3 g/cm³, size: 20–250 µm).

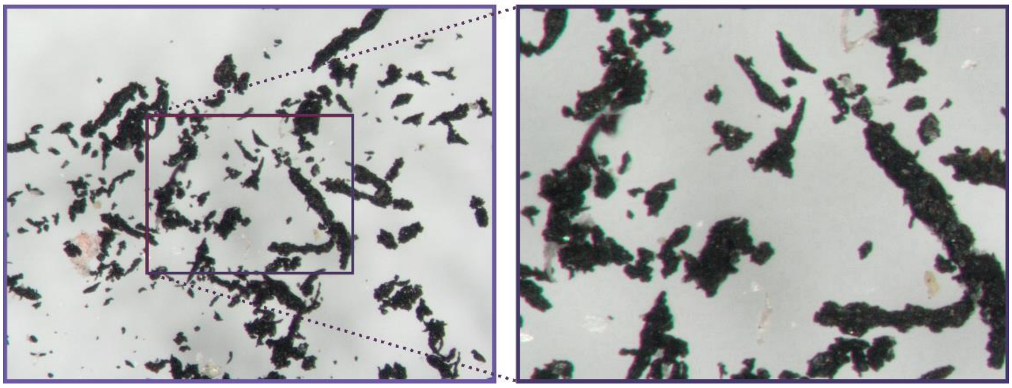

Figure S21. Optical microscope image of Tunnel sample (density: 1.3–1.4 g/cm³, size: 20–250 µm).

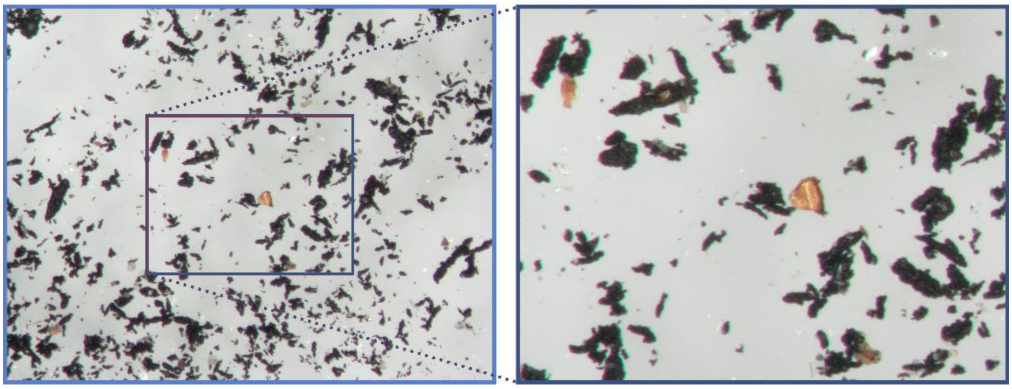

Figure S22. Optical microscope image of Tunnel sample (density: 1.4–1.5 g/cm³, size: 20–250 µm).

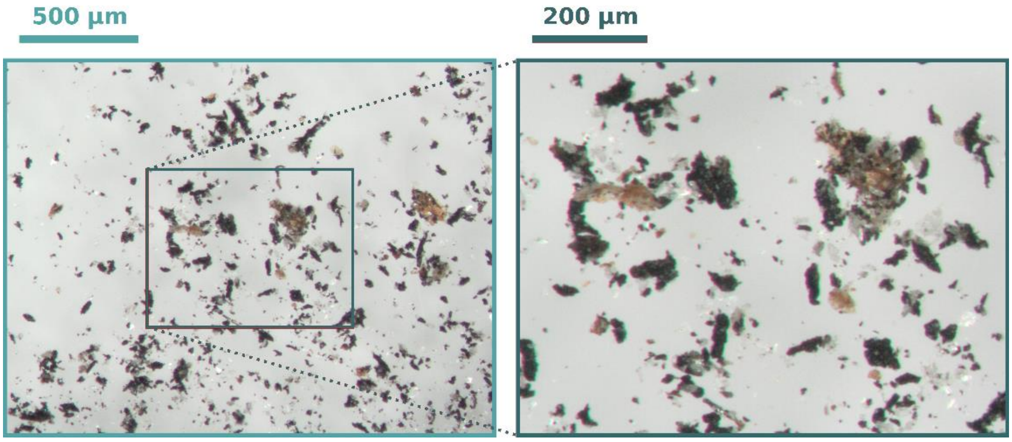

Figure S23. Optical microscope image of Tunnel sample (density: 1.5–1.6 g/cm³, size: 20–250 µm).

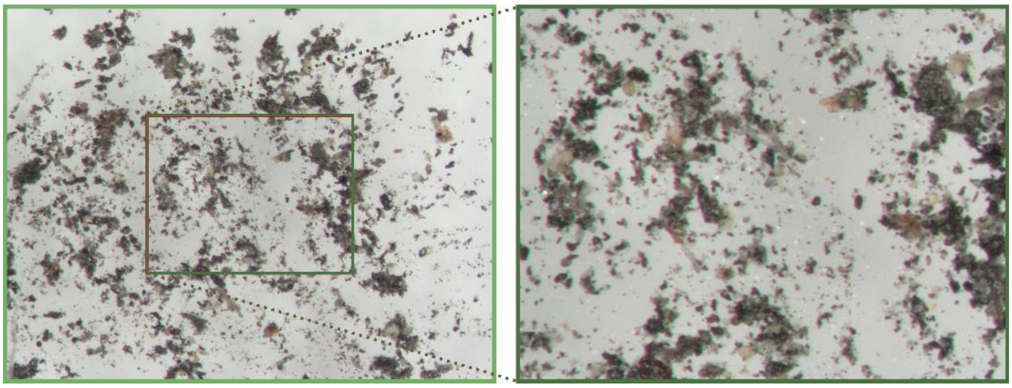

Figure S24. Optical microscope image of Tunnel sample (density: 1.6–1.7 g/cm³, size: 20–250 µm).

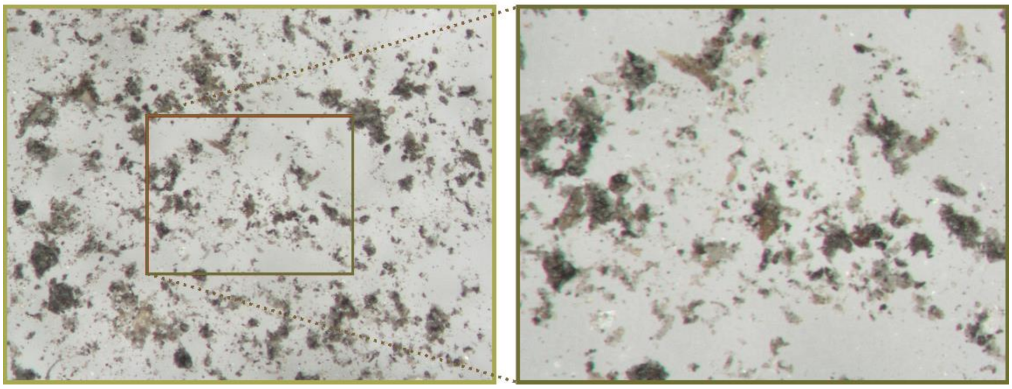

Figure S25. Optical microscope image of Tunnel sample (density: 1.7–1.8 g/cm³, size: 20–250 µm).

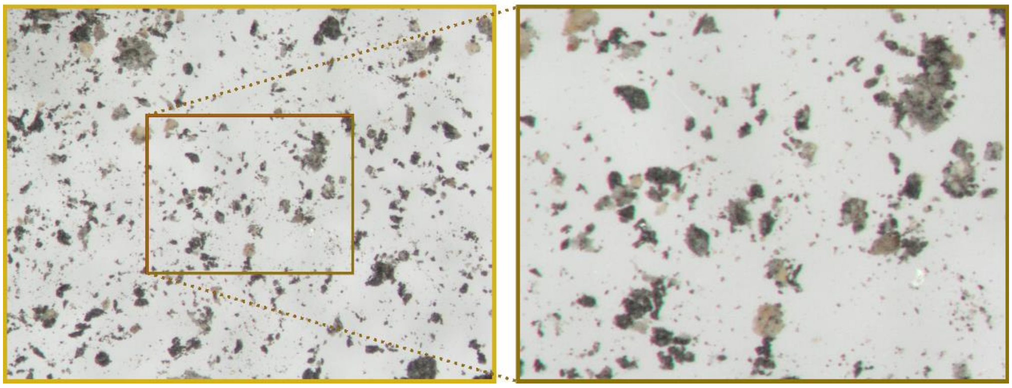

Figure S26. Optical microscope image of Tunnel sample (density: 1.8–1.9 g/cm³, size: 20–250 µm).

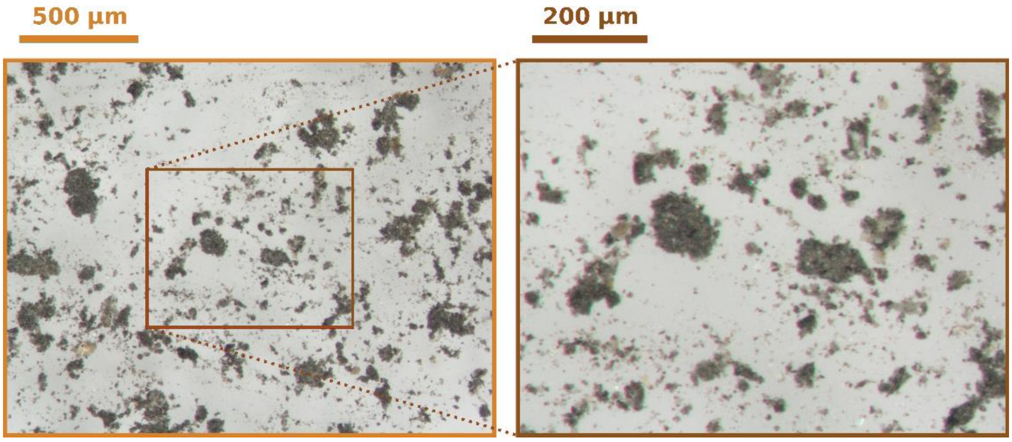

Figure S27. Optical microscope image of Tunnel sample (density: 1.9–2.0 g/cm³, size: 20–250 µm).

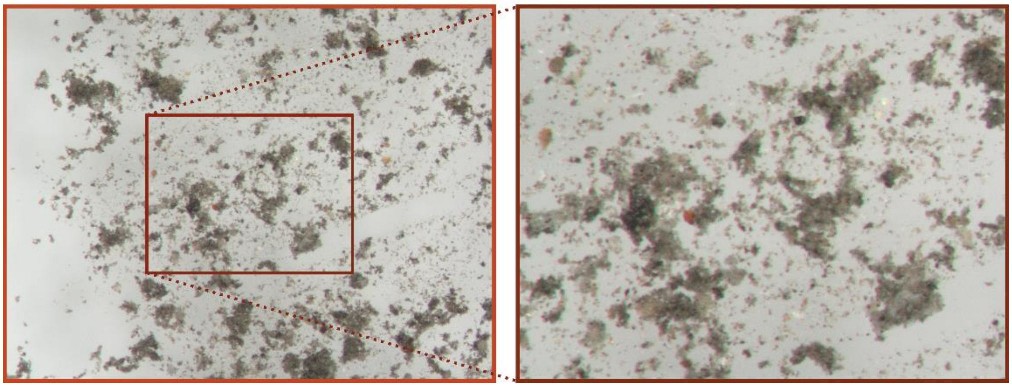

Figure S28. Optical microscope image of Tunnel sample (density: 2.0–2.1 g/cm³, size: 20–250 µm).

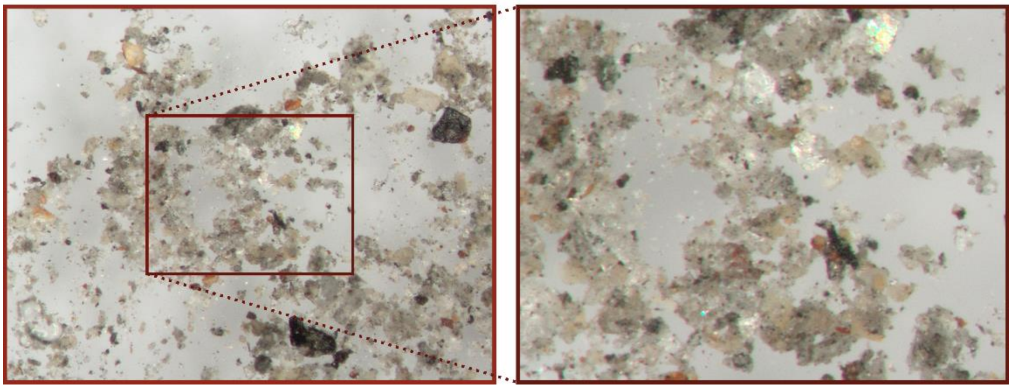

Figure S29. Optical microscope image of Tunnel sample (density: > 2.1 g/cm³, size: 20–250 µm).

S9 Data quality of settling velocity measurements

S9.1 Visual inspection of particles and contaminations

In total, 123,572 particles were detected and tracked. 30,759 of them were further considered for composing distributions as they met a respective cutoff for settling velocity or particle size (cf. sections S3 and S10) and will be denoted as evaluated particles. To exclude distinct contaminants – basically long and thin fibrous particles as the samples potentially include all other particle morphologies – or correct possible detection errors, a visual inspection of suspect particle contours was carried out by a human observer. Detection errors occur only rarely, e.g. if a particle contour is detected incompletely due to a partially translucent depiction or if overlapping particles are detected, although usually prevented by the detection algorithm. Despite stabilization of all suspensions with a detergent mix (NovaChem SF100, Postnova Analytics, Germany), as detailed in a previous publication[3], loosely agglomerated particles can be observed seldom, too.

Suspect particles were pre-filtered in order to reduce the workload. First, only particles with an average ECD above 30 µm were considered, as very small contours usually not allow any visual classification. Moreover, they had to meet low default cutoffs for size (ECD > 50 µm for the ultimate set of experiments) and settling velocity (> 0.2 mm/s for the penultimate and > 0.5 mm/s for the ultimate set of experiments). Now, potential contaminants and incorrect detections were further filtered to either have an average circularity below 0.6, a relative standard deviation of the ECD of more than 4% or include an outlier detection with an ECD further than 2.5 times the standard deviation from the mean ECD mean. Overall, 11,288 particles were visually classified, of which 625 (5.5% of suspects) were corrected by removing erroneous detections and 52 (0.5% of suspects) were removed – 16 as contaminations, 9 as incorrect detections and 27 as agglomerates. The remaining suspect particles (94.0% of suspects) were classified as valid measurements.

4805 (15.6%) of the 30,759 evaluated particles were visually classified – excluding 32 particles (0.1%) and correcting 192 (0.6%). The shares of excluded particles are depicted for each investigated density fraction in Figure S30. Interestingly, agglomerates were only found for fraction 1.4–1.5 g/cm³ of the Tunnel sample, still only 0.6% of the particles were excluded for this fraction.

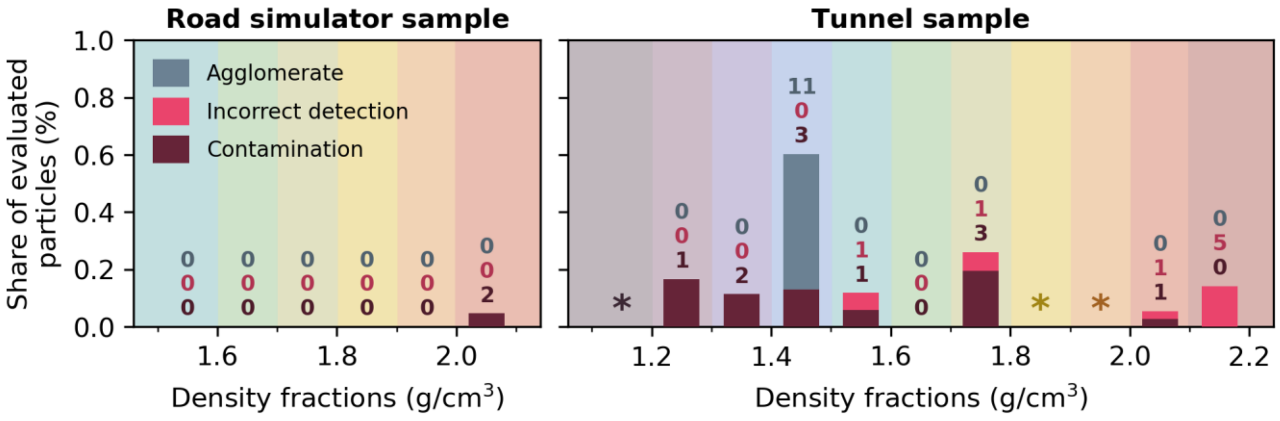

Figure S30. Share of evaluated particles excluded as contaminations, incorrect detections or agglomerates for density fractions of both samples. Absolute numbers indicated above the bars. Non-investigated fractions are indicated (*).

In case of the Tunnel sample, another six detected particles were classified as contaminations and excluded from further evaluation, since their contours have maximum inscribed circle diameters above 250 µm, which conflicts with the upper limit of the investigated size fraction (20–250 µm). Figure S31 shows all respective particles (note scale bar). The two largest particles are especially noticeable: Both might be large, tight agglomerates or include material from the used membrane filters scraped during sample handling.

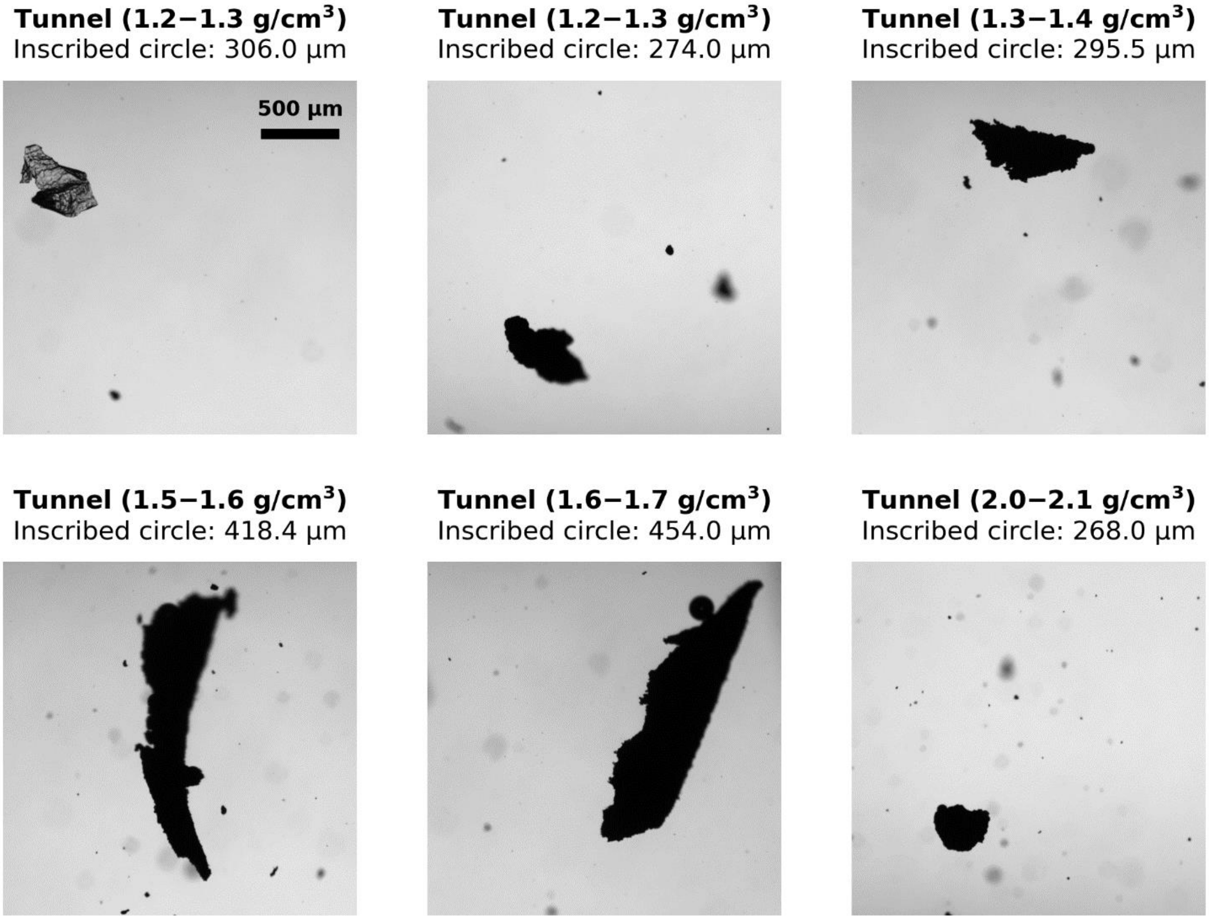

Figure S31. Raw image sections of all particles with inscribed circle diameters larger than 250 µm from density fractions of the Tunnel sample.

S9.2 Applying empirical model for particle-particle interactions

An empirical model for describing a potential increase of settling velocity due to interactions between particles was applied to all obtained settling data. The model was previously proposed with respect to the used measuring setup and calibrated according to spherical particles.[6] Thus, modeled deviations are not used to correct measured values directly. Instead, the settling velocity of a particle for which the modeled velocity deviation exceeds both an absolute (> 0.01 mm/s) as well as a relative threshold (> 5% of the measured velocity) is corrected based on further data gathered for the same fraction: The velocity is assigned the average value of the 5 most similar particles in terms of particle size (d_{eq}), which meet the criteria. Apart from particle tracking data, only particle density is required as additional model input[6]: It was estimated conservatively as the minimum for each fraction (down to 1.0 g/cm^3), which results in higher velocity deviations as output.

The following figures present the particles, which were excluded based on these criteria and in turn corrected, as well as the particles which were validly measured according to the empirical model. Figures S32–S37 and Figures S38–S45 show the results for all investigated density fractions of the Road simulator sample and Tunnel sample (20–250 µm size fraction only), respectively. Three density fractions (< 1.2, 1.8–1.9 and 1.9–2.0 g/cm³) of the Tunnel sample could not be investigated in settling experiments due to insufficient sample mass.

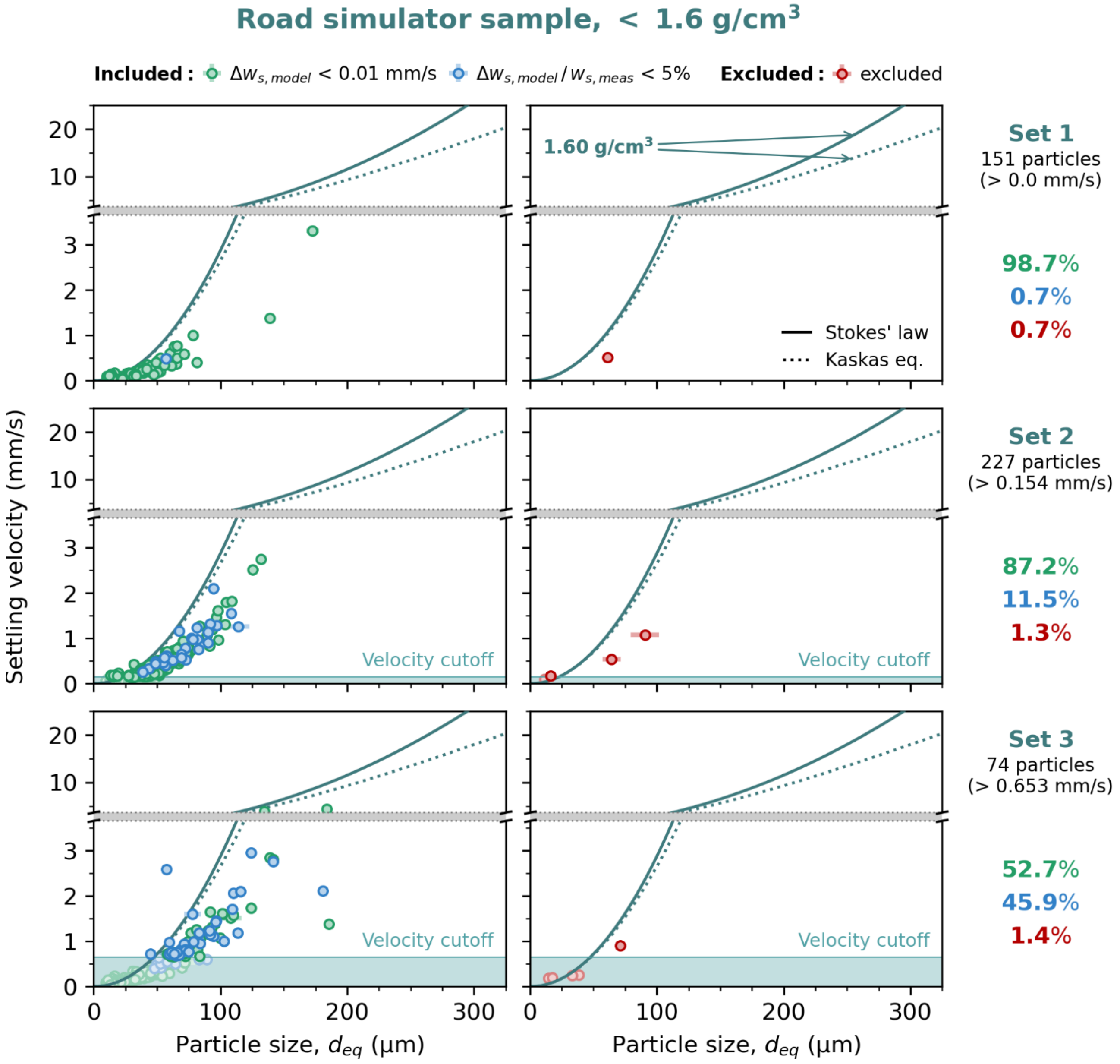

Figure S32. Settling velocities versus particle size as measured for the < 1.6 g/cm³ density fraction of the Road simulator sample. Sets of experiments (rows) as well as included (left column) and excluded particles (right column) according to the empirical model for particle-particle interactions are shown separately. Percentages of particles included with respect to the absolute (green) or relative (blue) criteria for modeled velocity deviation or excluded not meeting both of them (red) are indicated on the right-hand side. Particles with a corrected settling velocity below the respective velocity cutoff are depicted pale and not counted.

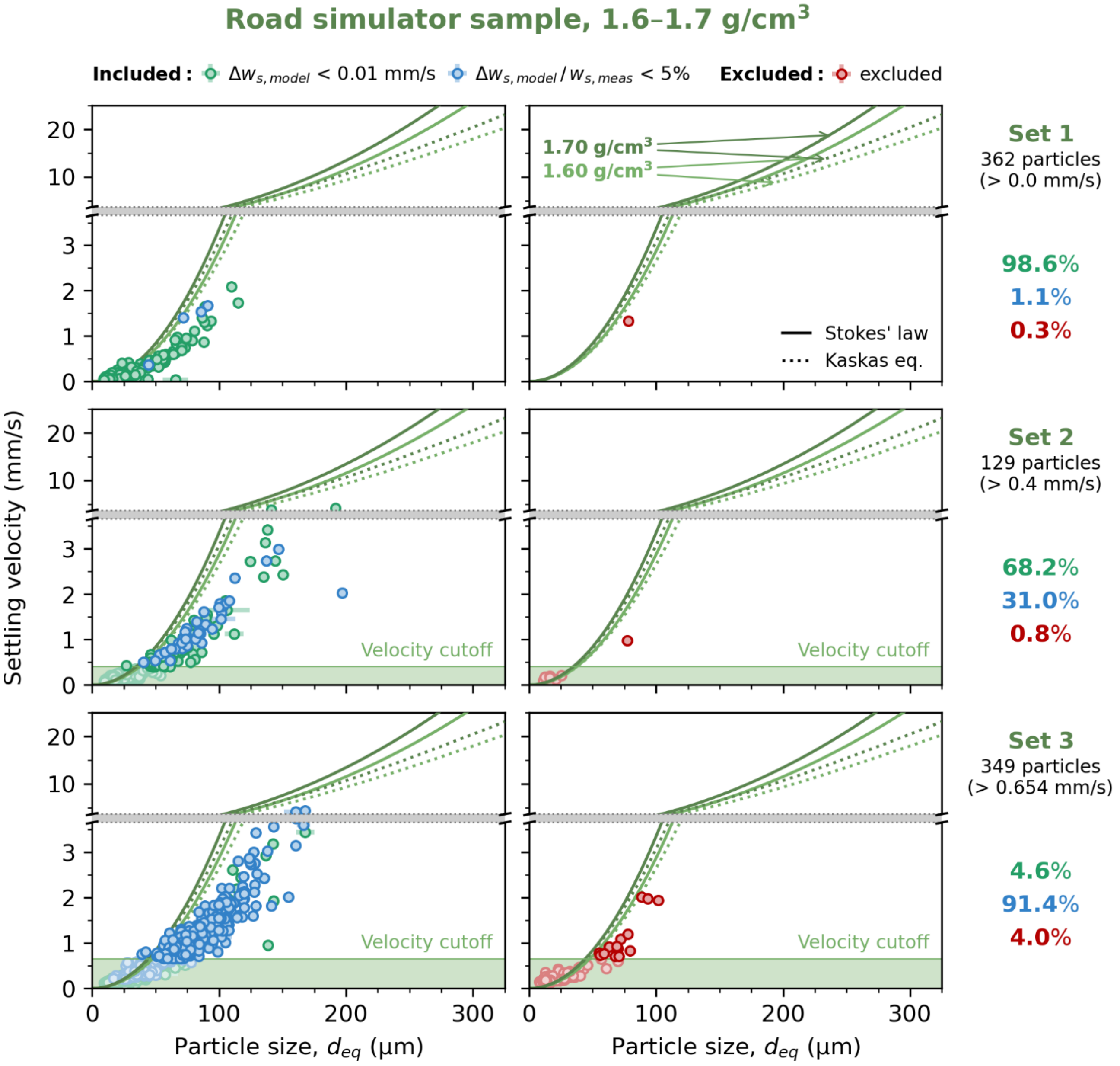

Figure S33. Settling velocities versus particle size as measured for the 1.6–1.7 g/cm³ density fraction of the Road simulator sample. Sets of experiments (rows) as well as included (left column) and excluded particles (right column) according to the empirical model for particle-particle interactions are shown separately. Percentages of particles included with respect to the absolute (green) or relative (blue) criteria for modeled velocity deviation or excluded not meeting both of them (red) are indicated on the right-hand side. Particles with a corrected settling velocity below the respective velocity cutoff are depicted pale and not counted.

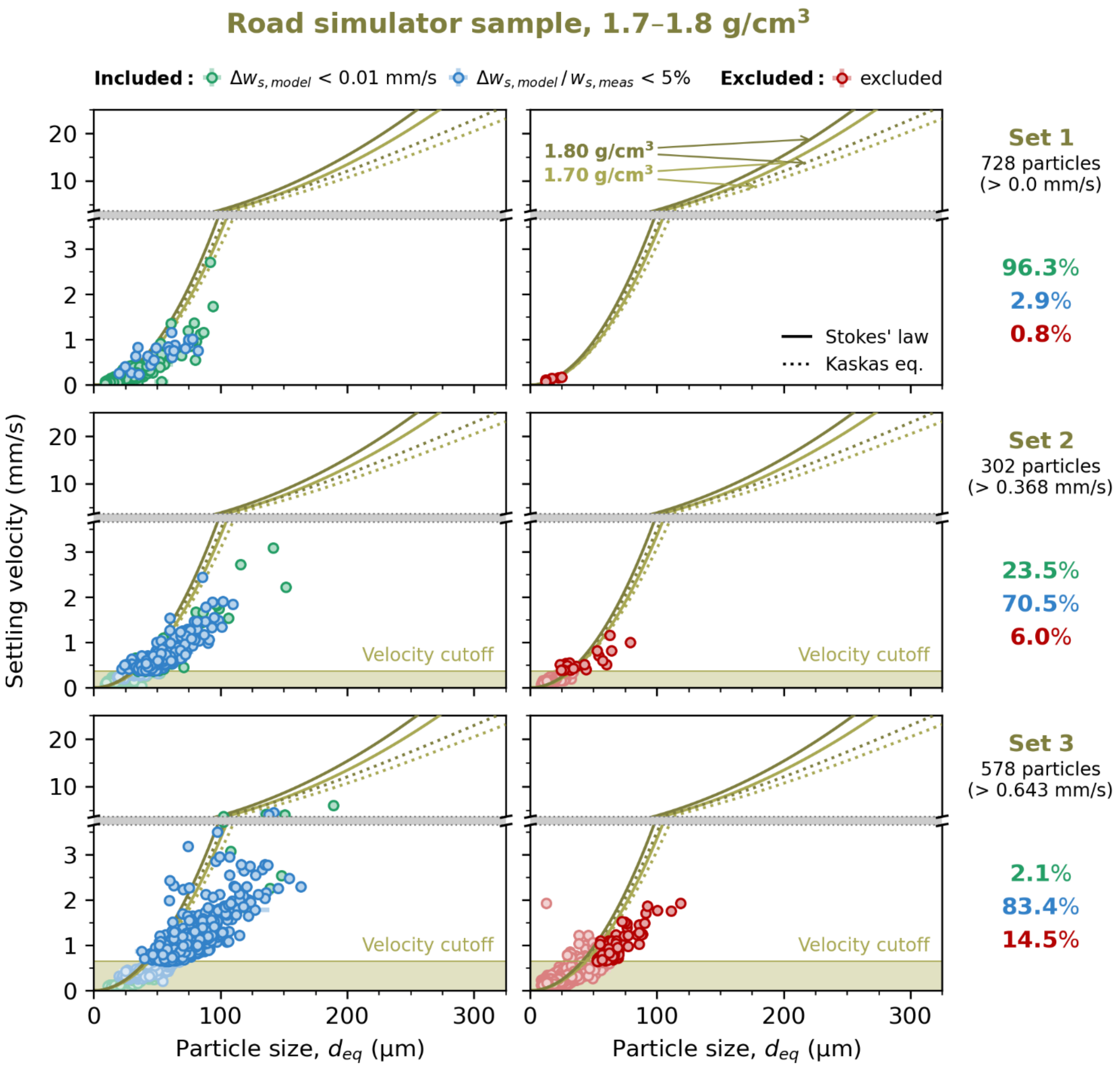

Figure S34. Settling velocities versus particle size as measured for the 1.7–1.8 g/cm³ density fraction of the Road simulator sample. Sets of experiments (rows) as well as included (left column) and excluded particles (right column) according to the empirical model for particle-particle interactions are shown separately. Percentages of particles included with respect to the absolute (green) or relative (blue) criteria for modeled velocity deviation or excluded not meeting both of them (red) are indicated on the right-hand side. Particles with a corrected settling velocity below the respective velocity cutoff are depicted pale and not counted.

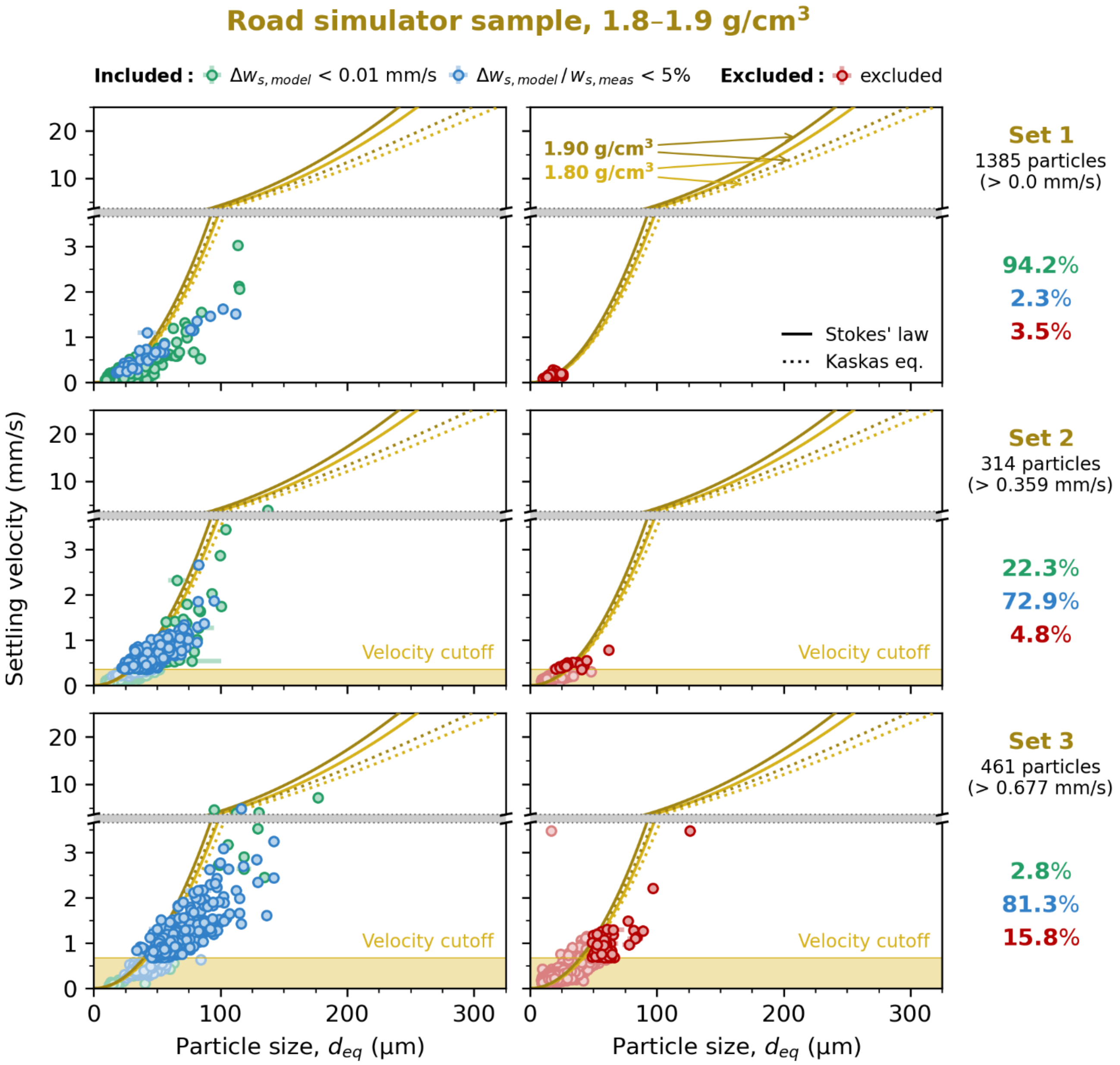

Figure S35. Settling velocities versus particle size as measured for the 1.8–1.9 g/cm³ density fraction of the Road simulator sample. Sets of experiments (rows) as well as included (left column) and excluded particles (right column) according to the empirical model for particle-particle interactions are shown separately. Percentages of particles included with respect to the absolute (green) or relative (blue) criteria for modeled velocity deviation or excluded not meeting both of them (red) are indicated on the right-hand side. Particles with a corrected settling velocity below the respective velocity cutoff are depicted pale and not counted.

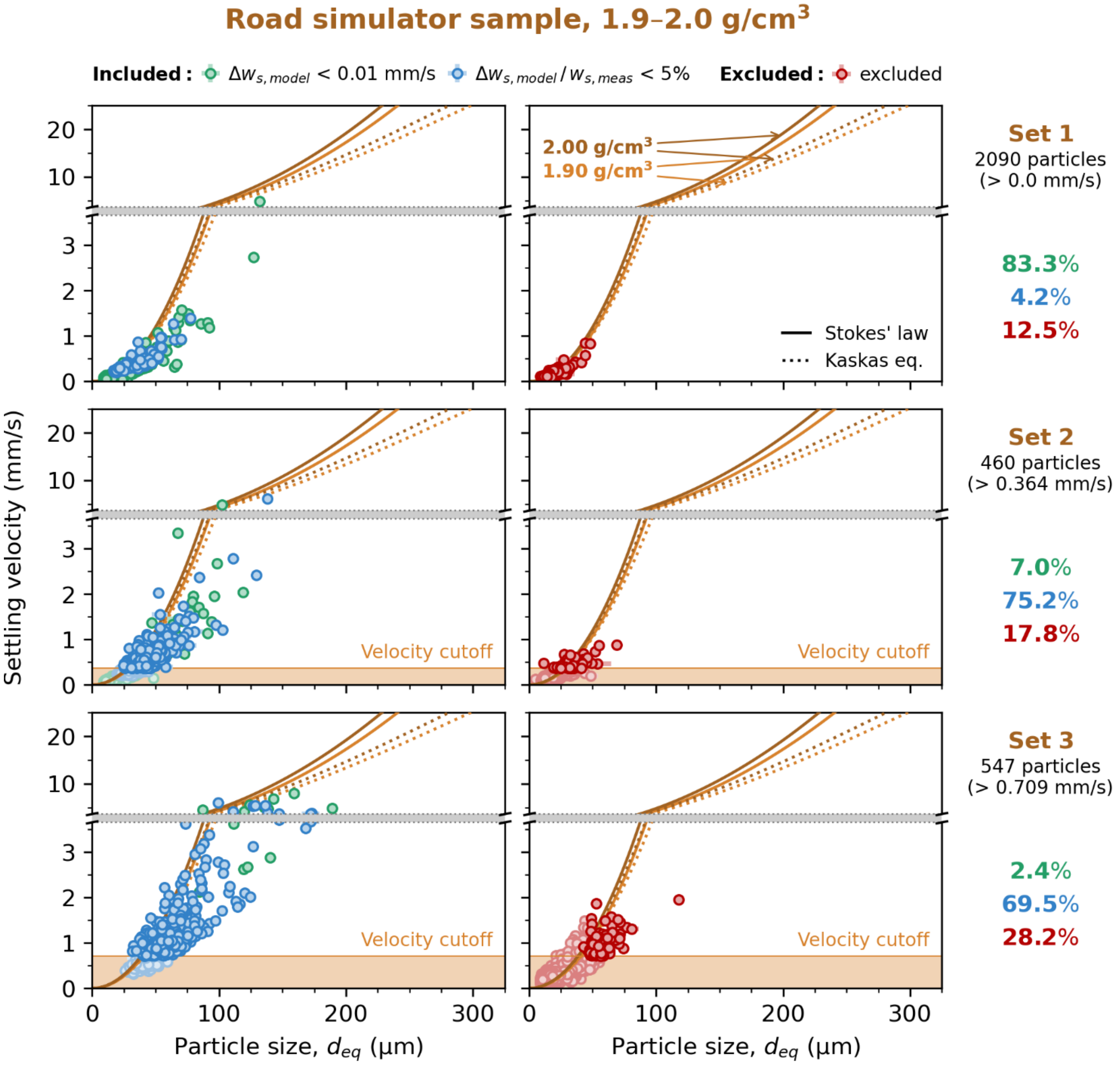

Figure S36. Settling velocities versus particle size as measured for the 1.9–2.0 g/cm³ density fraction of the Road simulator sample. Sets of experiments (rows) as well as included (left column) and excluded particles (right column) according to the empirical model for particle-particle interactions are shown separately. Percentages of particles included with respect to the absolute (green) or relative (blue) criteria for modeled velocity deviation or excluded not meeting both of them (red) are indicated on the right-hand side. Particles with a corrected settling velocity below the respective velocity cutoff are depicted pale and not counted.

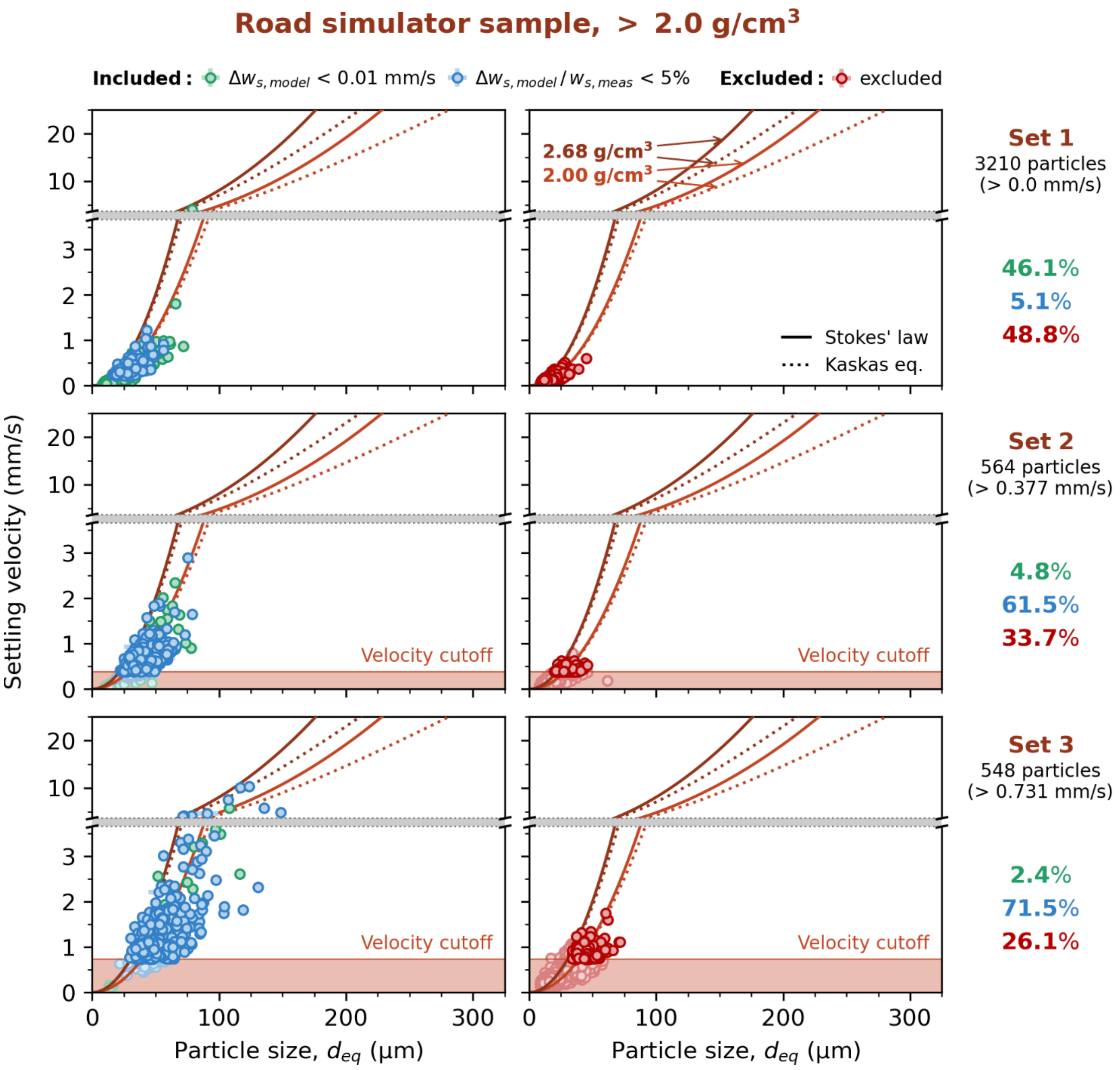

Figure S37. Settling velocities versus particle size as measured for the > 2.0 g/cm³ density fraction of the Road simulator sample. Sets of experiments (rows) as well as included (left column) and excluded particles (right column) according to the empirical model for particle-particle interactions are shown separately. Percentages of particles included with respect to the absolute (green) or relative (blue) criteria for modeled velocity deviation or excluded not meeting both of them (red) are indicated on the right-hand side. Particles with a corrected settling velocity below the respective velocity cutoff are depicted pale and not counted.

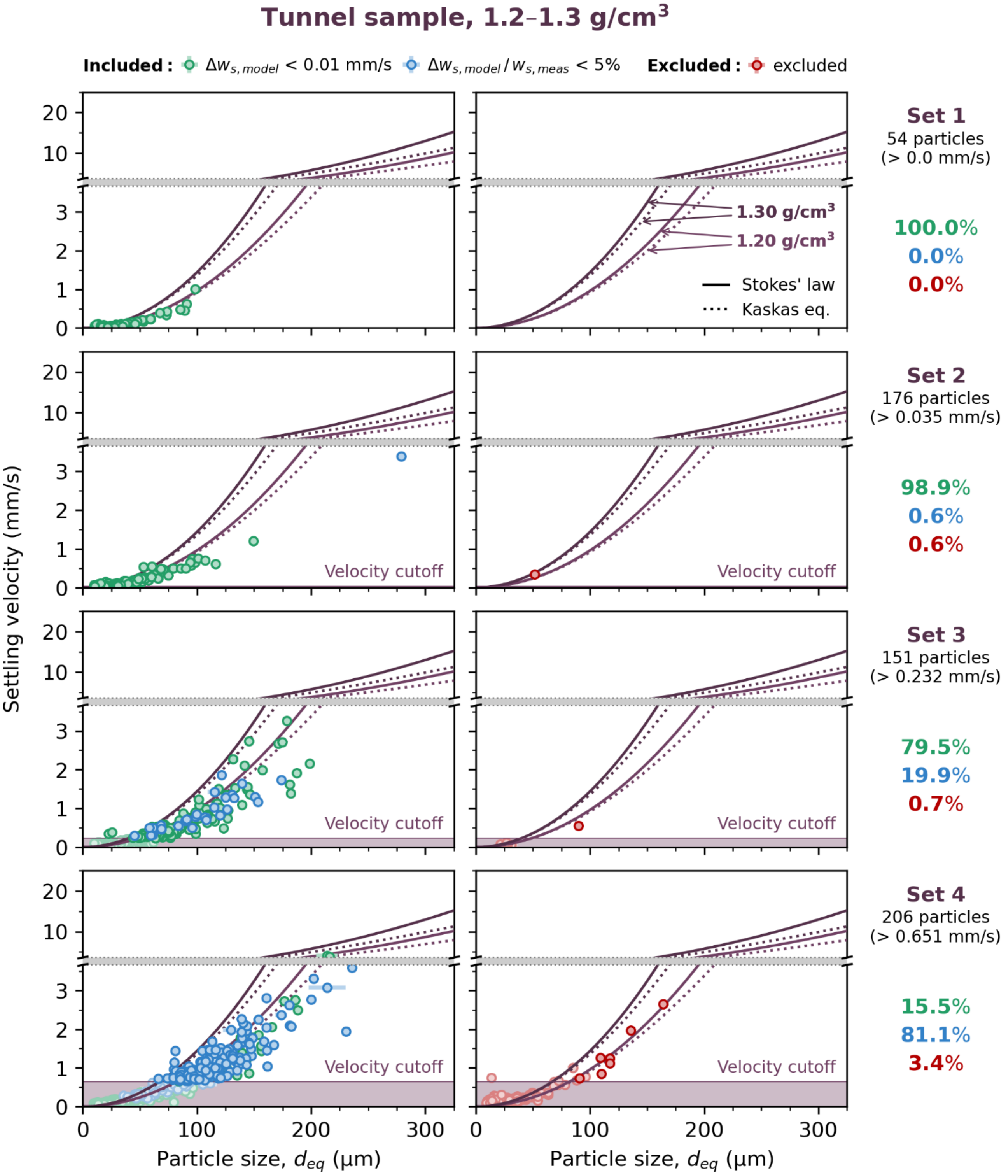

Figure S38. Settling velocities versus particle size as measured for the 1.2–1.3 g/cm³ density fraction of the Tunnel sample. Sets of experiments (rows) as well as included (left column) and excluded particles (right column) according to the empirical model for particle-particle interactions are shown separately. Percentages of particles included with respect to the absolute (green) or relative (blue) criteria for modeled velocity deviation or excluded not meeting both of them (red) are indicated on the right-hand side. Particles with a corrected settling velocity below the respective velocity cutoff are depicted pale and not counted.

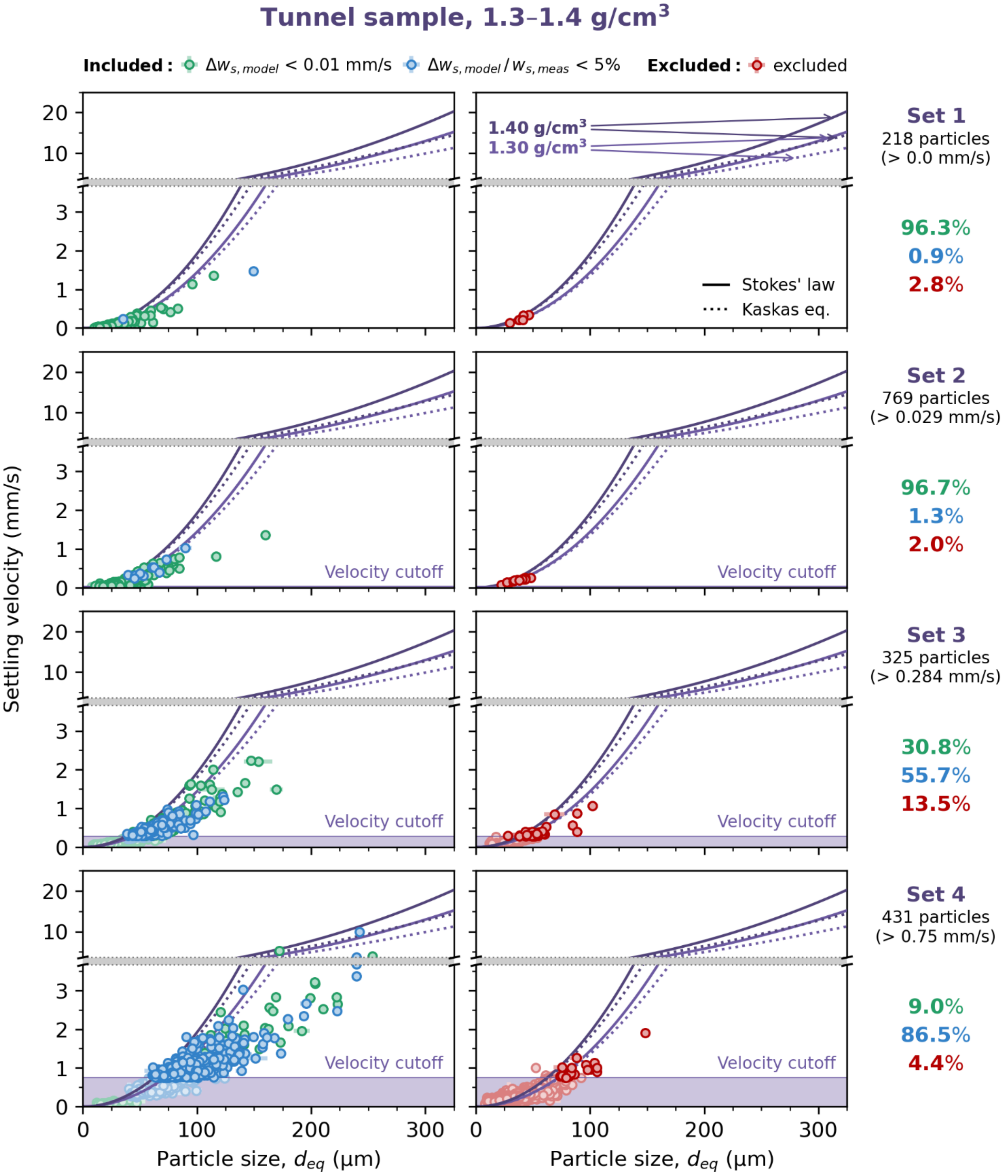

Figure S39. Settling velocities versus particle size as measured for the 1.3–1.4 g/cm³ density fraction of the Tunnel sample. Sets of experiments (rows) as well as included (left column) and excluded particles (right column) according to the empirical model for particle-particle interactions are shown separately. Percentages of particles included with respect to the absolute (green) or relative (blue) criteria for modeled velocity deviation or excluded not meeting both of them (red) are indicated on the right-hand side. Particles with a corrected settling velocity below the respective velocity cutoff are depicted pale and not counted.

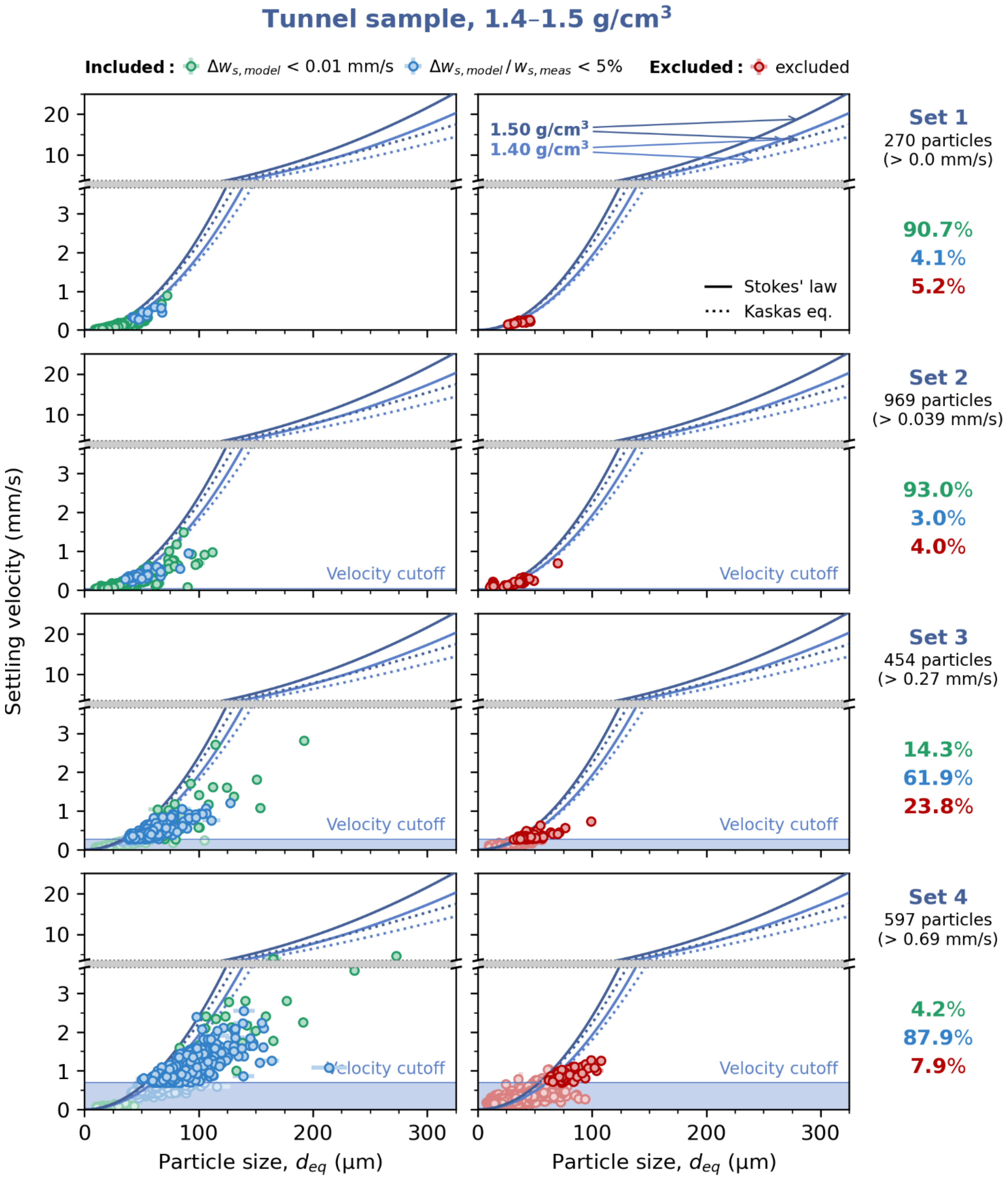

Figure S40. Settling velocities versus particle size as measured for the 1.4–1.5 g/cm³ density fraction of the Tunnel sample. Sets of experiments (rows) as well as included (left column) and excluded particles (right column) according to the empirical model for particle-particle interactions are shown separately. Percentages of particles included with respect to the absolute (green) or relative (blue) criteria for modeled velocity deviation or excluded not meeting both of them (red) are indicated on the right-hand side. Particles with a corrected settling velocity below the respective velocity cutoff are depicted pale and not counted.

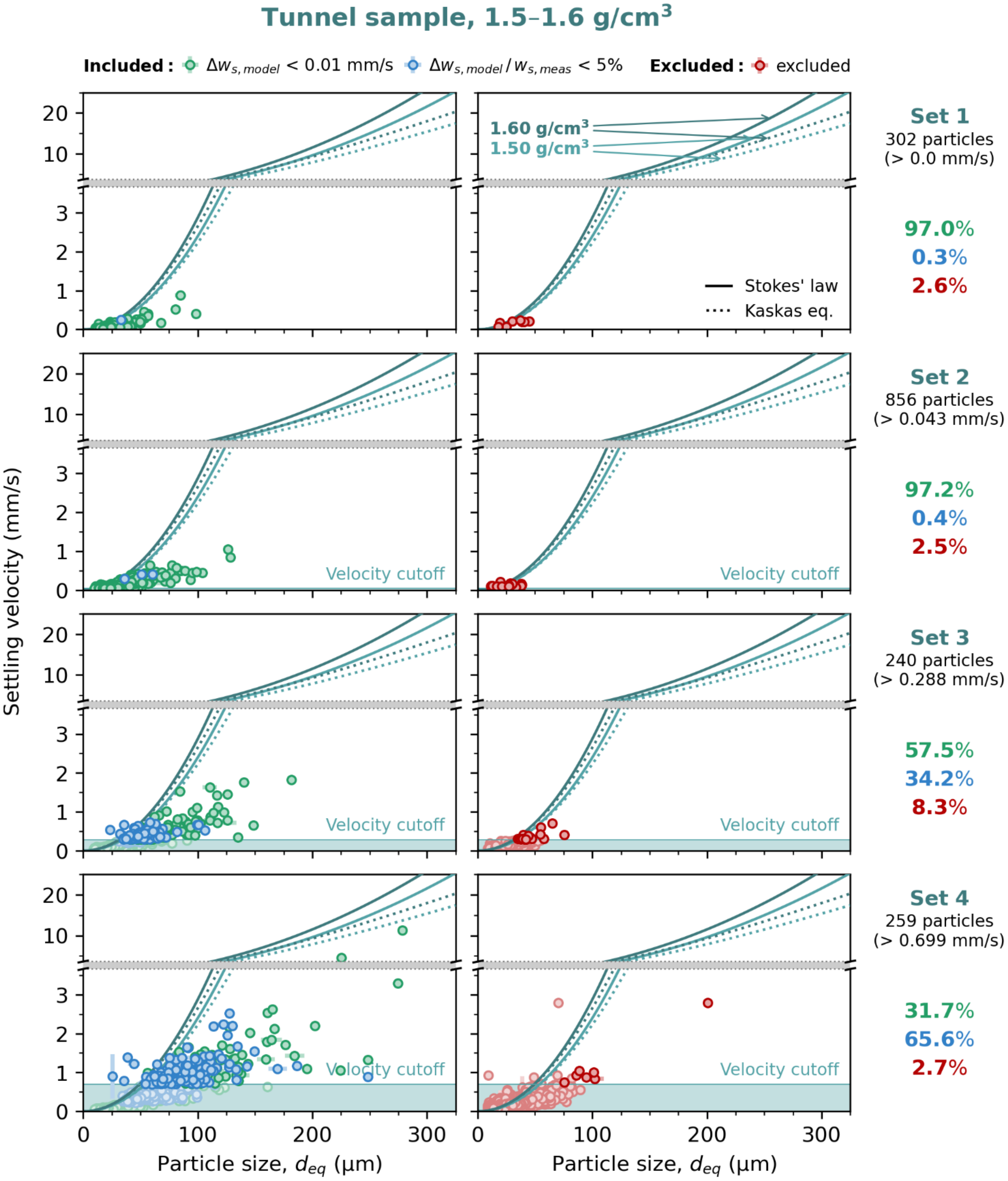

Figure S41. Settling velocities versus particle size as measured for the 1.5–1.6 g/cm³ density fraction of the Tunnel sample. Sets of experiments (rows) as well as included (left column) and excluded particles (right column) according to the empirical model for particle-particle interactions are shown separately. Percentages of particles included with respect to the absolute (green) or relative (blue) criteria for modeled velocity deviation or excluded not meeting both of them (red) are indicated on the right-hand side. Particles with a corrected settling velocity below the respective velocity cutoff are depicted pale and not counted.

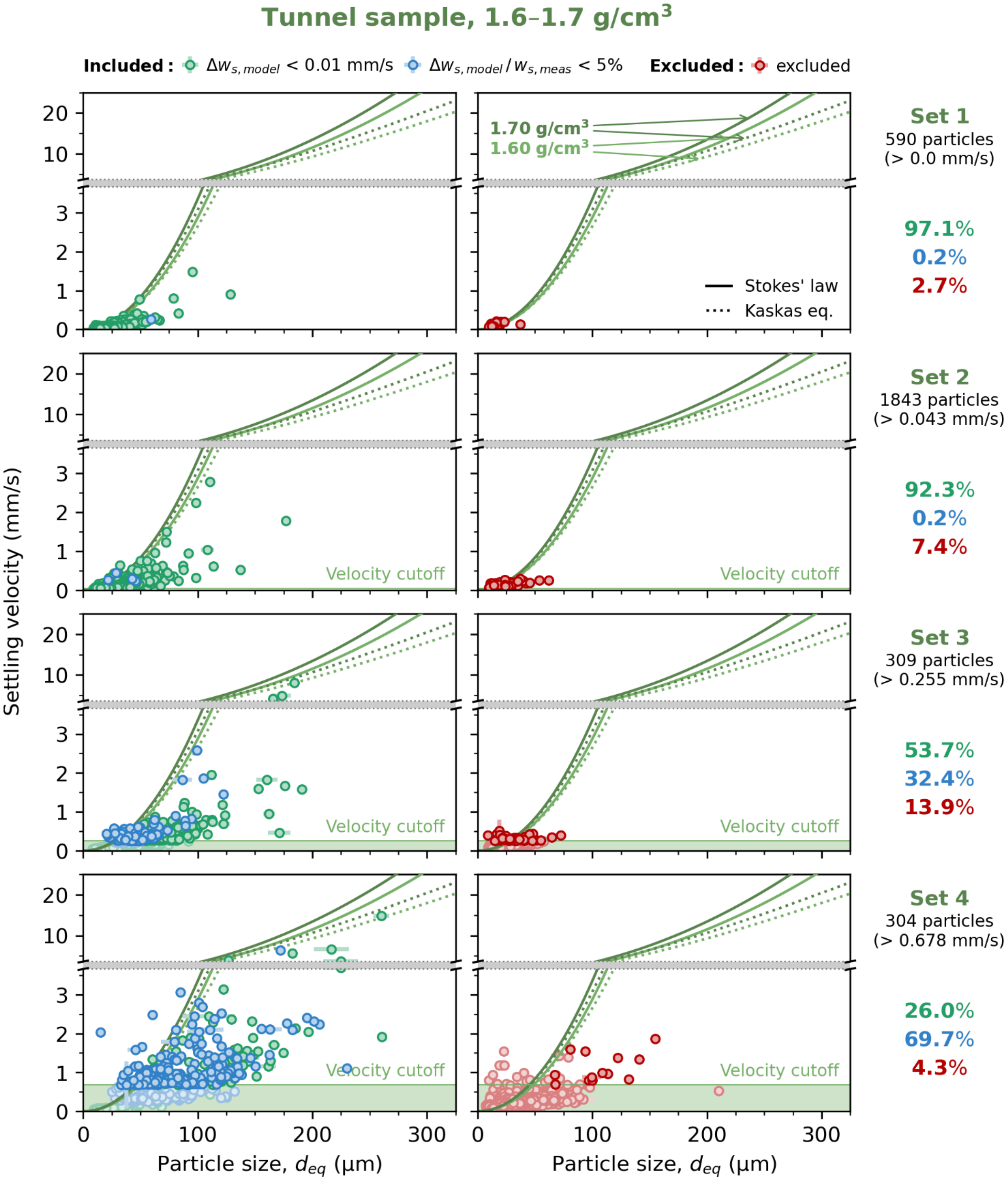

Figure S42. Settling velocities versus particle size as measured for the 1.6–1.7 g/cm³ density fraction of the Tunnel sample. Sets of experiments (rows) as well as included (left column) and excluded particles (right column) according to the empirical model for particle-particle interactions are shown separately. Percentages of particles included with respect to the absolute (green) or relative (blue) criteria for modeled velocity deviation or excluded not meeting both of them (red) are indicated on the right-hand side. Particles with a corrected settling velocity below the respective velocity cutoff are depicted pale and not counted.

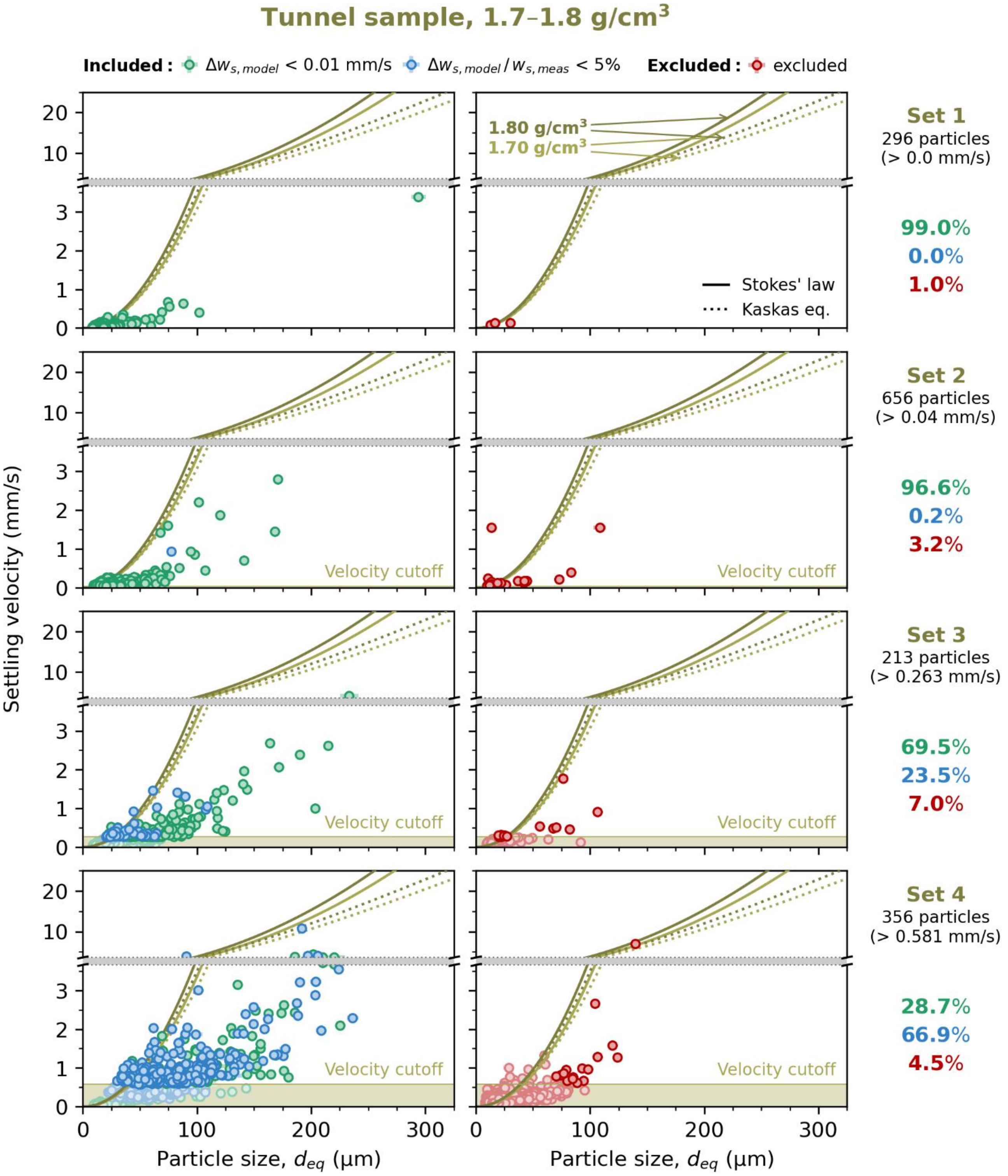

Figure S43. Settling velocities versus particle size as measured for the 1.7–1.8 g/cm³ density fraction of the Tunnel sample. Sets of experiments (rows) as well as included (left column) and excluded particles (right column) according to the empirical model for particle-particle interactions are shown separately. Percentages of particles included with respect to the absolute (green) or relative (blue) criteria for modeled velocity deviation or excluded not meeting both of them (red) are indicated on the right-hand side. Particles with a corrected settling velocity below the respective velocity cutoff are depicted pale and not counted.

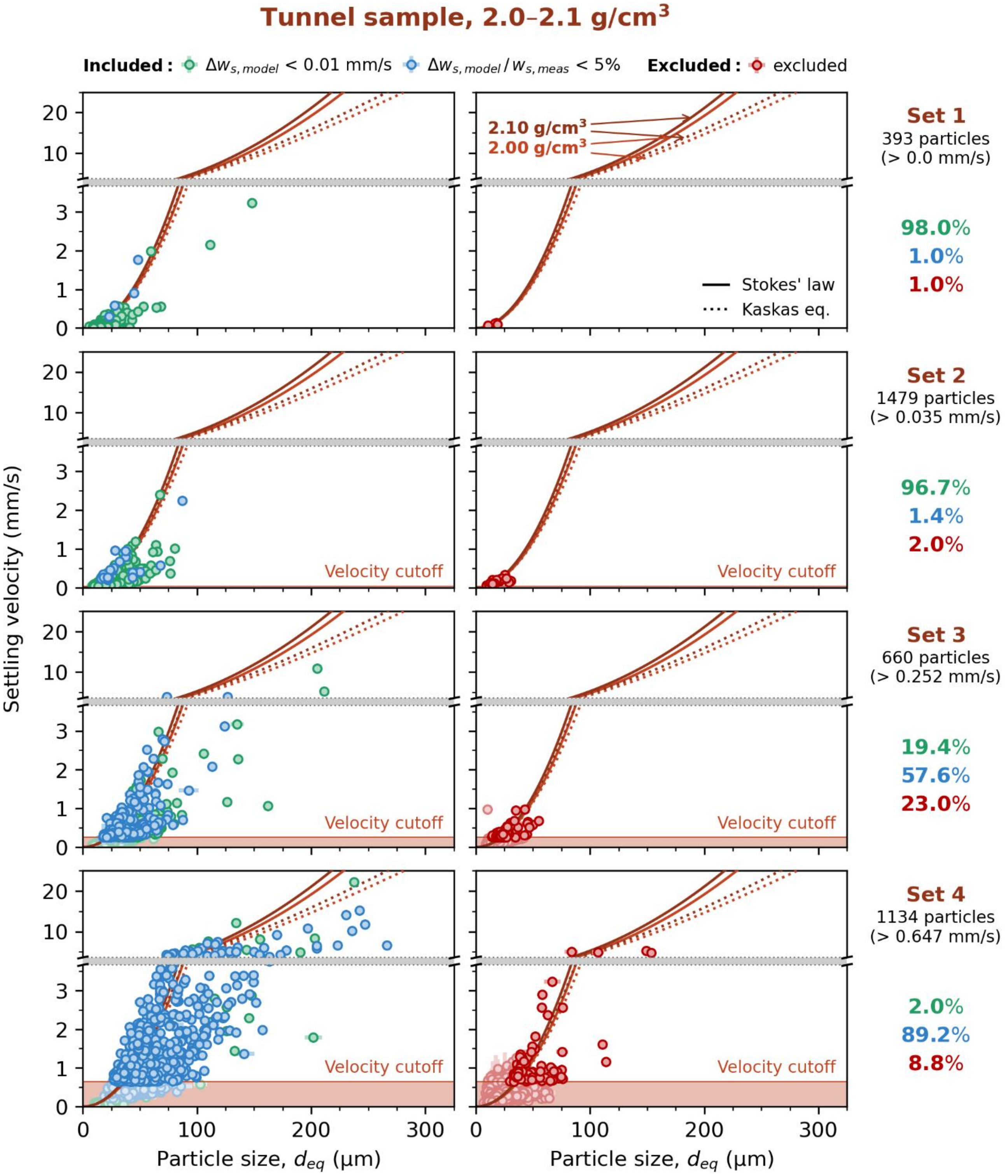

Figure S44. Settling velocities versus particle size as measured for the 2.0–2.1 g/cm³ density fraction of the Tunnel sample. Sets of experiments (rows) as well as included (left column) and excluded particles (right column) according to the empirical model for particle-particle interactions are shown separately. Percentages of particles included with respect to the absolute (green) or relative (blue) criteria for modeled velocity deviation or excluded not meeting both of them (red) are indicated on the right-hand side. Particles with a corrected settling velocity below the respective velocity cutoff are depicted pale and not counted.

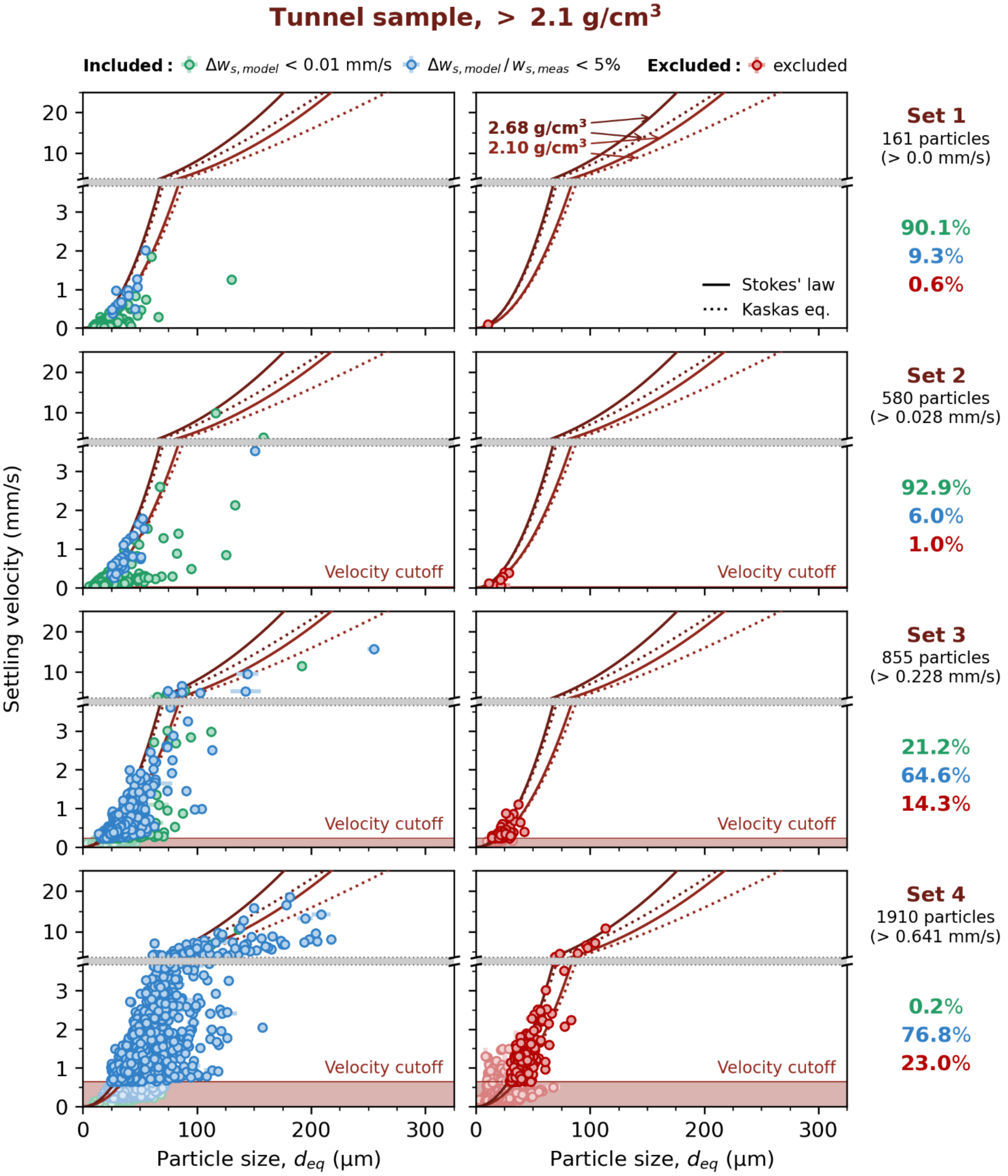

Figure S45. Settling velocities versus particle size as measured for the > 2.0 g/cm³ density fraction of the Tunnel sample. Sets of experiments (rows) as well as included (left column) and excluded particles (right column) according to the empirical model for particle-particle interactions are shown separately. Percentages of particles included with respect to the absolute (green) or relative (blue) criteria for modeled velocity deviation or excluded not meeting both of them (red) are indicated on the right-hand side. Particles with a corrected settling velocity below the respective velocity cutoff are depicted pale and not counted.

S10 Measured particle size and settling velocity distributions

The following figures depict the measured distributions of particle size ($d_{eq,Bagheri}$), equivalent circular diameter (ECD) and settling velocity as measured in settling experiments for each investigated density fraction of the Road simulator sample (Figures S46–S51) and the Tunnel sample (Figures S52–S59). Cumulative distributions related to particle count, estimated particle surface area and estimated particle volume are shown, respectively.

All distributions were derived from tracking data of individual particles according to the procedure described in section S3. Respective individual cutoffs for size and settling velocity in order to combine the data from several sets of experimental runs with different runtime are indicated in each figure. For each depicted distribution, the best fit continuous distribution function from the Python package *scipy.stats*[7] is annotated together with its fitted parameters (cf. section S3 for details on the fitting procedure). The underlying data of all figures, including 95% CI and fitted cumulative distribution functions, is provided in the associated Zenodo repository[12].

Additionally, Tables S5–S7 comprise the quartiles of the cumulative distributions of settling velocity and particle size measured for each investigated density fraction of both samples – either with respect to estimated particle volume (Table S5), estimated particle surface area (Table S6) or particle count (Table S7).

Table S5. Quartiles of cumulative distributions of particle size and settling velocity with respect to estimated particle volume as measured for density fractions of both samples, respectively.

**Sample name**	**Density fraction** (g/cm³)	**Particle size** (µm) Quartiles			**Settling velocity** (mm/s) Quartiles		
		Q1 (25%)	Q2 (50%)	Q3 (75%)	Q1 (25%)	Q2 (50%)	Q3 (75%)
**Road simulator sample**	< 1.6	56.4	89.1	124.2	0.45	1.00	1.72
	1.6–1.7	62.4	90.5	120.1	0.61	1.27	2.03
	1.7–1.8	44.6	70.0	95.2	0.41	0.91	1.54
	1.8–1.9	30.6	51.0	73.6	0.21	0.59	1.23
	1.9–2.0	25.6	41.4	70.9	0.21	0.48	1.20
	> 2.0	18.4	27.5	43.9	0.16	0.30	0.69
**Tunnel sample (20-250 µm)**	1.2–1.3	73.8	115.3	154.4	0.44	0.95	1.78
	1.3–1.4	38.4	56.1	96.3	0.16	0.36	0.90
	1.4–1.5	34.7	50.7	77.4	0.15	0.32	0.72
	1.5–1.6	33.6	50.8	93.6	0.12	0.25	0.59
	1.6–1.7	29.9	45.2	81.1	0.09	0.20	0.48
	1.7–1.8	37.8	66.3	118.0	0.12	0.28	0.93
	2.0–2.1	27.8	53.0	119.2	0.15	0.53	2.43
	> 2.1	34.7	63.2	122.6	0.32	1.23	4.30

Table S6. Quartiles of cumulative distributions of particle size and settling velocity with respect to estimated particle surface area as measured for density fractions of both samples, respectively.

Sample name	Density fraction (g/cm³)	**Particle size** (µm)			**Settling velocity** (mm/s)		
		**Quartiles**			**Quartiles**		
		Q1 (25%)	Q2 (50%)	Q3 (75%)	Q1 (25%)	Q2 (50%)	Q3 (75%)
**Road simulator sample**	< 1.6	42.0	59.9	91.9	0.24	0.51	1.17
	1.6–1.7	38.7	66.1	99.5	0.27	0.79	1.55
	1.7–1.8	27.5	47.9	76.2	0.18	0.48	1.04
	1.8–1.9	20.1	31.9	52.8	0.13	0.24	0.62
	1.9–2.0	17.9	27.1	42.1	0.13	0.22	0.51
	> 2.0	14.9	20.2	29.8	0.12	0.19	0.35
**Tunnel sample (20-250 µm)**	1.2–1.3	44.1	85.3	127.6	0.16	0.55	1.25
	1.3–1.4	30.2	42.1	62.1	0.11	0.20	0.46
	1.4–1.5	28.3	38.7	55.4	0.10	0.20	0.42
	1.5–1.6	25.5	35.3	51.3	0.08	0.16	0.27
	1.6–1.7	22.8	32.5	48.1	0.06	0.11	0.21
	1.7–1.8	23.5	37.1	64.0	0.05	0.12	0.29
	2.0–2.1	17.9	26.5	45.3	0.06	0.14	0.45
	> 2.1	22.8	32.5	54.4	0.13	0.37	1.20

Table S7. Quartiles of cumulative distributions of particle size and settling velocity with respect to particle count as measured for density fractions of both samples, respectively.

Sample name	Density fraction (g/cm³)	**Particle size** (µm)			**Settling velocity** (mm/s)		
		**Quartiles**			**Quartiles**		
		Q1 (25%)	Q2 (50%)	Q3 (75%)	Q1 (25%)	Q2 (50%)	Q3 (75%)
**Road simulator sample**	< 1.6	14.5	24	41.4	0.06	0.11	0.25
	1.6–1.7	11.5	14.3	24.5	0.07	0.1	0.15
	1.7–1.8	12.6	16.9	25.8	0.07	0.11	0.18
	1.8–1.9	12.1	14.7	21.6	0.07	0.1	0.16
	1.9–2.0	12.5	15.6	20.6	0.08	0.11	0.17
	> 2.0	12.1	14.4	18.3	0.09	0.12	0.18
**Tunnel sample (20-250 µm)**	1.2–1.3	20.2	28	40.2	0.04	0.08	0.15
	1.3–1.4	22.7	28	36.9	0.05	0.09	0.15
	1.4–1.5	17.1	23.8	32.5	0.04	0.08	0.14
	1.5–1.6	17.5	22.5	30.3	0.04	0.07	0.12
	1.6–1.7	14.4	18.7	26.1	0.03	0.05	0.09
	1.7–1.8	13.2	18.6	25.4	0.03	0.04	0.08
	2.0–2.1	12	15.6	21.1	0.04	0.06	0.12
	> 2.1	12.7	17.5	25.5	0.05	0.1	0.25

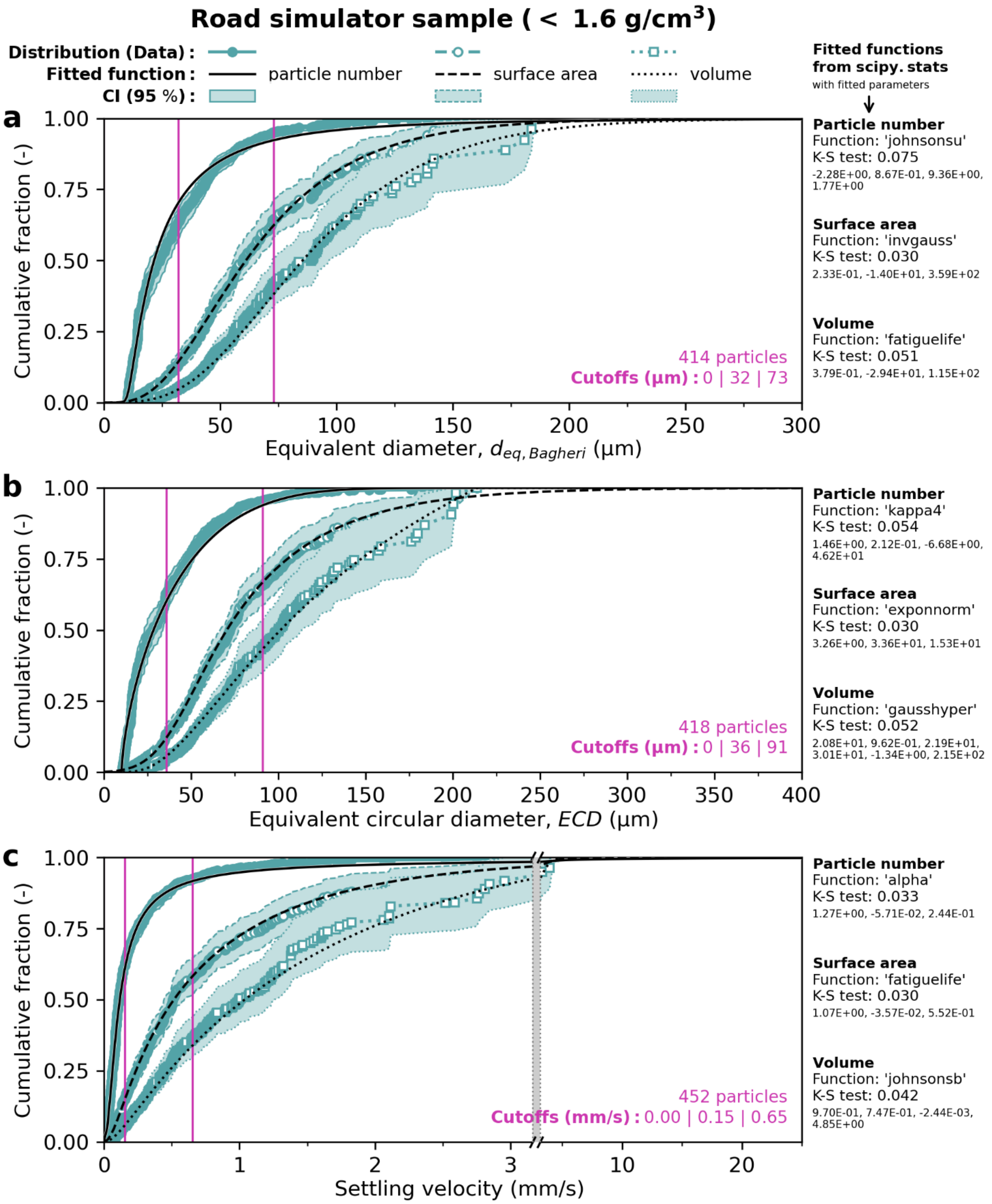

Figure S46. Cumulative distributions of particle size as equivalent diameter (a) or equivalent circular diameter (b) and settling velocity (c) composed from measurements for fraction < 1.6 g/cm³ of the Road simulator sample. Cumulative particle number, surface area and volume shown, respectively, including 95% confidence interval derived via bootstrapping (N=2000) and best fit continuous distribution function from Python package *scipy.stats* (function name[8], K-S test statistic and fitted parameters given on the right-hand side). Note computed cutoffs indicated in pink (cf. section S3).

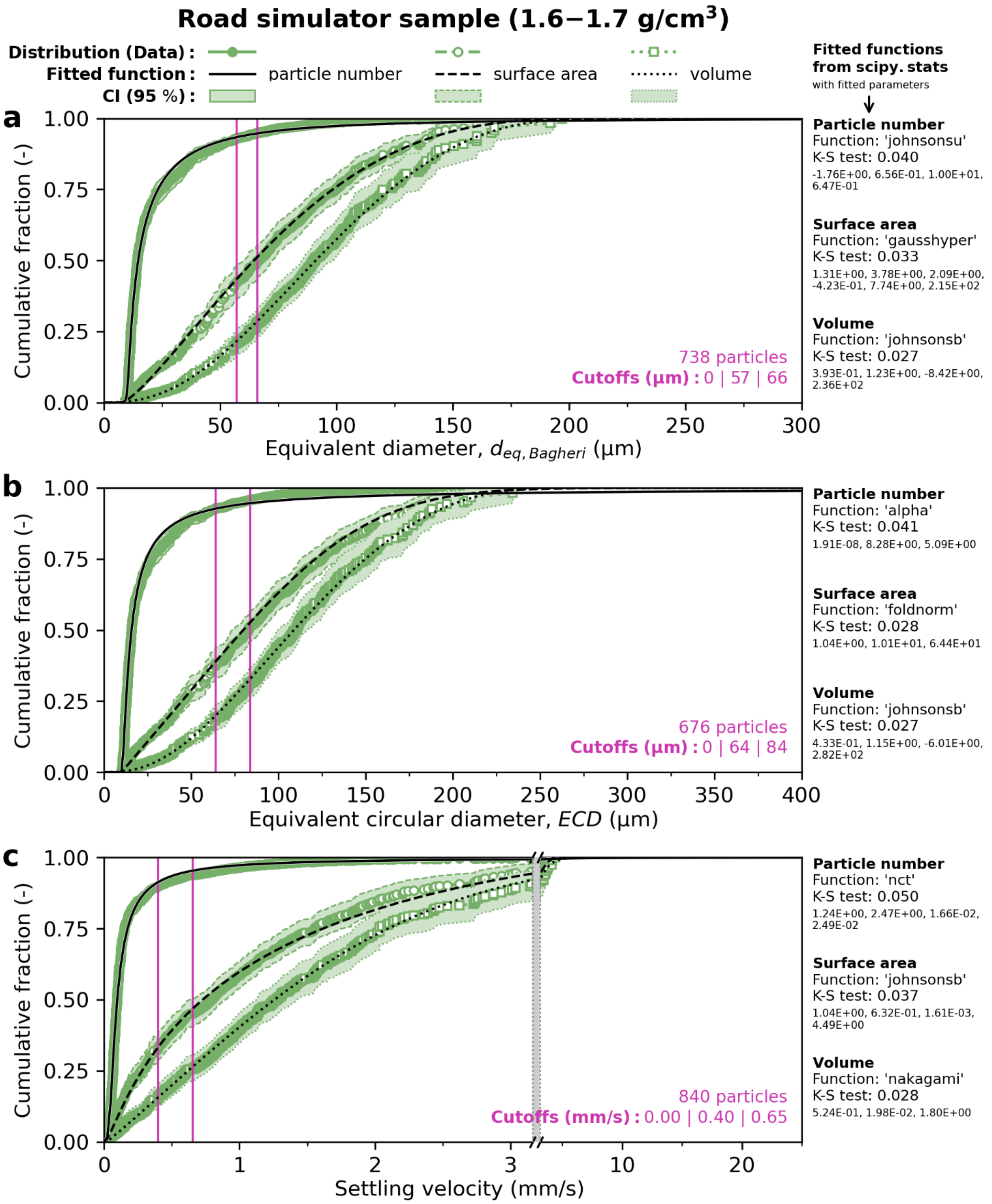

Figure S47. Cumulative distributions of particle size as equivalent diameter (a) or equivalent circular diameter (b) and settling velocity (c) composed from measurements for fraction 1.6–1.7 g/cm³ of the Road simulator sample. Cumulative particle number, surface area and volume shown, respectively, including 95% confidence interval derived via bootstrapping (N=2000) and best fit continuous distribution function from Python package *scipy.stats* (function name[8], K-S test statistic and fitted parameters given on the right-hand side). Note computed cutoffs indicated in pink (cf. section S3).

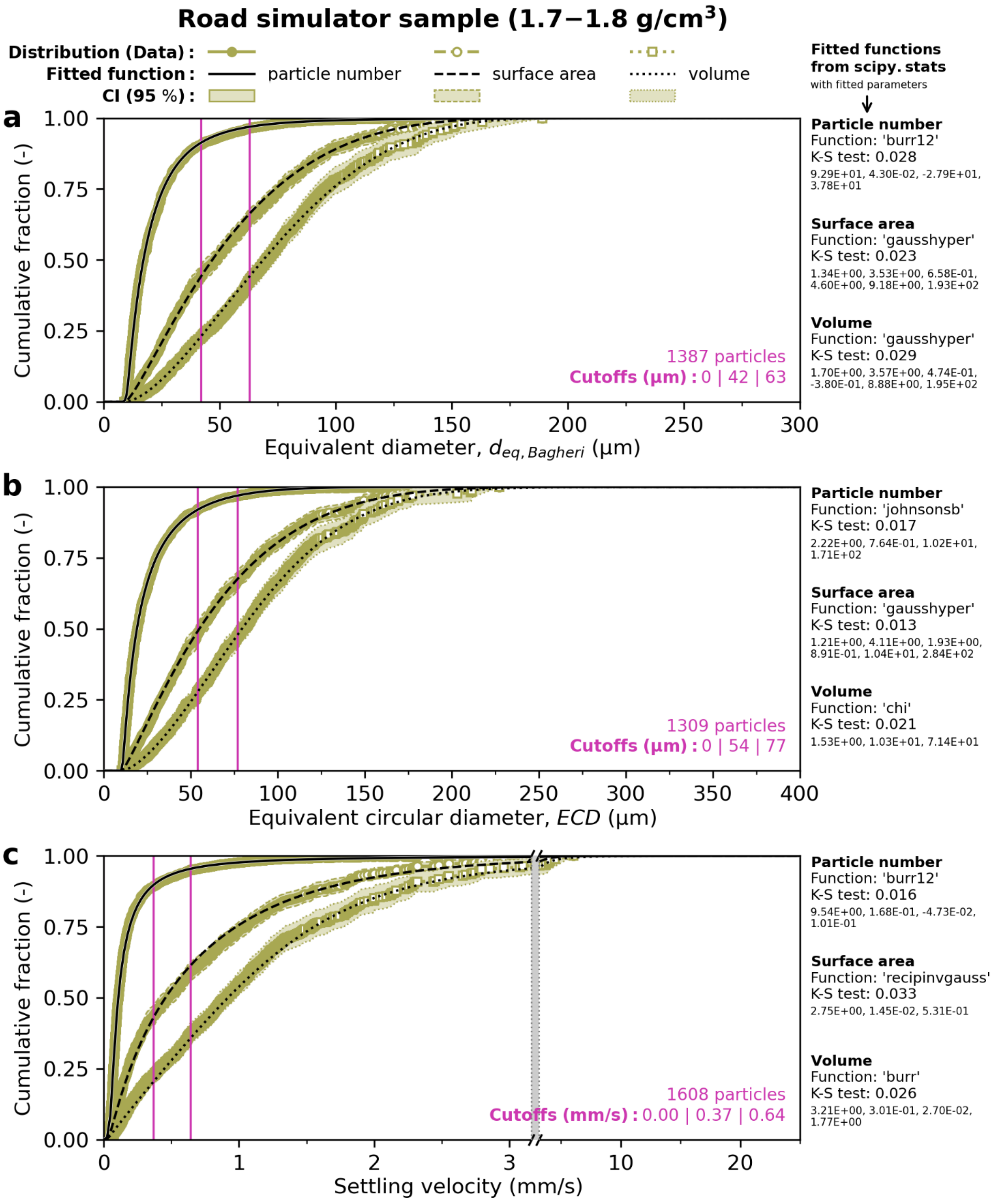

Figure S48. Cumulative distributions of particle size as equivalent diameter (a) or equivalent circular diameter (b) and settling velocity (c) composed from measurements for fraction 1.7–1.8 g/cm³ of the Road simulator sample. Cumulative particle number, surface area and volume shown, respectively, including 95% confidence interval derived via bootstrapping (N=2000) and best fit continuous distribution function from Python package *scipy.stats* (function name[8], K-S test statistic and fitted parameters given on the right-hand side). Note computed cutoffs indicated in pink (cf. section S3).

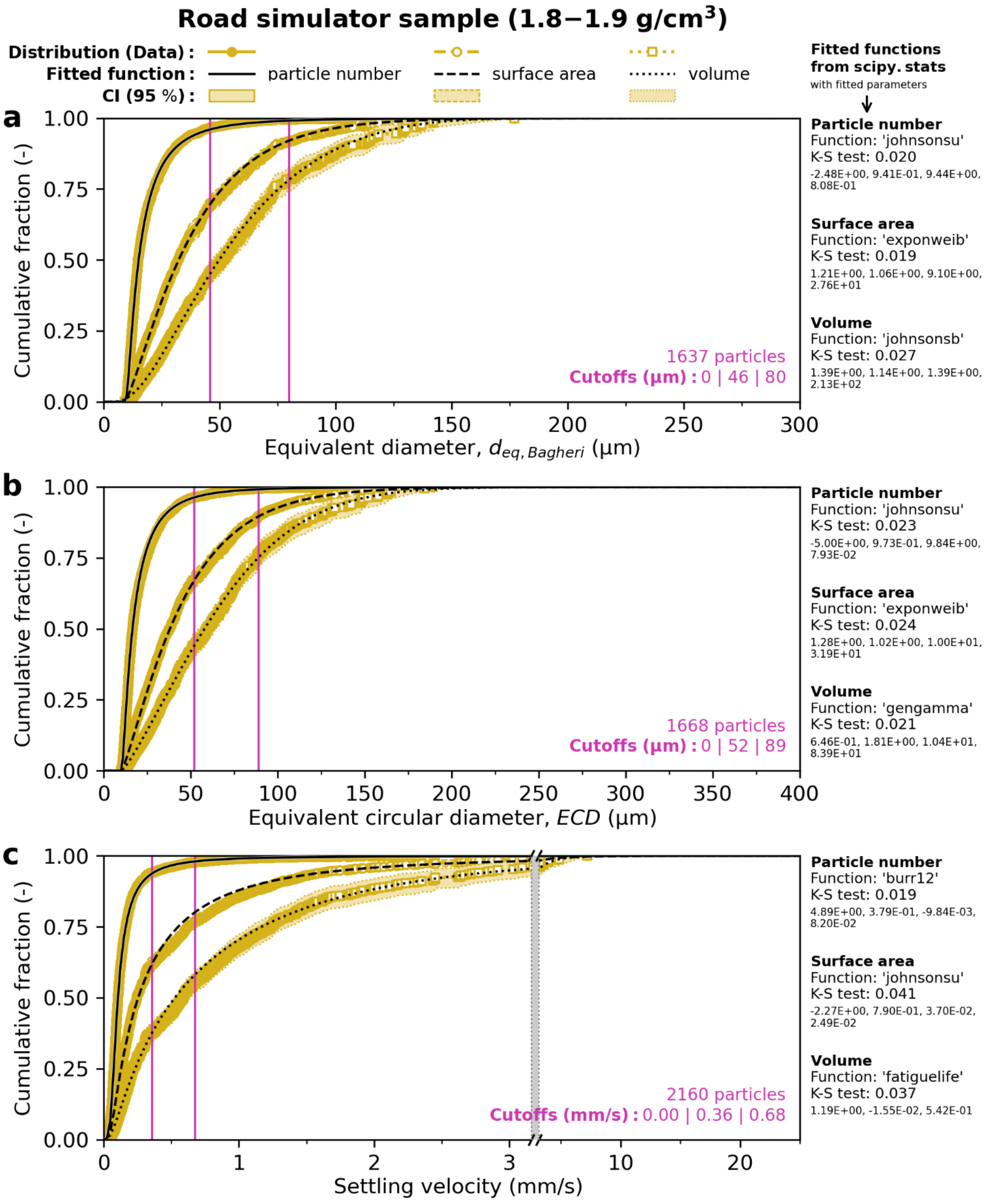

Figure S49. Cumulative distributions of particle size as equivalent diameter (a) or equivalent circular diameter (b) and settling velocity (c) composed from measurements for fraction 1.8–1.9 g/cm³ of the Road simulator sample. Cumulative particle number, surface area and volume shown, respectively, including 95% confidence interval derived via bootstrapping (N=2000) and best fit continuous distribution function from Python package *scipy.stats* (function name[8], K-S test statistic and fitted parameters given on the right-hand side). Note computed cutoffs indicated in pink (cf. section S3).

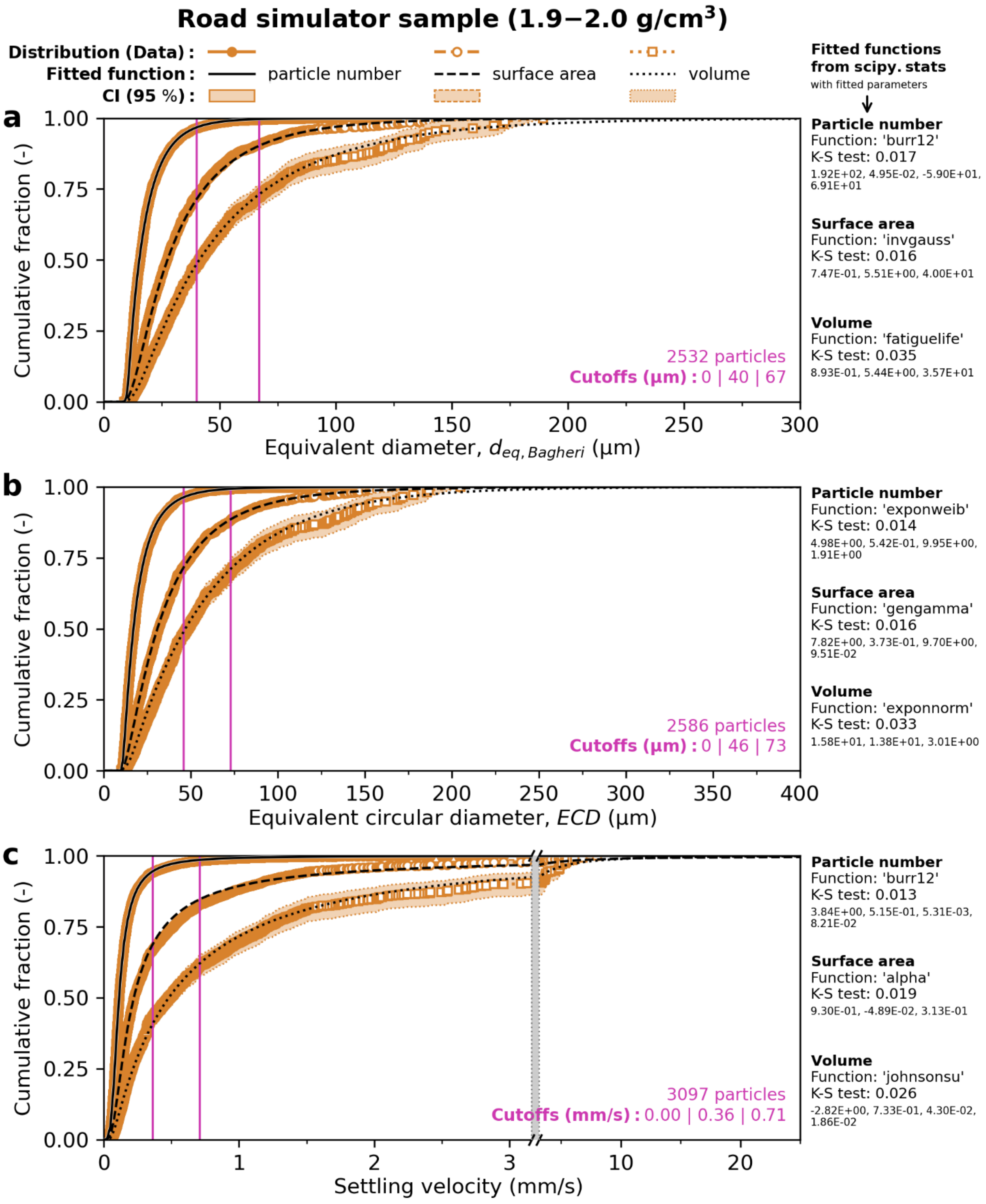

Figure S50. Cumulative distributions of particle size as equivalent diameter (a) or equivalent circular diameter (b) and settling velocity (c) composed from measurements for fraction 1.9–2.0 g/cm³ of the Road simulator sample. Cumulative particle number, surface area and volume shown, respectively, including 95% confidence interval derived via bootstrapping (N=2000) and best fit continuous distribution function from Python package *scipy.stats* (function name[8], K-S test statistic and fitted parameters given on the right-hand side). Note computed cutoffs indicated in pink (cf. section S3).

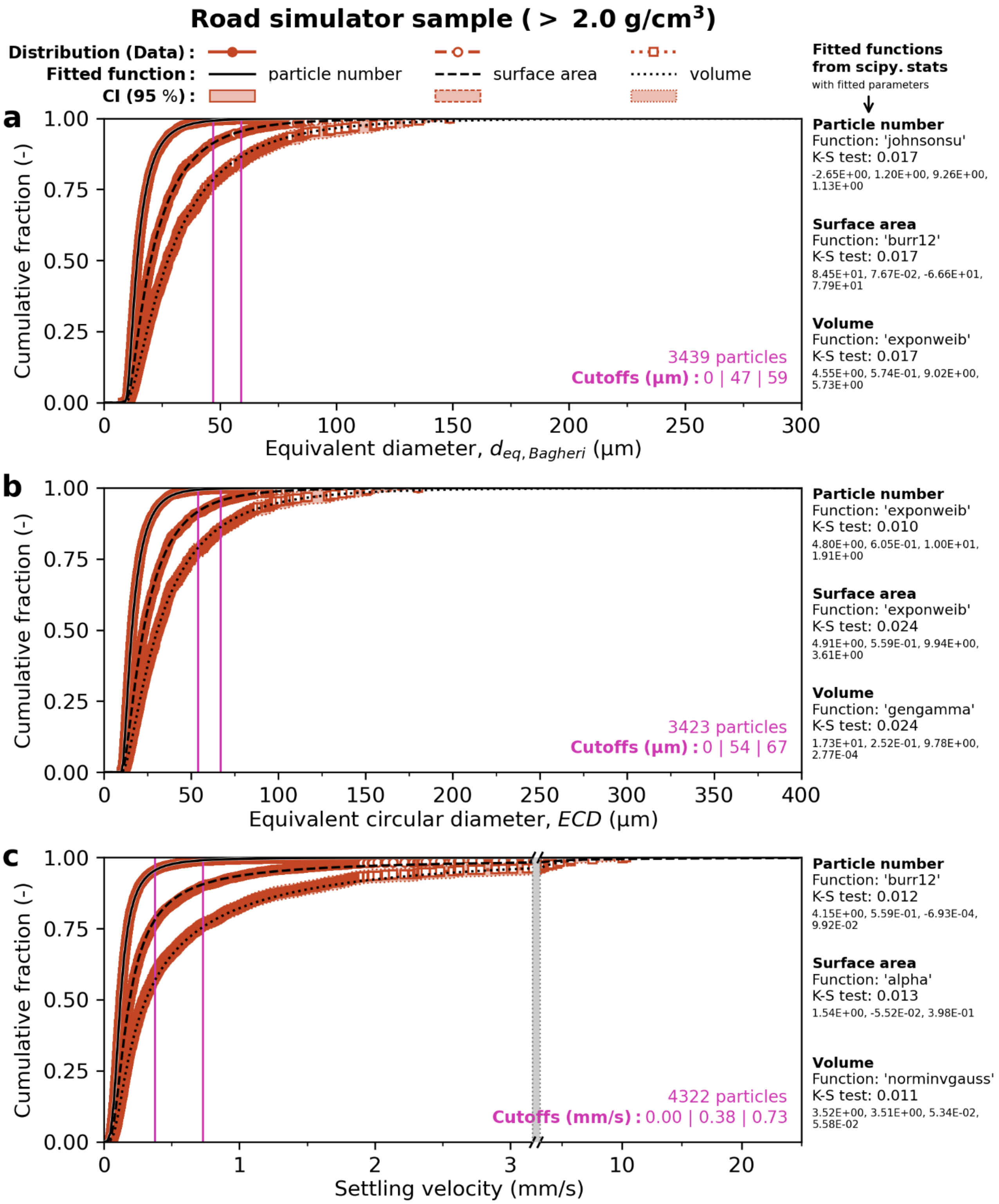

Figure S51. Cumulative distributions of particle size as equivalent diameter (a) or equivalent circular diameter (b) and settling velocity (c) composed from measurements for fraction > 2.0 g/cm³ of the Road simulator sample. Cumulative particle number, surface area and volume shown, respectively, including 95% confidence interval derived via bootstrapping (N=2000) and best fit continuous distribution function from Python package *scipy.stats* (function name[8], K-S test statistic and fitted parameters given on the right-hand side). Note computed cutoffs indicated in pink (cf. section S3).

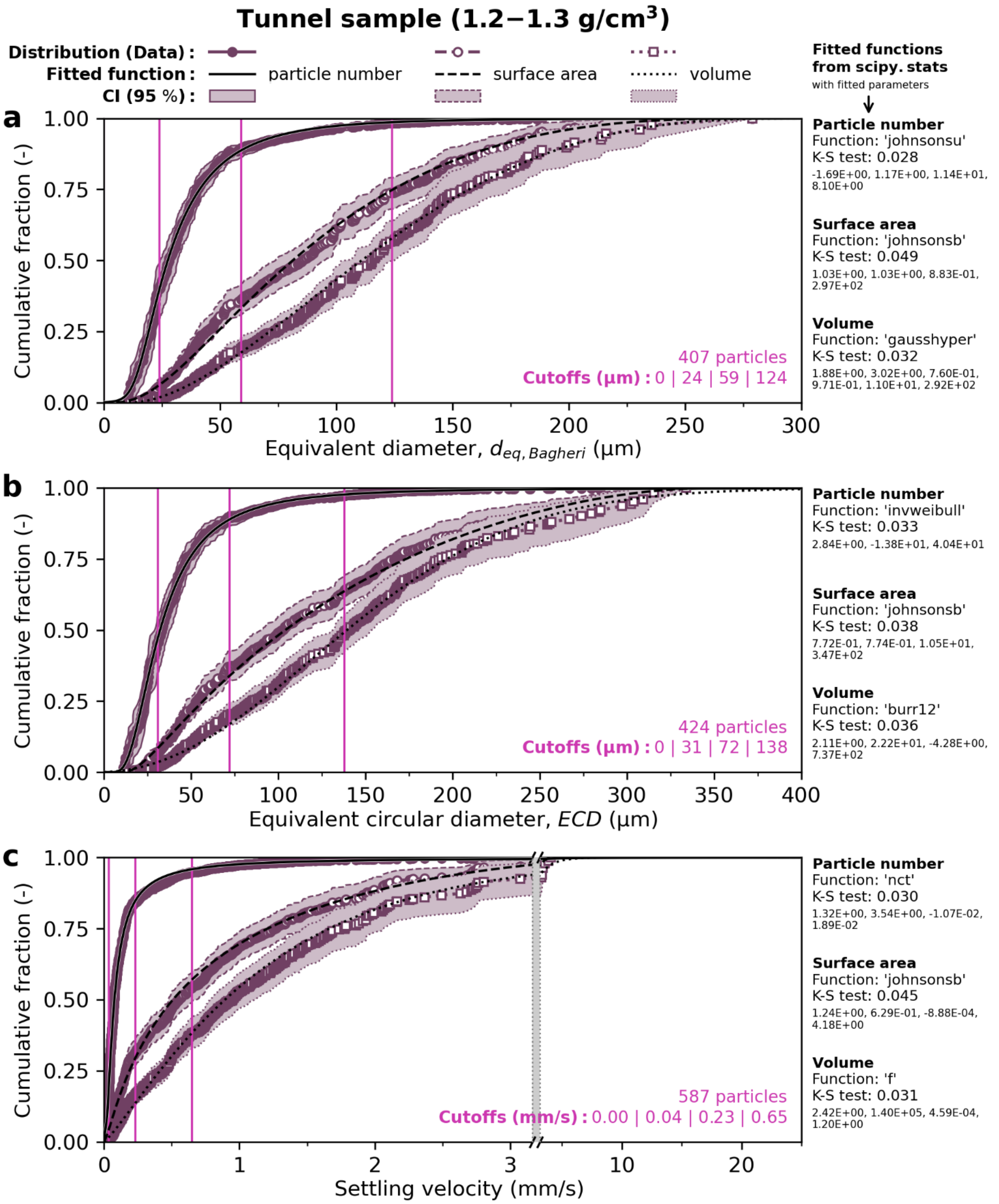

Figure S52. Cumulative distributions of particle size as equivalent diameter (a) or equivalent circular diameter (b) and settling velocity (c) composed from measurements for fraction 1.2–1.3 g/cm³ of the Tunnel sample. Cumulative particle number, surface area and volume shown, respectively, including 95% confidence interval derived via bootstrapping (N=2000) and best fit continuous distribution function from Python package *scipy.stats* (function name[8], K-S test statistic and fitted parameters given on the right-hand side). Note computed cutoffs indicated in pink (cf. section S3).

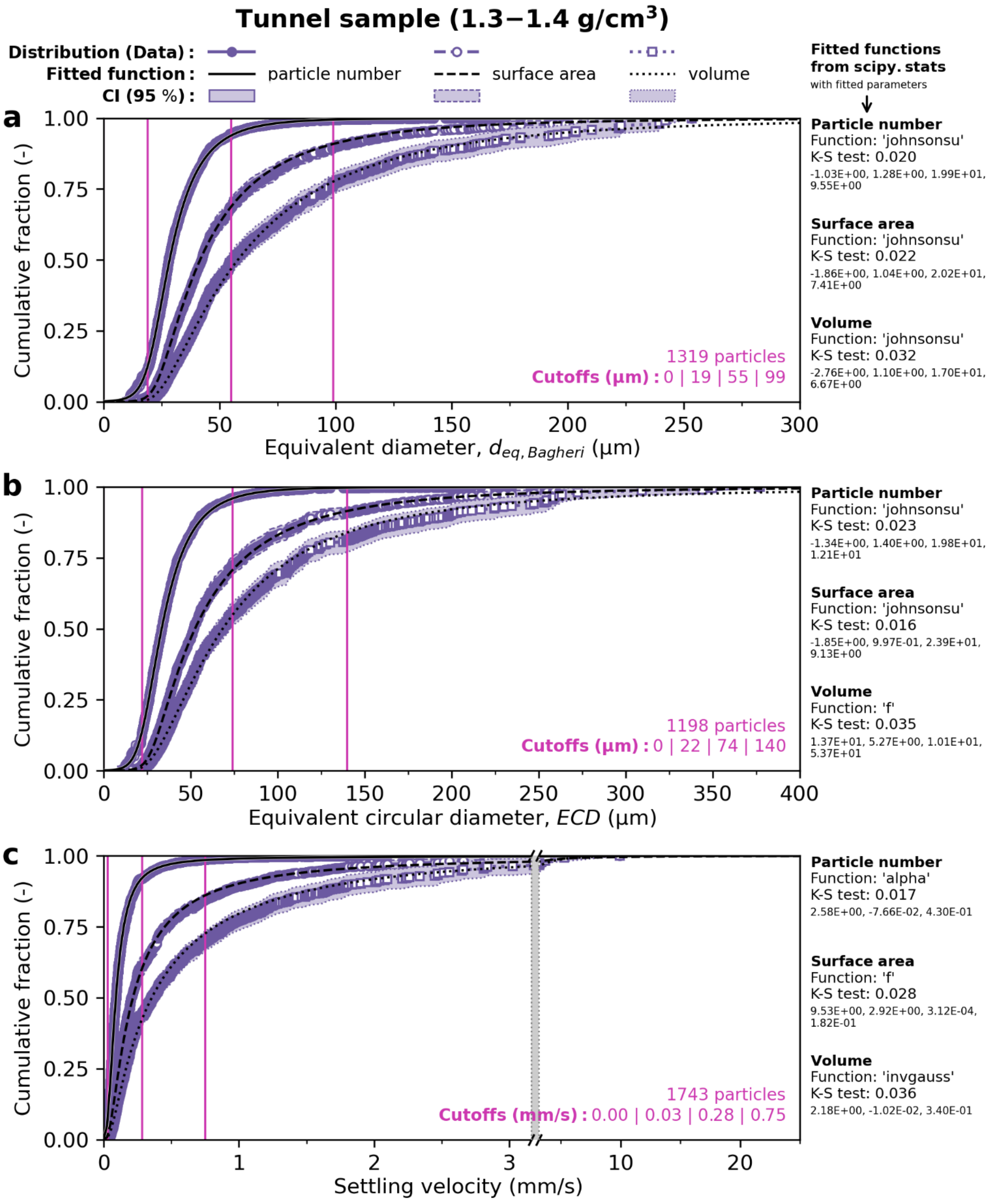

Figure S53. Cumulative distributions of particle size as equivalent diameter (a) or equivalent circular diameter (b) and settling velocity (c) composed from measurements for fraction 1.3–1.4 g/cm³ of the Tunnel sample. Cumulative particle number, surface area and volume shown, respectively, including 95% confidence interval derived via bootstrapping (N=2000) and best fit continuous distribution function from Python package *scipy.stats* (function name[8], K-S test statistic and fitted parameters given on the right-hand side). Note computed cutoffs indicated in pink (cf. section S3).

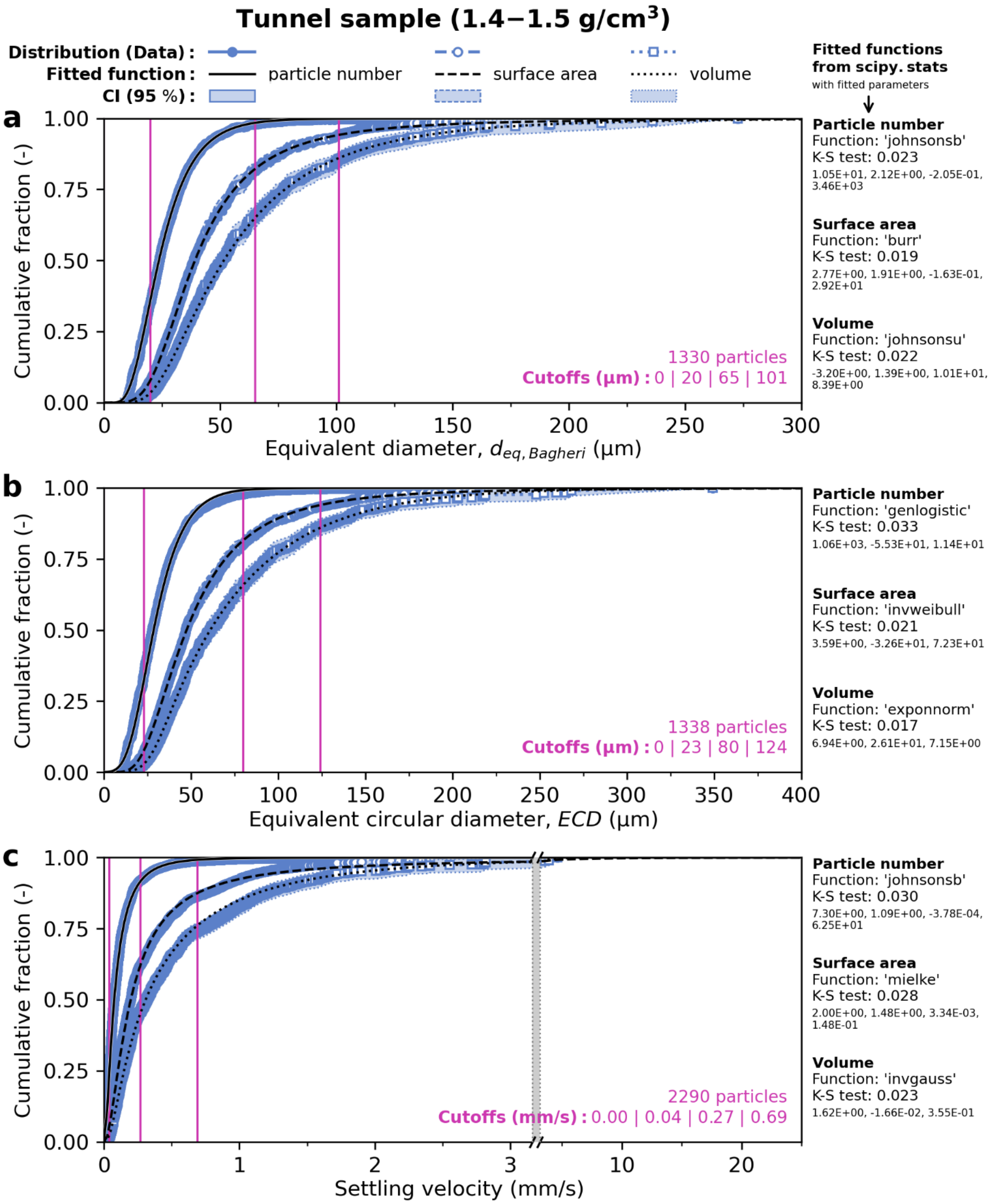

Figure S54. Cumulative distributions of particle size as equivalent diameter (a) or equivalent circular diameter (b) and settling velocity (c) composed from measurements for fraction 1.4–1.5 g/cm³ of the Tunnel sample. Cumulative particle number, surface area and volume shown, respectively, including 95% confidence interval derived via bootstrapping (N=2000) and best fit continuous distribution function from Python package *scipy.stats* (function name[8], K-S test statistic and fitted parameters given on the right-hand side). Note computed cutoffs indicated in pink (cf. section S3).

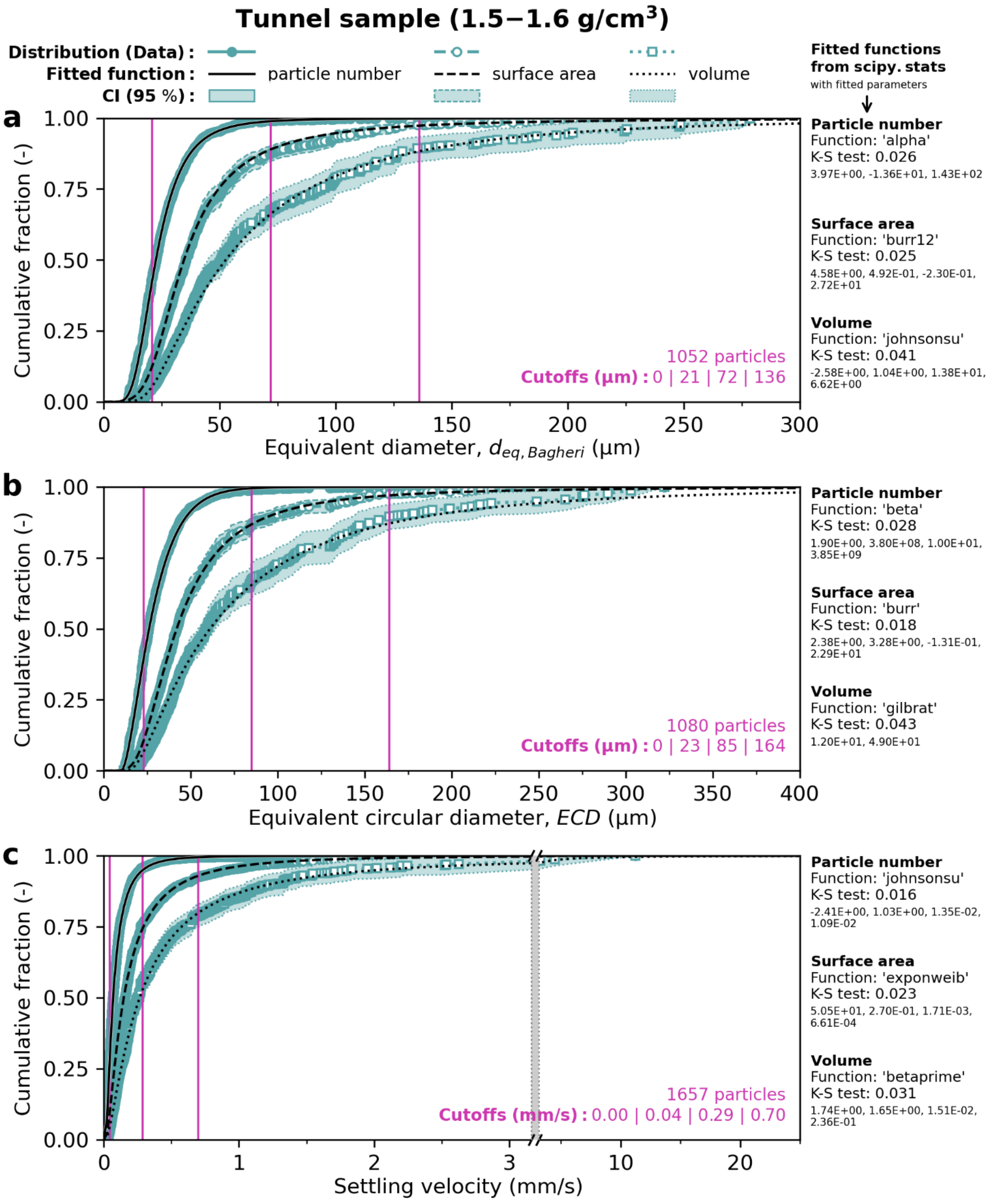

Figure S55. Cumulative distributions of particle size as equivalent diameter (a) or equivalent circular diameter (b) and settling velocity (c) composed from measurements for fraction 1.5–1.6 g/cm³ of the Tunnel sample. Cumulative particle number, surface area and volume shown, respectively, including 95% confidence interval derived via bootstrapping (N=2000) and best fit continuous distribution function from Python package *scipy.stats* (function name[8], K-S test statistic and fitted parameters given on the right-hand side). Note computed cutoffs indicated in pink (cf. section S3).

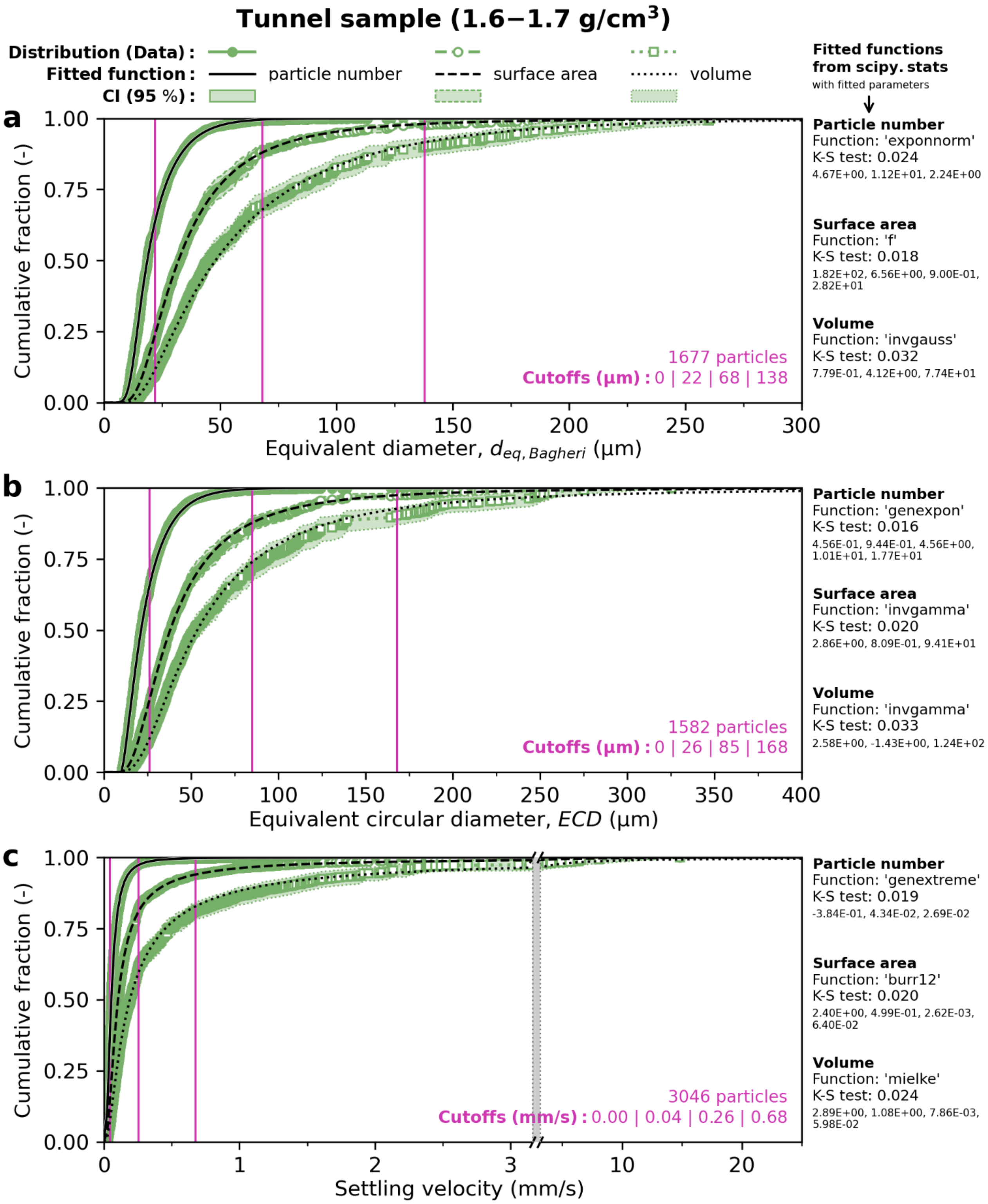

Figure S56. Cumulative distributions of particle size as equivalent diameter (a) or equivalent circular diameter (b) and settling velocity (c) composed from measurements for fraction 1.6–1.7 g/cm³ of the Tunnel sample. Cumulative particle number, surface area and volume shown, respectively, including 95% confidence interval derived via bootstrapping (N=2000) and best fit continuous distribution function from Python package *scipy.stats* (function name[8], K-S test statistic and fitted parameters given on the right-hand side). Note computed cutoffs indicated in pink (cf. section S3).

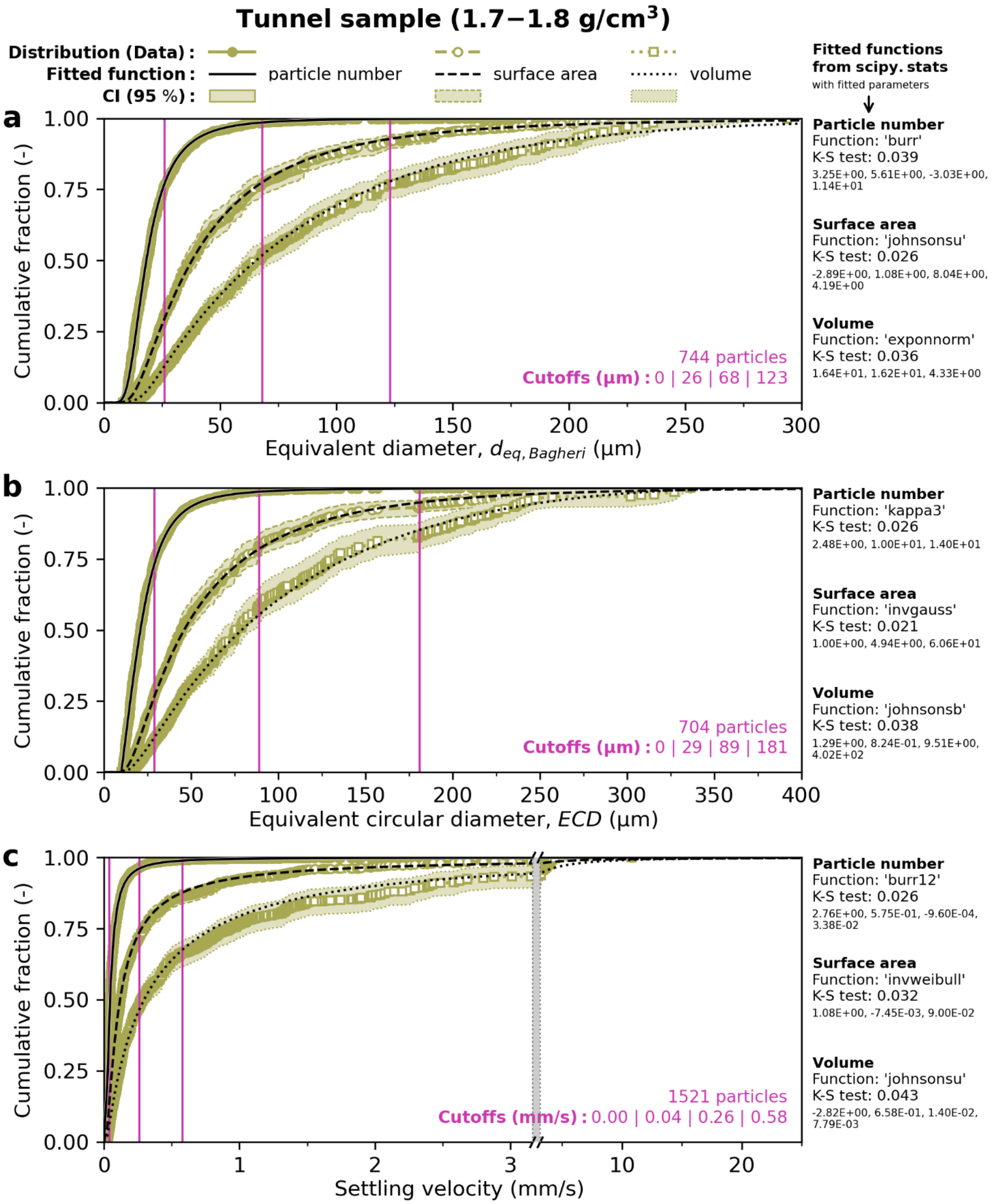

Figure S57. Cumulative distributions of particle size as equivalent diameter (a) or equivalent circular diameter (b) and settling velocity (c) composed from measurements for fraction 1.7–1.8 g/cm³ of the Tunnel sample. Cumulative particle number, surface area and volume shown, respectively, including 95% confidence interval derived via bootstrapping (N=2000) and best fit continuous distribution function from Python package *scipy.stats* (function name[8], K-S test statistic and fitted parameters given on the right-hand side). Note computed cutoffs indicated in pink (cf. section S3).

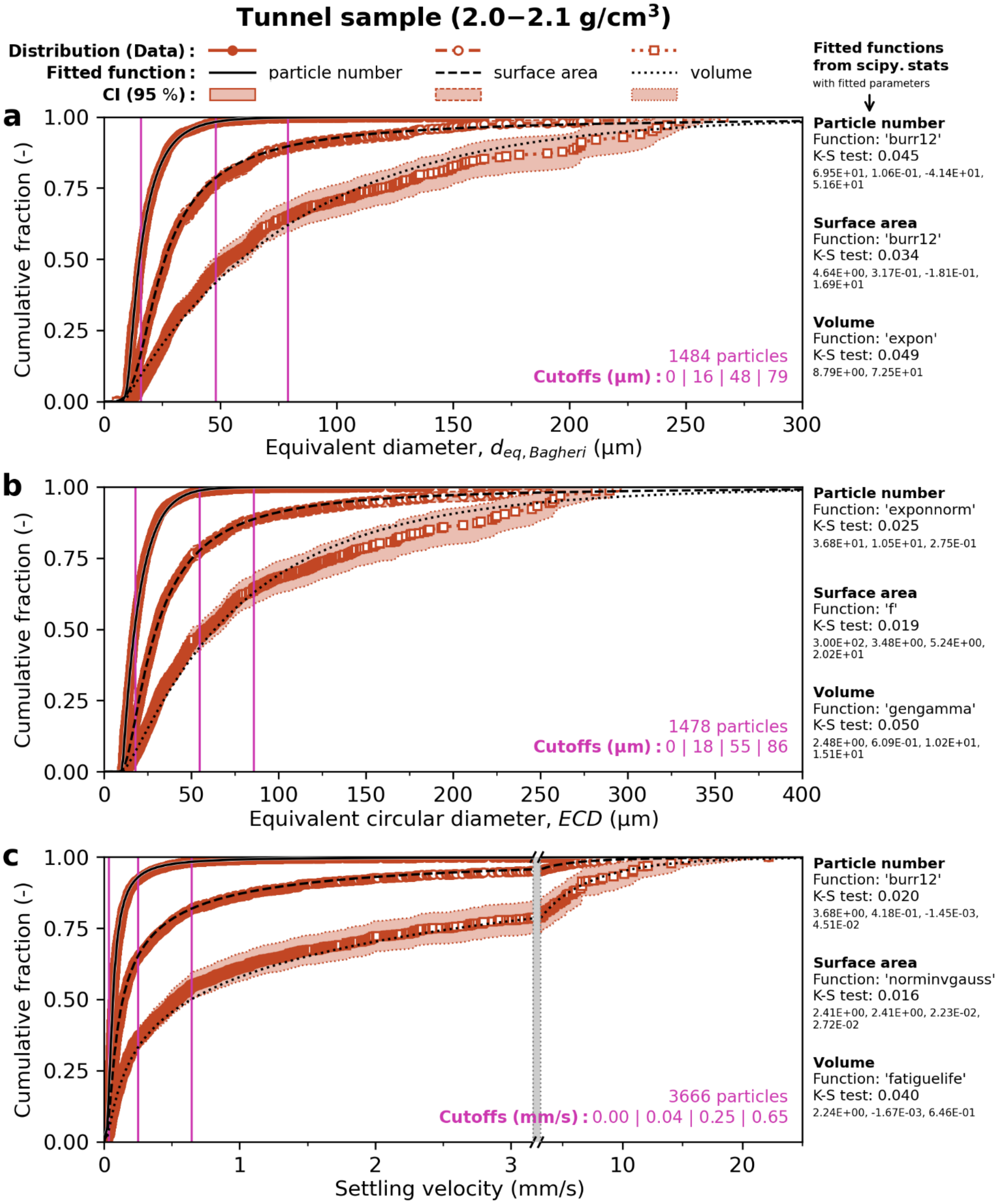

Figure S58. Cumulative distributions of particle size as equivalent diameter (a) or equivalent circular diameter (b) and settling velocity (c) composed from measurements for fraction 2.0–2.1 g/cm³ of the Tunnel sample. Cumulative particle number, surface area and volume shown, respectively, including 95% confidence interval derived via bootstrapping (N=2000) and best fit continuous distribution function from Python package *scipy.stats* (function name[8], K-S test statistic and fitted parameters given on the right-hand side). Note computed cutoffs indicated in pink (cf. section S3).

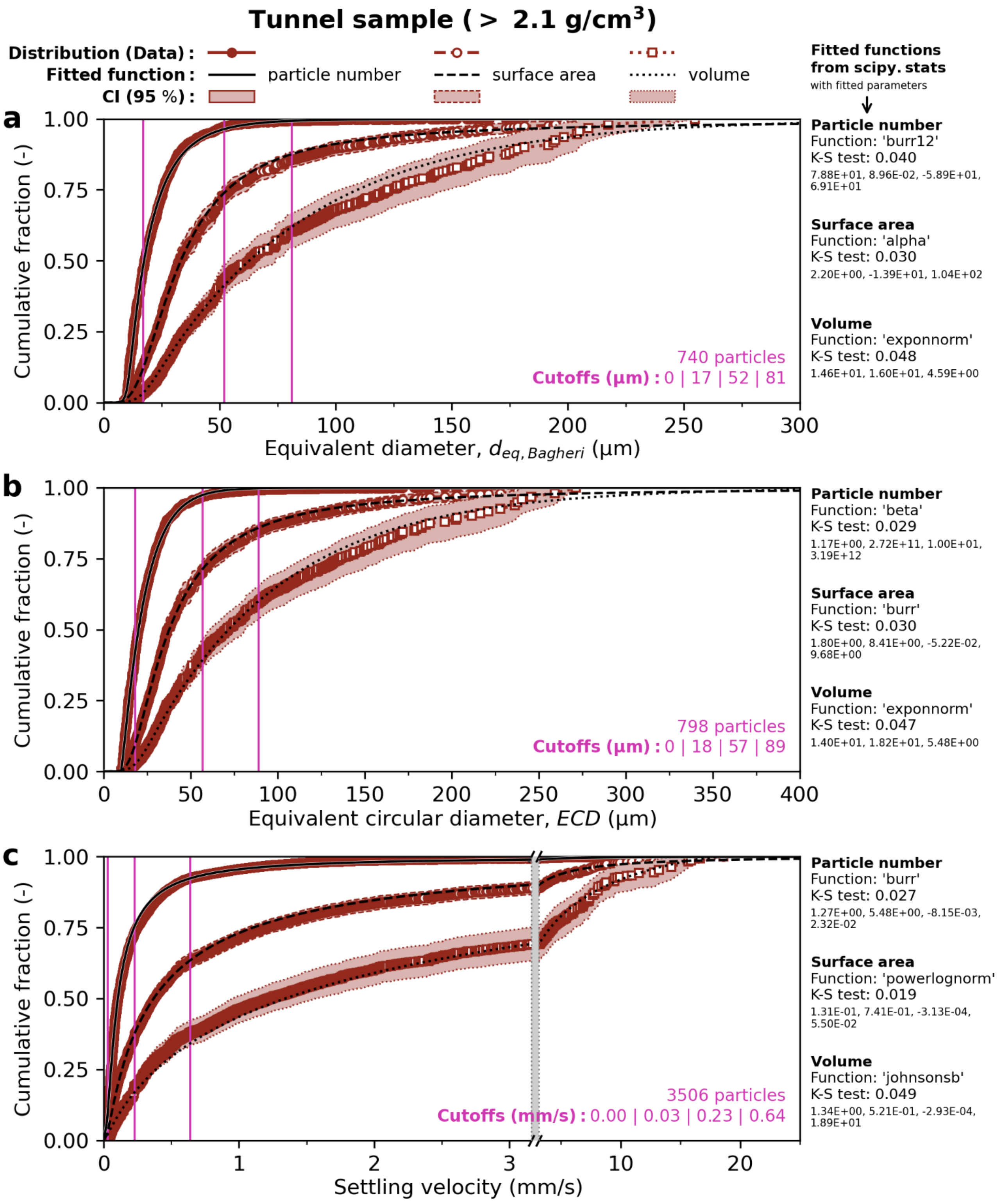

Figure S59. Cumulative distributions of particle size as equivalent diameter (a) or equivalent circular diameter (b) and settling velocity (c) composed from measurements for fraction > 2.1 g/cm³ of the Tunnel sample. Cumulative particle number, surface area and volume shown, respectively, including 95% confidence interval derived via bootstrapping (N=2000) and best fit continuous distribution function from Python package *scipy.stats* (function name[8], K-S test statistic and fitted parameters given on the right-hand side). Note computed cutoffs indicated in pink (cf. section S3).

S11 Composed particle size and settling velocity distributions

To elucidate size and settling velocities of TRWP from both samples, respectively, cumulative distributions of those properties were derived. In order to achieve this, the measured distributions of the different density fractions (see section S10) were merged – either with respect to tire material (Figures S60, S62, S64 and S66) or TRWP content (Figures S61, S63, S65 and S67). The entire methodology is described in section S6. Different distributions were composed related to particle mass (Figures S60 and S61), particle volume (Figures S62 and S63), particle surface area (Figures S64 and S65) as well as particle count (Figures S66 and S67). Associated quartiles of these cumulative distributions are comprised in Table S8, while Table S9 specifies the best fit cumulative distribution functions (CDFs) from the Python package *scipy.stats*[7] that are included in Figures S60–S67, respectively. The best fit CDFs were determined by successively fitting and comparing the available functions from *scipy.stats*[7,8] according to least squares (R^2). To ensure an equally good fit along the whole data range despite a decreasing quantity of empirical data with increasing particle size, fitting data was generated more evenly. For this purpose, 5000 samples of the cumulative probability (between 0 and 1) were linearly interpolated between the adjacent empirical data points, respectively.

As discussed in the main manuscript, the presented cumulated distributions might be used as inputs for different modeling scenarios, especially to sample particle properties in case of probabilistic modeling[14]. Yet, the presented distributions have to be handled and contextualized carefully (please also consider respective discussion in the main manuscript). In case of the Tunnel sample, only the cumulative distributions derived with respect to incorporated tire mass include all size fractions, while all other distributions could only be composed exclusively for the 20–250 µm size fraction. For the Road simulator sample, distributions might be truncated on the lower end, because particles with ECD < 10 µm are not captured due to limitations of the employed imaging setup and the according method for measuring settling velocities. This is especially important for distributions that were derived with respect to surface area and particle count, since these parameters are increasingly dominated by the smallest particles of a population. Similar truncations are less probable for the Tunnel sample, since only the 20–250 µm size fraction was investigated. Still, this fraction includes a certain amount of smaller particles due to imperfect size fractionation via sieving as was discussed in the main publication.

Finally, the presented settling velocity distributions are only valid for pure water at 15 °C. For use under deviating conditions, they have to be corrected with respect to changes in water temperature or salinity. Before merging distributions (cf. section S6), such corrections should be applied individually to each density fraction's cumulative settling velocity distribution (e.g. by deriving a correction factor according to Stokes' law when applied to reference and target properties, respectively). A respective script and the required raw data are included in the Zenodo repository[12].

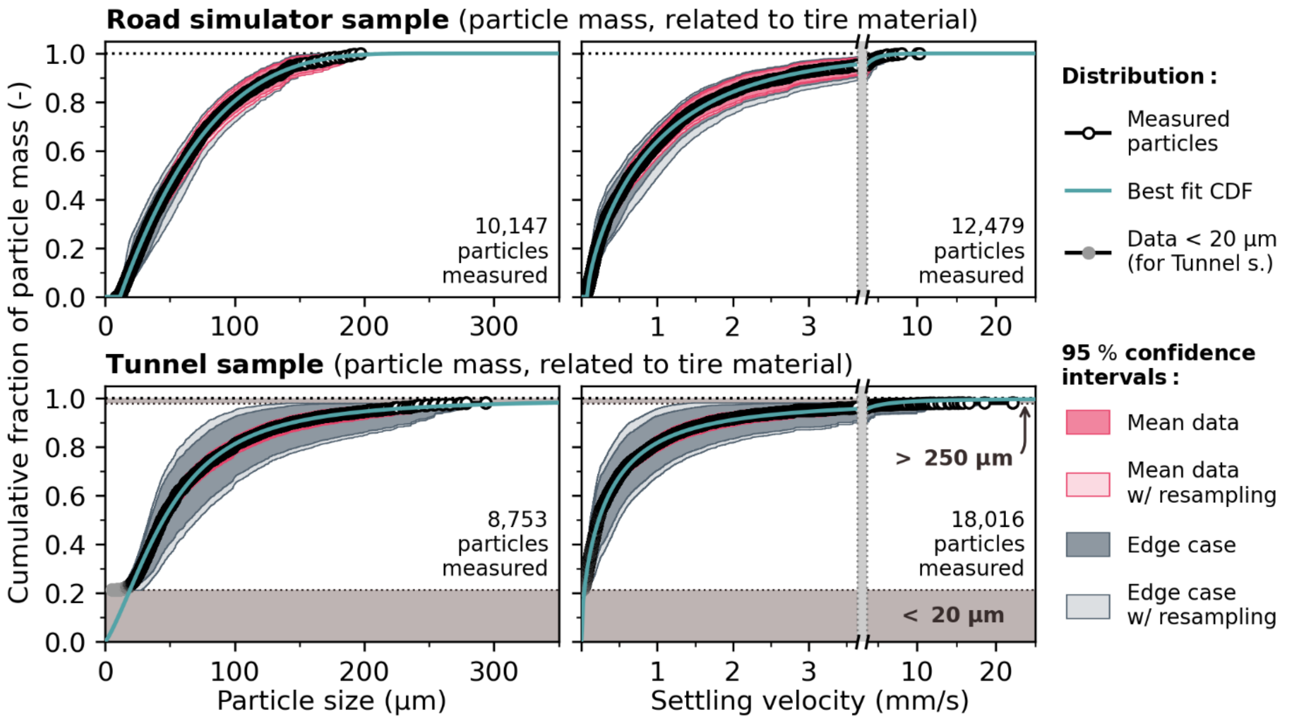

Figure S60. Cumulative distributions of particle size and settling velocity (in water at 15 °C) with respect to particle mass. Distributions were derived with regard to TRWP-incorporated tire material by composing settling data on density fractions of both samples based on estimated tire contents, respectively.

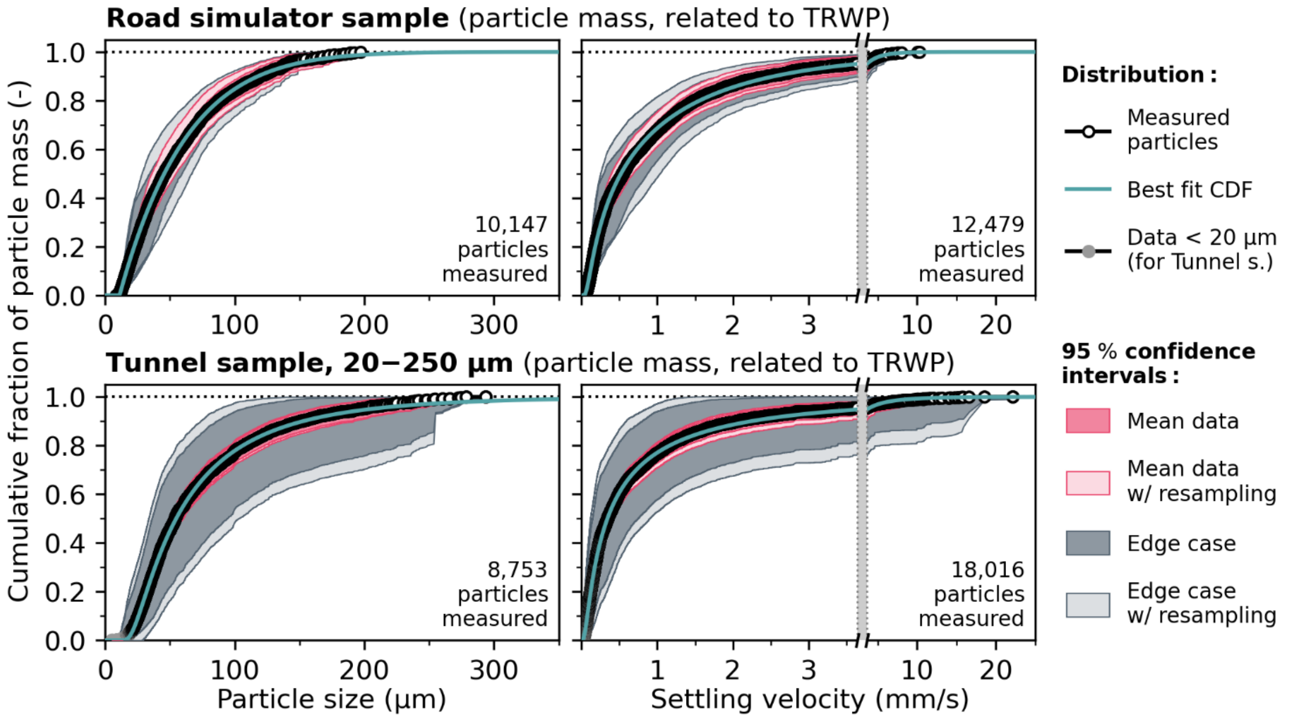

Figure S61. Cumulative distributions of particle size and settling velocity (in water at 15 °C) with respect to particle mass. Distributions were derived with regard to TRWP by composing settling data on density fractions of both samples based on estimated TRWP contents, respectively. For the Tunnel sample, only the 20–250 µm size fraction could be considered.

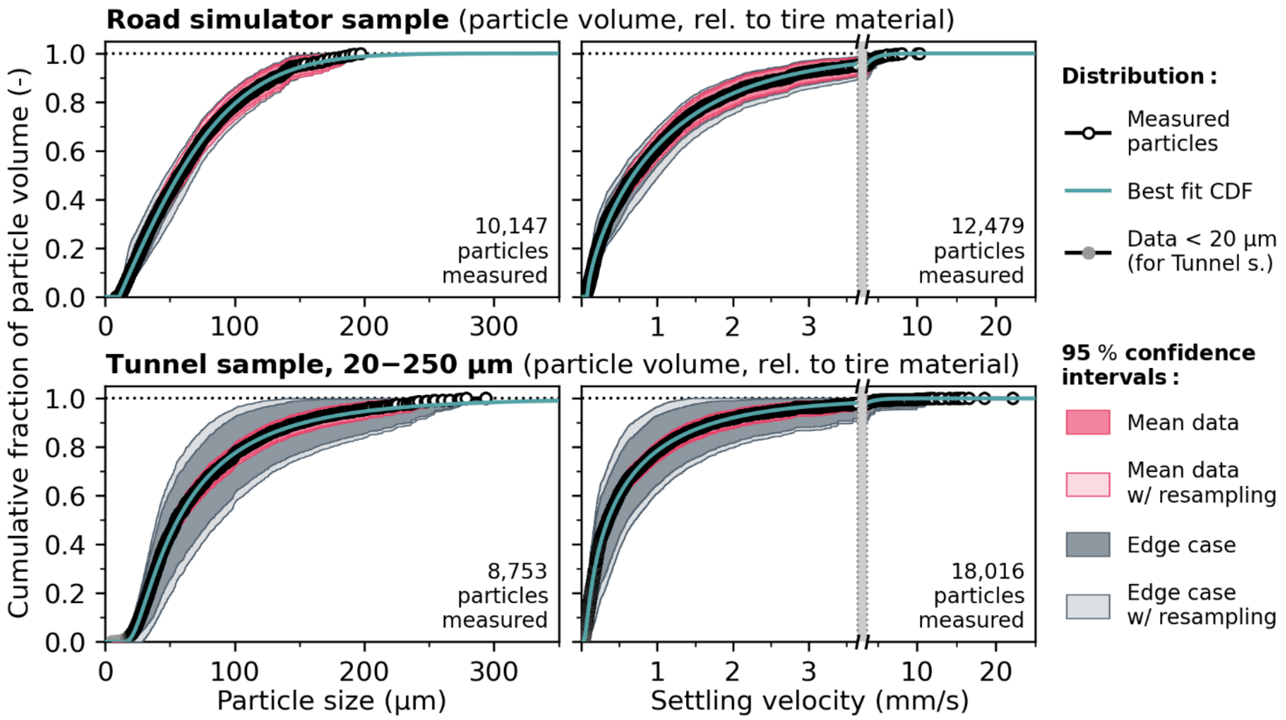

Figure S62. Cumulative distributions of particle size and settling velocity (in water at 15 °C) with respect to particle volume. Distributions were derived with regard to TRWP-incorporated tire material by composing settling data on density fractions of both samples based on estimated tire contents, respectively. For the Tunnel sample, only the 20–250 µm size fraction could be considered.

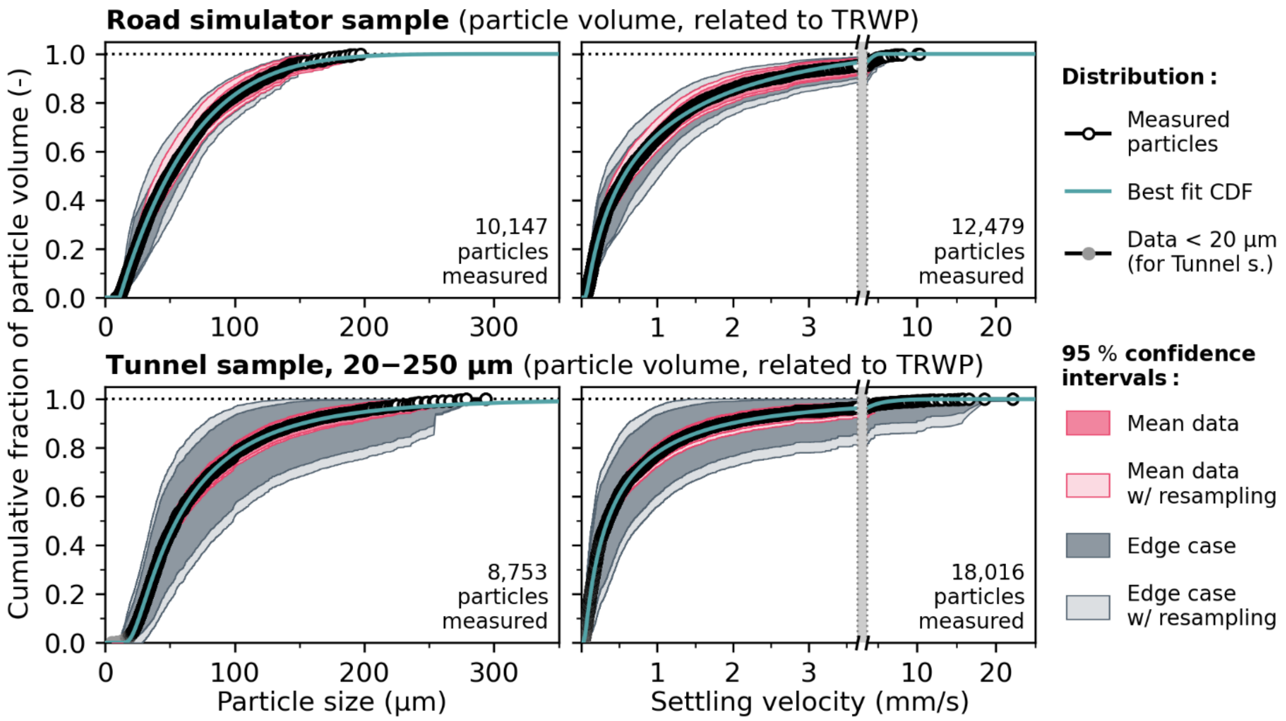

Figure S63. Cumulative distributions of particle size and settling velocity (in water at 15 °C) with respect to particle volume. Distributions were derived with regard to TRWP by composing settling data on density fractions of both samples based on estimated TRWP contents, respectively. For the Tunnel sample, only the 20–250 µm size fraction could be considered.

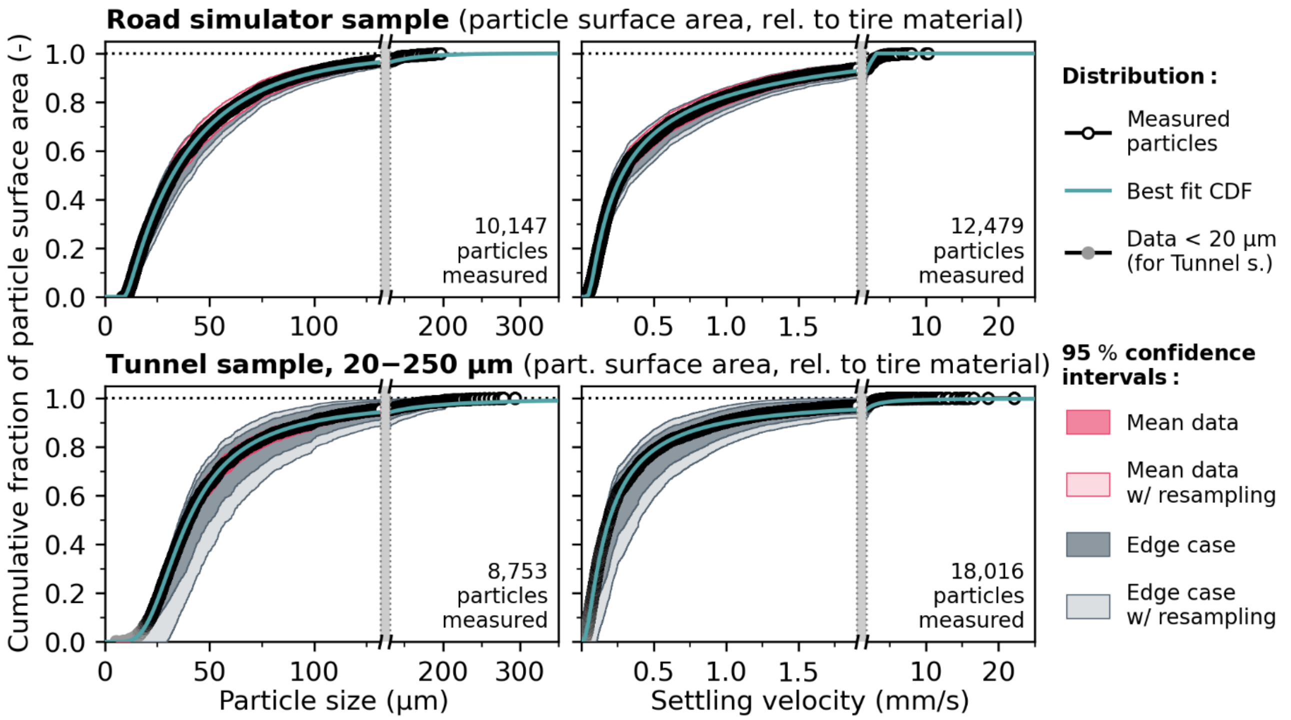

Figure S64. Cumulative distributions of particle size and settling velocity (in water at 15 °C) with respect to particle surface area. Distributions were derived with regard to TRWP-incorporated tire material by composing settling data on density fractions of both samples based on estimated tire contents, respectively. For the Tunnel sample, only the 20–250 µm size fraction could be considered.

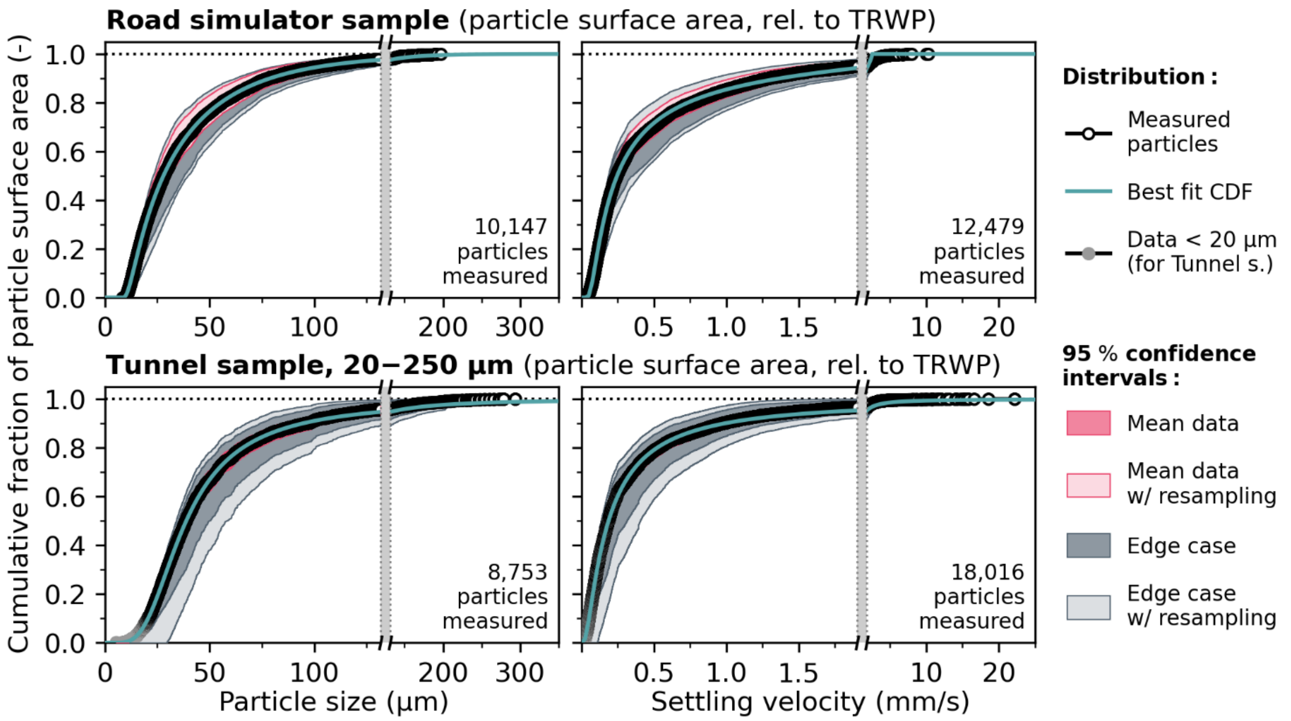

Figure S65. Cumulative distributions of particle size and settling velocity (in water at 15 °C) with respect to particle surface area. Distributions were derived with regard to TRWP by composing settling data on density fractions of both samples based on estimated TRWP contents, respectively. For the Tunnel sample, only the 20–250 µm size fraction could be considered.

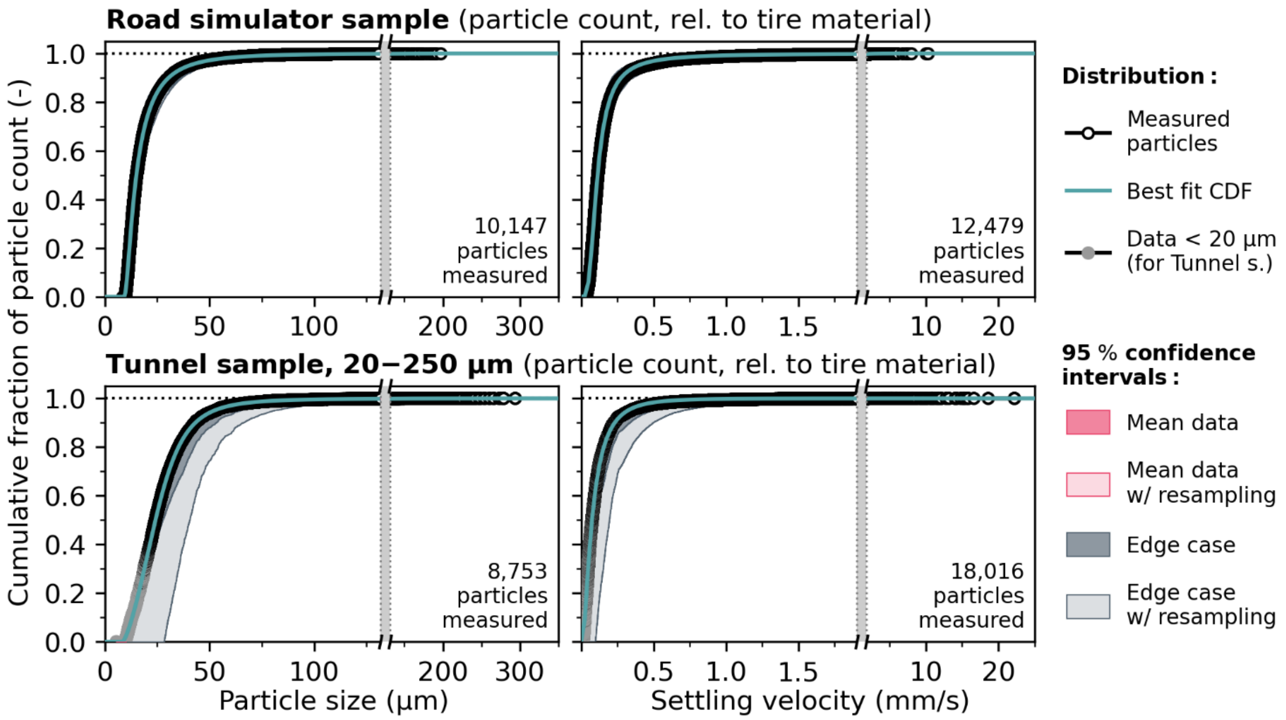

Figure S66. Cumulative distributions of particle size and settling velocity (in water at 15 °C) with respect to particle count. Distributions were derived with regard to TRWP-incorporated tire material by composing settling data on density fractions of both samples based on estimated tire contents, respectively. For the Tunnel sample, only the 20–250 µm size fraction could be considered.

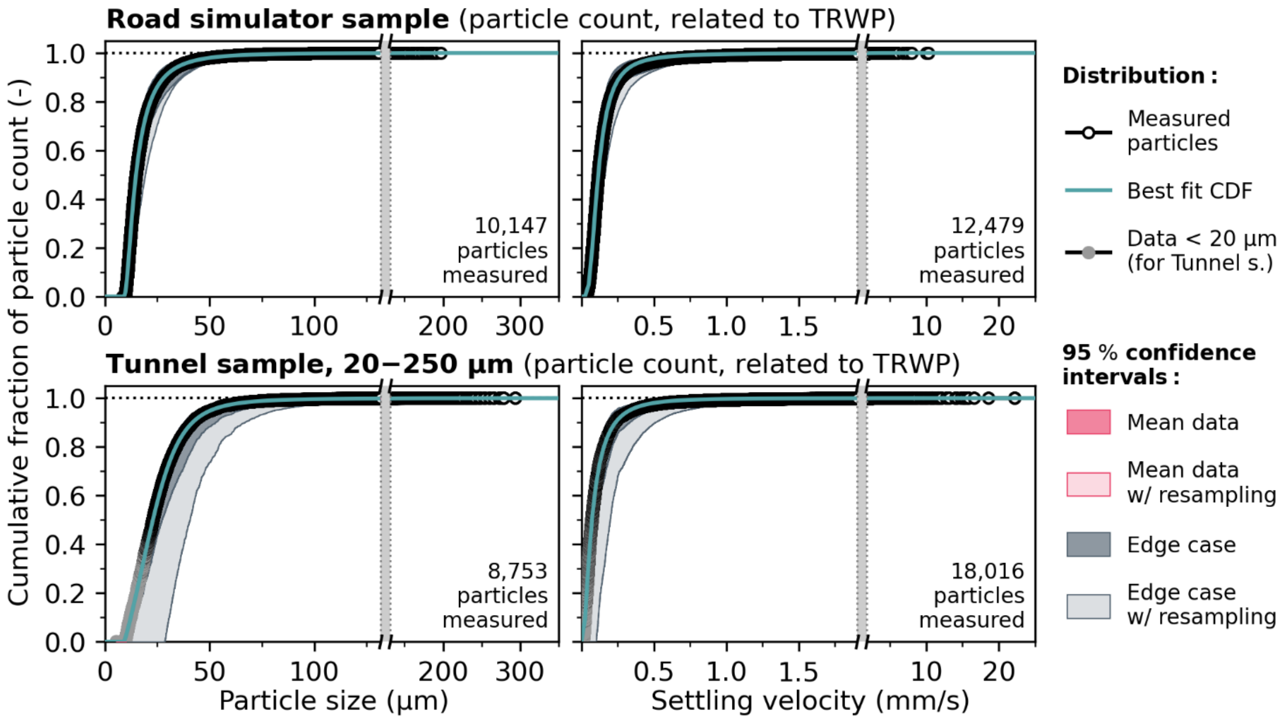

Figure S67. Cumulative distributions of particle size and settling velocity (in water at 15 °C) with respect to particle count. Distributions were derived with regard to TRWP by composing settling data on density fractions of both samples based on estimated TRWP contents, respectively. For the Tunnel sample, only the 20–250 µm size fraction could be considered.

Table S8. Quartiles of all cumulative particle size and settling velocity distributions as composed for both samples (cf. Figure S60–Figure S67). Please note the restriction to the 20–250 µm size fraction for most instances of the Tunnel sample (annotated).

Figure and distribution	Quantile	Particle size (µm)		Settling velocity (mm/s)	
		Road simulator sample	Tunnel sample	Road simulator sample	Tunnel sample
**Figure 60** mass + tire	Q1: 25%	30.3	22.3	0.25	0.06
	Q2: 50%	54.4	43.7	0.65	0.22
	Q3: 75%	88.6	84.2	1.41	0.70
**Figure 61** mass + TRWP	Q1: 25%	25.9	35.5*	0.21	0.15*
	Q2: 50%	45.4	54.1*	0.54	0.35*
	Q3: 75%	77.9	95.2*	1.25	0.93*
**Figure 62** volume + tire	Q1: 25%	31.9	36.6*	0.26	0.15*
	Q2: 50%	57.1	55.1*	0.69	0.35*
	Q3: 75%	91.8	97.0*	1.45	0.89*
**Figure 63** volume + TRWP	Q1: 25%	27.3	35.9*	0.22	0.15*
	Q2: 50%	48.7	54.3*	0.58	0.35*
	Q3: 75%	81.9	95.2*	1.31	0.90*
**Figure 64** surf. area + tire	Q1: 25%	19.4	28.5*	0.14	0.10*
	Q2: 50%	32.1	39.8*	0.28	0.19*
	Q3: 75%	56.7	60.7*	0.73	0.44*
**Figure 65** surf. area + TRWP	Q1: 25%	17.7	27.6*	0.13	0.09*
	Q2: 50%	28.0	38.8*	0.24	0.19*
	Q3: 75%	48.3	58.9*	0.60	0.42*
**Figure 66** count + tire	Q1: 25%	12.2	17.7*	0.08	0.04*
	Q2: 50%	15.0	24.1*	0.11	0.07*
	Q3: 75%	20.7	32.7*	0.17	0.13*
**Figure 67** count + TRWP	Q1: 25%	12.2	16.5*	0.08	0.04*
	Q2: 50%	14.8	23.0*	0.12	0.07*
	Q3: 75%	19.9	31.4*	0.17	0.13*

* distribution and associated quartiles are restricted to the 20–250 µm size fraction of the Tunnel sample

Table S9. Best-fit cumulative distribution functions (CDF) from *scipy.stats*[7] Python package as depicted in Figure S60–Figure S67 for particle size and settling velocity distributions. Name of function and respectively fitted parameters given.

Figure and references	Sample and Quantity		Best-fit cumulative distribution function (CDF) from *scipy.stats*[7]						
			Name of CDF[8]	Fitted parameters of CDF					
				P1	P2	P3	P4	P5	P6
**Figure 60** mass + tire	Road simulator	d_{eq}	*genpareto*	-2.77E-01	1.15E+01	6.74E+01	-	-	-
		w_s	*exponweib*	7.53E-01	9.39E-01	7.47E-02	1.18E+00	-	-
	Tunnel	d_{eq}	*burr*	2.12E+00	5.63E-01	-7.95E-12	6.83E+01	-	-
		w_s	*mielke*	5.18E-01	1.35E+00	1.29E-09	7.60E-01	-	-
**Figure 61** mass + TRWP	Road simulator	d_{eq}	*halfgennorm*	1.71E+00	1.09E+01	9.26E+01	-	-	-
		w_s	*kappa4*	1.43E+00	2.78E-02	-4.01E-01	1.34E+00	-	-
	Tunnel (20-250 µm)	d_{eq}	*invgauss*	9.89E-01	1.09E+01	6.73E+01	-	-	-
		w_s	*fatiguelife*	1.45E+00	1.72E-02	3.31E-01	-	-	-
**Figure 62** volume + tire	Road simulator	d_{eq}	*recipinvgauss*	1.75E+00	8.18E+00	2.30E+01	-	-	-
		w_s	*gausshyper*	8.70E+01	8.80E-01	8.77E+01	2.72E+03	2.45E-02	3.22E+00
	Tunnel (20-250 µm)	d_{eq}	*burr12*	2.91E+00	6.20E-01	7.48E+00	2.53E+01	-	-
		w_s	*betaprime*	2.88E+00	1.31E+00	8.37E-03	7.20E-02	-	-
**Figure 63** volume + TRWP	Road simulator	d_{eq}	*exponweib*	3.31E+01	3.18E-01	8.82E+00	8.79E-02	-	-
		w_s	*burr12*	3.37E+00	5.76E-01	6.73E-03	8.23E-02	-	-
	Tunnel (20-250 µm)	d_{eq}	*mielke*	1.38E+00	3.45E+00	8.47E+00	2.46E+01	-	-
		w_s	*powerlognorm*	8.01E-01	8.39E-01	2.90E-03	6.03E-02	-	-
**Figure 64** surf. area + tire	Road simulator	d_{eq}	*exponnorm*	2.62E+01	1.14E+01	1.71E+00	-	-	-
		w_s	*gausshyper*	6.10E+01	4.27E+00	6.09E+01	1.41E+04	4.87E-02	1.07E+01
	Tunnel (20-250 µm)	d_{eq}	*invgauss*	1.03E+00	9.67E+00	6.57E+01	-	-	-
		w_s	*invgauss*	4.08E+00	-5.14E-03	2.56E-01	-	-	-
**Figure 65** surf. area + TRWP	Road simulator	d_{eq}	*genpareto*	-1.57E-01	1.12E+01	5.69E+01	-	-	-
		w_s	*johnsonsb*	1.35E+00	6.06E-01	5.21E-02	5.29E+00	-	-
	Tunnel (20-250 µm)	d_{eq}	*invgauss*	9.98E-01	1.04E+01	6.62E+01	-	-	-
		w_s	*johnsonsb*	2.50E+00	6.58E-01	3.12E-02	1.44E+01	-	-
**Figure 66** count + tire	Road simulator	d_{eq}	*fatiguelife*	1.04E+00	7.41E+00	2.06E+01	-	-	-
		w_s	*gausshyper*	4.95E+01	5.55E-01	5.05E+01	8.72E+02	2.15E-02	2.57E+00
	Tunnel (20-250 µm)	d_{eq}	*burr12*	3.09E+00	6.07E-01	5.38E+00	2.62E+01	-	-
		w_s	*betaprime*	3.03E+00	1.28E+00	6.83E-03	6.46E-02	-	-
**Figure 67** count + TRWP	Road simulator	d_{eq}	*exponweib*	3.90E+01	3.33E-01	8.61E+00	9.46E-02	-	-
		w_s	*burr*	2.31E+00	1.43E+00	1.55E-02	8.17E-02	-	-
	Tunnel (20-250 µm)	d_{eq}	*mielke*	1.14E+00	3.39E+00	9.16E+00	2.44E+01	-	-
		w_s	*powerlognorm*	1.93E-01	4.78E-01	-1.01E-03	2.94E-02	-	-

S12 Particle aspect ratios obtained from settling data

Figure S68 comprises the cumulative distributions of aspect ratios as derived from settling data with respect to estimated particle volume. Aspect ratios were determined as the ratio between the minimum and maximum edge length of rotated bounding rectangles fitted to each captured contour of a particle. Distributions for all investigated density fraction of both samples are shown, respectively – similar to distributions of particle size and settling velocity as depicted in Figure 3 of the main manuscript. Regarding the Tunnel sample, significantly higher median aspect ratios were measured for particles from the density fractions below 1.7 g/cm³, which might correspond to the elongated shape associated with typical TRWP.[9]

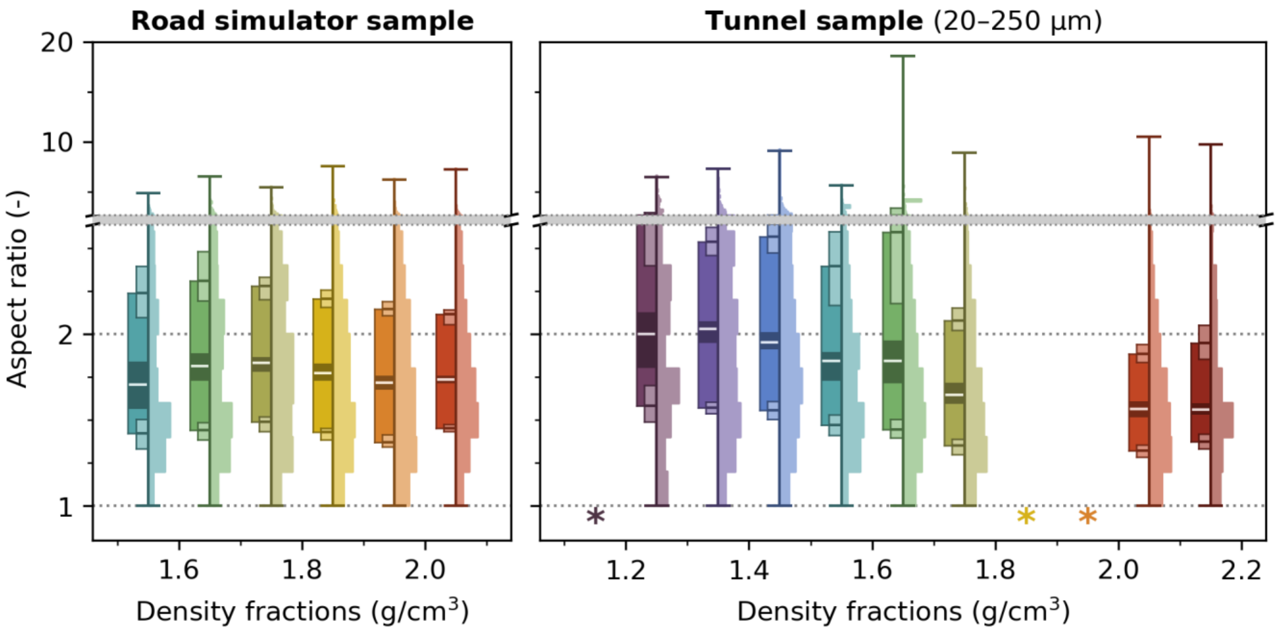

Figure S68. Cumulative distributions of particle aspect ratios with respect to estimated particle volumes for density fractions of both samples. The dual plots indicate histograms (right-hand half) as well as boxplots with range, quartiles and associated 95% confidence intervals (left-hand half) as measured for the density fractions of the Road simulator sample (right panels) and of the 20–250 µm size fraction of the Tunnel sample (center panels), respectively. Fractions that were not analyzed due to insufficient sample mass are annotated (*).

S13 References

(1) Kovochich, M.; Liong, M.; Parker, J. A.; Oh, S. C.; Lee, J. P.; Xi, L.; Kreider, M. L.; Unice, K. M. Chemical mapping of tire and road wear particles for single particle analysis. *Sci. Total Environ.* **2021**, *757*, 144085. DOI: 10.1016/j.scitotenv.2020.144085. Published Online: Nov. 26, 2020.

(2) Bagheri, G. H.; Bonadonna, C.; Manzella, I.; Vonlanthen, P. On the characterization of size and shape of irregular particles. *Powder Technol.* **2015**, *270*, 141–153. DOI: 10.1016/j.powtec.2014.10.015.

(3) Dittmar, S.; Ruhl, A. S.; Altmann, K.; Jekel, M. Settling Velocities of Small Microplastic Fragments and Fibers. *Environ. Sci. Technol.* **2024**.

(4) Chakraborti, R. K.; Atkinson, J. F.; van Benschoten, J. E. Characterization of Alum Floc by Image Analysis. *Environ. Sci. Technol.* **2000**, *34* (18), 3969–3976. DOI: 10.1021/es990818o.

(5) Shen, X.; Maa, J. P.-Y. A camera and image processing system for floc size distributions of suspended particles. *Mar. Geol.* **2016**, *376*, 132–146. DOI: 10.1016/j.margeo.2016.03.009.

(6) Dittmar, S.; Ruhl, A. S.; Jekel, M. Optimized and Validated Settling Velocity Measurement for Small Microplastic Particles (10-400 µm). *ACS ES&T Water* **2023.** DOI: 10.1021/acsestwater.3c00457.

(7) Virtanen, P.; Gommers, R.; Oliphant, T. E.; Haberland, M.; Reddy, T.; Cournapeau, D.; Burovski, E.; Peterson, P.; Weckesser, W.; Bright, J.; van der Walt, S. J.; Brett, M.; Wilson, J.; Millman, K. J.; Mayorov, N.; Nelson, A. R. J.; Jones, E.; Kern, R.; Larson, E.; Carey, C. J.; Polat, İ.; Feng, Y.; Moore, E. W.; VanderPlas, J.; Laxalde, D.; Perktold, J.; Cimrman, R.; Henriksen, I.; Quintero, E. A.; Harris, C. R.; Archibald, A. M.; Ribeiro, A. H.; Pedregosa, F.; van Mulbregt, P. SciPy 1.0: fundamental algorithms for scientific computing in Python. *Nat. Methods* **2020**, *17* (3), 261–272. DOI: 10.1038/s41592-019-0686-2. Published Online: Feb. 3, 2020.

(8) The SciPy community. *Documentation (scipy.stats)*. https://docs.scipy.org/doc/scipy/reference/stats.html (accessed 2024-01-06).

(9) Wagner, S.; Hüffer, T.; Klöckner, P.; Wehrhahn, M.; Hofmann, T.; Reemtsma, T. Tire wear particles in the aquatic environment - A review on generation, analysis, occurrence, fate and effects. *Water Res.* **2018**, *139*, 83–100. DOI: 10.1016/j.watres.2018.03.051.

(10) Kreider, M. L.; Panko, J. M.; McAtee, B. L.; Sweet, L. I.; Finley, B. L. Physical and chemical characterization of tire-related particles: comparison of particles generated using different methodologies. *Sci. Total Environ.* **2010**, *408* (3), 652–659. DOI: 10.1016/j.scitotenv.2009.10.016.

(11) Unice, K. M.; Weeber, M. P.; Abramson, M. M.; Reid, R. C. D.; van Gils, J. A. G.; Markus, A. A.; Vethaak, A. D.; Panko, J. M. Characterizing export of land-based microplastics to the estuary - Part I: Application of integrated geospatial microplastic transport models to assess tire and road wear particles in the Seine watershed. *Sci. Total Environ.* **2019**, *646*, 1639–1649. DOI: 10.1016/j.scitotenv.2018.07.368.

(12) Dittmar, S.; Weyrauch, S. Additional data for “Settling velocities of tire and road wear particles: Analyzing finely graded density fractions of samples from a road simulator and a highway tunnel” **2025.** DOI: 10.5281/zenodo.15088431.

(13) Kovochich, M.; Parker, J. A.; Oh, S. C.; Lee, J. P.; Wagner, S.; Reemtsma, T.; Unice, K. M. Characterization of Individual Tire and Road Wear Particles in Environmental Road Dust, Tunnel Dust, and Sediment. *Environ. Sci. Technol. Lett.* **2021**, *8* (12), 1057–1064. DOI: 10.1021/acs.estlett.1c00811.

(14) Kooi, M.; Koelmans, A. A. Simplifying Microplastic via Continuous Probability Distributions for Size, Shape, and Density. *Environ. Sci. Technol. Lett.* **2019**, *6* (9), 551–557. DOI: 10.1021/acs.estlett.9b00379.
